# Modular Synthesis of PEG-Dendritic Block Copolymers by Thermal Azide–Alkyne Cycloaddition with Internal Alkynes and Evaluation of their Self-Assembly for Drug Delivery Applications

**DOI:** 10.1021/acs.biomac.3c01429

**Published:** 2024-04-13

**Authors:** Samuel Parcero-Bouzas, Juan Correa, Celia Jimenez-Lopez, Bruno Delgado Gonzalez, Eduardo Fernandez-Megia

**Affiliations:** Centro Singular de Investigación en Química Biolóxica e Materiais Moleculares (CIQUS), Departamento de Química Orgánica, Universidade de Santiago de Compostela, Jenaro de la Fuente s/n, Santiago de Compostela 15782, Spain

## Abstract

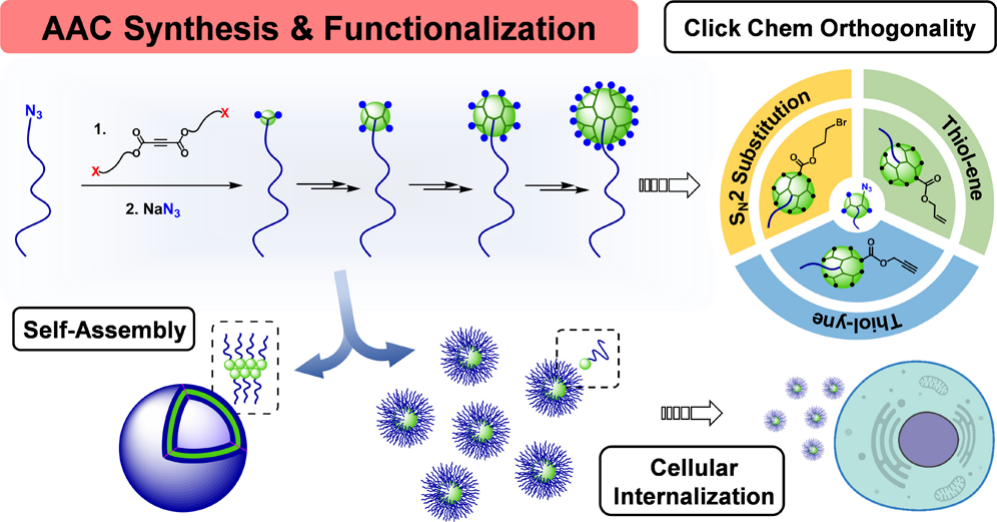

Linear–dendritic block copolymers assemble in solution due to differences in the solubility or charge properties of the blocks. The monodispersity and multivalency of the dendritic block provide unparalleled control for the design of drug delivery systems when incorporating poly(ethylene glycol) (PEG) as a linear block. An accelerated synthesis of PEG-dendritic block copolymers based on the click and green chemistry pillars is described. The tandem composed of the thermal azide–alkyne cycloaddition with internal alkynes and azide substitution is revealed as a flexible, reliable, atom-economical, and user-friendly strategy for the synthesis and functionalization of biodegradable (polyester) PEG-dendritic block copolymers. The high orthogonality of the sequence has been exploited for the preparation of heterolayered copolymers with terminal alkenes and alkynes, which are amenable for subsequent functionalization by thiol–ene and thiol–yne click reactions. Copolymers with tunable solubility and charge were so obtained for the preparation of various types of nanoassemblies with promising applications in drug delivery.

## Introduction

Linear–dendritic block copolymers are interesting materials with the ability to assemble in solution due to differences in the solubility or charge properties of the blocks. The monodispersity and globular nature of the dendritic block, along with the possibility of tuning its multivalency with the generation (G), grant linear–dendritic copolymers an unparalleled control among block copolymers.^1−5^ Originally described in the early 1990s, linear–dendritic block copolymers have found wide application in the biomedical field, particularly for the design of drug delivery systems (DDS) when incorporating poly(ethylene glycol) (PEG).^6^ PEG, an FDA-approved hydrophilic polymer, is characterized by a reduced toxicity and immunogenicity as well as a stealth character for increased solubility and circulation times in the bloodstream.^7^

**Figure 1:**
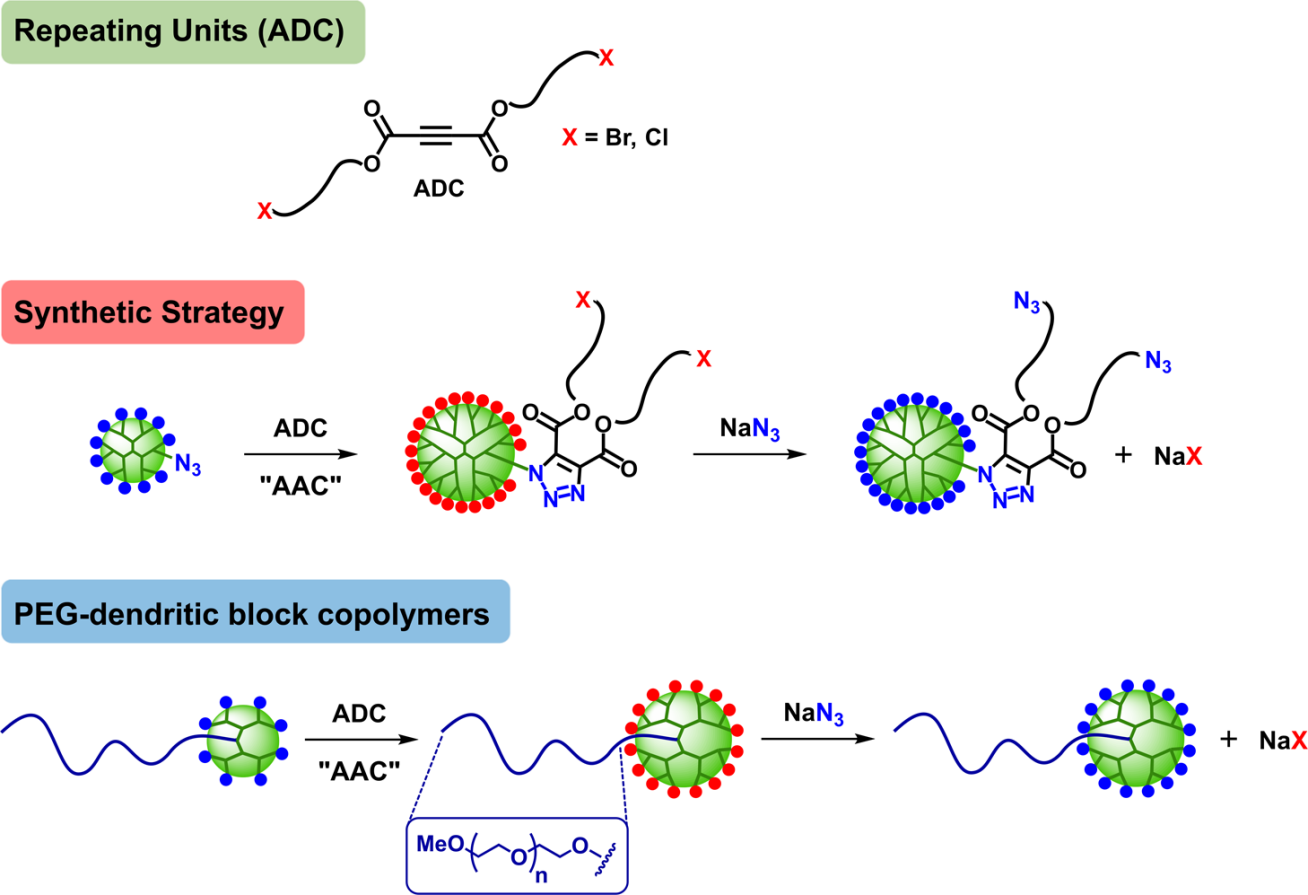
General structure of the acetylenedicarboxylate (ADC) repeating units. Synthesis of dendrimers based on the thermal azide–alkyne cycloaddition (AAC) and azide substitution reactions. Synthetic strategy applied to PEG-dendritic block copolymers.

PEG-dendritic block copolymers can be prepared following three main synthetic strategies^6^: the “direct coupling” of two preformed blocks,^8^ a “chain first” approach where the dendritic block is divergently grown from a PEG at the dendritic focal point,^9^ and a “dendron-first” using a dendritic macroinitiator for the polymerization of ethylene oxide.^10^ Advantages of the “chain first” strategy, such as the possibility to impart different functionalities at the dendron periphery, broad applicability regardless of the dendron G, and the use of PEG as a soluble polymeric support to facilitate purifications, have popularized this approach. Despite the fact that many dendritic families have been incorporated into PEG-dendritic copolymers in this manner, the rigorous synthetic protocols demanded by the branched dendritic architecture impose hurdles. Long reaction times and purifications incompatible with traditional chromatography translate into slow overall processes and modest yields, especially at high dendritic G. These limitations, not specific to PEG-dendritic copolymers, are in fact the challenges that dendritic polymers have faced over the past 30 years.^11^ To address this issue, our group has recently described an accelerated synthesis of dendrimers based on the click^12^ and green^13^ chemistry pillars. The strategy, which involves the thermal azide–alkyne cycloaddition (AAC) and azide substitution reactions, has revealed to be one of the most accelerated, flexible, and user-friendly ever described for the synthesis of dendrimers (Figure 1).^14^

Despite the metal-free AAC is an archetypal click reaction that proceeds with complete atom-economy,^15^ high temperatures and prolonged reaction times have traditionally undermined its application in the complex scenario of the synthesis of dendrimers.^16,17^ Only a combination of activated acetylenedicarboxylate (ADC) repeating units, optimized reaction/purification conditions, and a strict alignment to green chemistry principles (atom-economy and waste reduction) has unveiled the benefits of this chemistry for the accelerated synthesis of dendrimers (Figure 1).^14^ Interestingly, the newly formed triazol branching point in these dendrimers emerges as a key structural element to explore the dendritic chemical space and the adjustment of dendritic properties, such as solubility, rigidity, and biodegradability. This possibility is strengthened by a nearly unlimited access to ADC repeating units via Fischer esterification from commercially available acetylene dicarboxylic acid.

**Figure 2:**
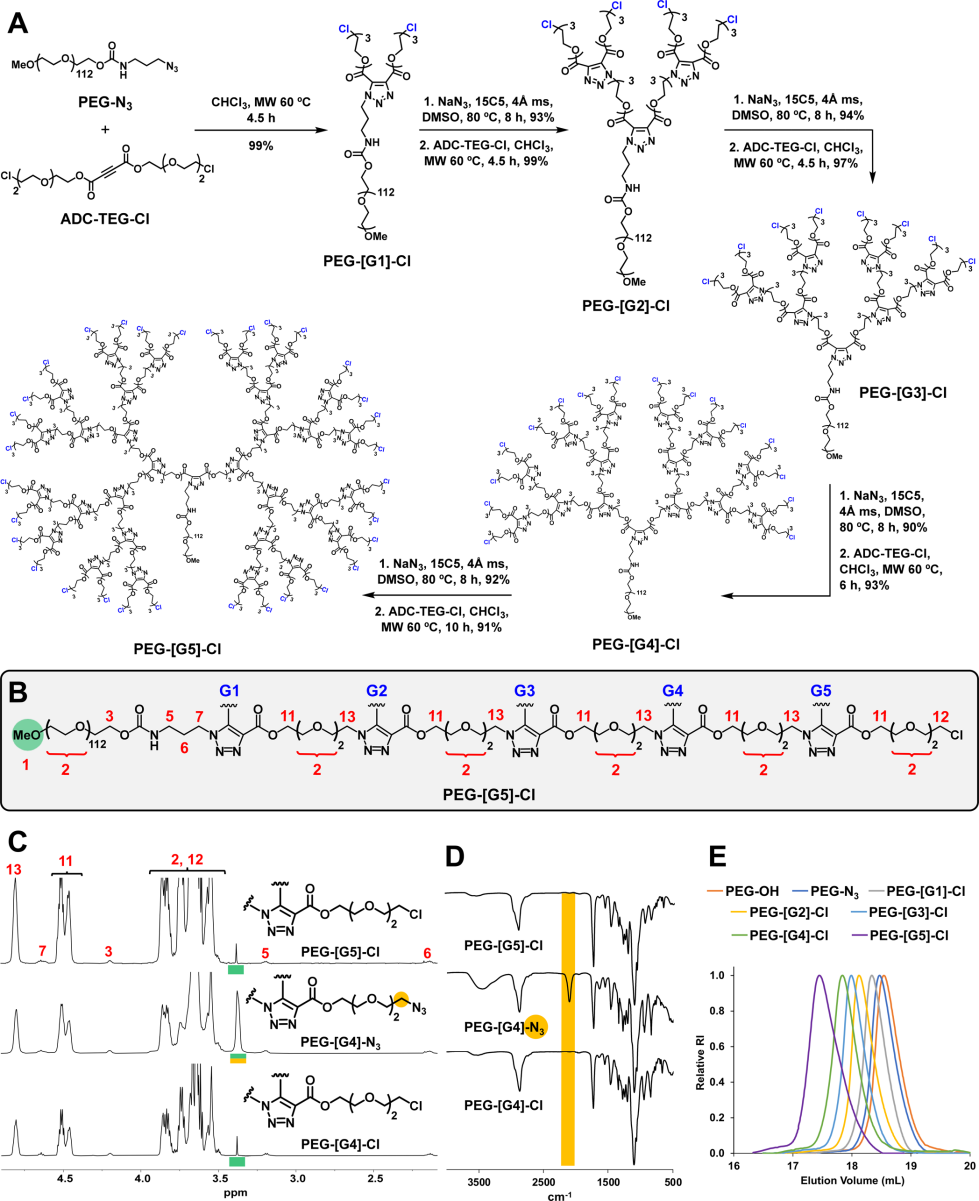
Synthesis of five generations of PEG-dendritic block copolymers via AAC and azide substitution (A). Monitoring of the reaction progress by ^1^H NMR (500 MHz, CDCl_3_) (B, C) and IR (D). GPC elugrams of the copolymers (E).

Herein, we describe our efforts in using the AAC/azide substitution tandem to overcome current limitations in the synthesis and functionalization of PEG-dendritic block copolymers (Figure 1). To this end, a methoxy-terminated PEG incorporating an azide group (PEG-N_3_) and the repeating unit ADC-TEG-Cl (with a hydrophilic triethylene glycol spacer, TEG) were selected as the test bed for the synthesis of PEG-dendritic block copolymers up to G5 (Figure 2). The advantage of the great orthogonality of this sequence was taken for the incorporation of additional functionality in the ADC repeating units, including terminal alkenes and alkynes for the subsequent functionalization of the copolymers by alternative click thiol–ene^18^ and thiol–yne^19^ reactions. In this way, a collection of copolymers with tunable solubility and charge was obtained that was assessed in the preparation of various types of nanoassemblies such as micelles, polymersomes, and nanorods that illustrate the potential of this family of copolymers for drug delivery applications.

## Materials and Methods

### Materials

1-Thio-β-d-galactose sodium salt was purchased from GLYCON Biochemicals and protonated with Dowex 50WX8 before use. Doxorubicin hydrochloride was purchased from Apollo Scientific. LysoTracker Green DND-26 was purchased from Invitrogen Thermo Fisher and bis(benzimide) H-33258 (Hoechst 33258) from Sigma-Aldrich. Calcium (1000 mg/L in water) standard for ion chromatography was purchased from VWR. Phosphorus pentoxide (P_2_O_5_) was purchased from Fluorochem. Bis-dPEG_11_-DBCO was purchased from Quanta Biodesign Limited. Poly-l-arginine hydrochloride (Poly-Arg) with DP 144 by MALLS was purchased from Sigma-Aldrich. All other chemicals were purchased from Sigma-Aldrich or Acros Organics and were used without further purification. All solvents were HPLC grade, purchased from Scharlab, Sigma-Aldrich, or Fisher Scientific and used without further purification. DMSO, CHCl_3_, and pyridine were dried under 4 Å molecular sieves. MeCN, DMF, and CH_2_Cl_2_ were dried using a SPS800 solvent purification system from MBRAUN. NaN_3_ and 15-crown-5 ether (15C5) were dried under a vacuum at 60 °C for 12 h in the presence of P_2_O_5_. H_2_O was of Milli-Q grade, obtained using a Millipore water purification system. Thin-layer chromatography (TLC) was done on silica (60F_254_ from Merck) aluminum-backed plates. PEG-NHS,^20^ 3-azidopropan-1-amine,^21^ di(2-(2-(2-chloroethoxy)ethoxy)ethyl) acetylenedicarboxylate (ADC-TEG-Cl),^14^ di(4-phenylbutyl) acetylenedicarboxylate (ADC-Ar),^14^ di(3-bromopropyl) acetylenedicarboxylate (ADC-Br),^14^ didodecyl acetylenedicarboxylate (ADC-Dod),^22^ diallyl acetylenedicarboxylate (ADC-ene),^23^ and dipropargyl acetylenedicarboxylate (ADC-yne)^23^ were prepared following known procedures.

### Microwave Synthesis

Microwave-assisted reactions were performed in a Discover SP microwave synthesizer (CEM, USA), using a fixed power method with simultaneous external cooling with compressed air. The reaction temperature was controlled with the infrared (IR) sensor integrated in the apparatus that had previously been calibrated with an internal probe provided with a fiber-optic sensor (Thermowell Kit-541165, CEM, USA).^24^ CHCl_3_ used as the solvent in microwave reactions was previously filtered through basic alumina.

### Ultrafiltration

Purifications by ultrafiltration were performed on Millipore Amicon stirred cells with Amicon YM3 regenerated cellulose membranes under 5 psi of N_2_ pressure.

### NMR Spectroscopy

NMR spectra were recorded on a Varian Mercury 300 MHz, Bruker DRX 500 MHz, or Varian Inova 400 MHz spectrometers. Chemical shifts (δ) are reported in ppm relative to the residual solvent peak (7.26 ppm for CDCl_3_, 3.31 ppm for CD_3_OD, 4.79 ppm for D_2_O). ^1^H-diffusion filter experiments were done using a Stimulated-Echo-LED pulse sequence with bipolar PFG gradients. A relaxation delay (d1) was set to 1.5 s and diffusion delays (Δ) to 50, 90, or 100 ms. MestReNova 14.2 software (Mestrelab Research) was used for spectral processing.

### Infrared Spectroscopy

FT-IR spectra were recorded on a PerkinElmer Spectrum Two instrument equipped with a UATR accessory.

### Fluorescence Spectroscopy

Fluorescence spectra were recorded on a Fluorometer FS5 from Edinburgh Instruments (slit widths: 3 nm for excitation and 3 nm for emission). All measurements were taken at room temperature.

### Mass Spectrometry

MALDI-TOF spectra were recorded on a 4800 MALDI-TOF/TOF spectrometer (Applied Biosystems, Foster City, CA, USA). 2,5-Dihydroxybenzoic acid (DHB) was used as the matrix. The block copolymer samples were dissolved in MeOH and 1 μL of the solution was mixed with 20–30 μL of a DHB solution (10 mg/mL in MeOH). The MS spectra were acquired in linear ion mode, with an average of 100 laser shots of wavelength 355 nm. The mass of the copolymers was determined by reference to a Peptide Standard I (Bruker-Daltonics) composed of insulin (*m*/*z* 5734.51), ubiquitin I (*m*/*z* 8565.76), cytochrome C (*m*/*z* 12360.97, 6180.99), and myoglobin (*m*/*z* 16952.30, 8476.65) as internal standards.

### Dynamic Light Scattering (DLS)

DLS measurements were performed on a Malvern Nano ZS (Malvern Instruments, U.K.) operating at 633 nm with a 173° scattering angle at 25 or 37 °C. Mean diameters were obtained from the volume (particles smaller than 100 nm) or intensity (particles larger than 100 nm) particle size distributions provided by Malvern Zetasizer Software. DLS histograms were obtained from the volume or intensity particle size distributions of five independent measurements. Filtering of samples before DLS measurements was avoided to prevent the removal of larger assemblies or aggregates that could obscure the analysis.

### Cryo-Transmission Electron Microscopy (cryo-TEM)

For 2D cryo-imaging, 4 μL of sample (0.5 mg/mL) was applied directly onto a glow-discharged 200-mesh Quantifoil R 2/2 holey-carbon grid and rapidly plunged into liquid ethane with the help of a Vitrobot Mark III (FEI Inc., Eindhoven, The Netherlands). Sample analysis at liquid nitrogen temperature was carried out with a JEM-2200 FS/CR (JEOL Ltd.) transmission electron microscope, using an acceleration voltage of 200 kV and defocus ranging from −1.5 to −5.0 μm. Images were taken under low-dose conditions on a 4K × 4K UltraScan 4000 CCD camera (Gatan Inc., Pleasanton, CA, USA). An in-column Omega energy filter was used in the microscope with the energy slit width set at 30 eV, to improve the signal-to-noise ratio of the images. Average sizes and dimensions were determined with ImageJ software (version 1.51j8) by measuring the line intensity profile of a representative number of assemblies.

### Scanning Electron Microscopy (SEM)

SEM analysis was performed on a FEMSEM-Ultra plus (ZEISS, Germany) with an accelerating voltage of 3 kV and using SE/InLens (secondary electron detector) and/or EsB/AsB (backscattered electron detector). The liquid sample was deposited on a silicon wafer adhered to a SEM stub by using a carbon adhesive disk.

### Gel Permeation Chromatography (GPC)

GPC experiments were performed on an Agilent 1100 series separation module using a PSS SDV precolumn (5 μm, 8 × 50 mm) and two PSS SDV Linear S columns (3 μm, 8 × 300 mm) connected to an Agilent G1362A refractive index detector. THF was used as the eluent at 0.6 mL/min. Samples at 1–1.2 mg/mL were filtered through 0.45 μm PTFE filters before injection.

## Results and Discussion

### AAC/Azide Substitution for the Synthesis of PEG-Dendritic Block Copolymers

For the synthesis of the copolymers, a PEG-N_3_ was selected as a focal point for the divergent growth of the dendritic block (Figure 2A). It was prepared in 93% yield from a commercial methoxy-terminated PEG–OH of 5000 Da by treatment with *N*,*N*′-disuccinimidyl carbonate followed by 3-azidopropan-1-amine (see the SI). The copolymer of G1 was easily obtained from PEG-N_3_ and ADC-TEG-Cl (2.5 equiv per azide) in CHCl_3_ (0.1 M per azide) by heating at 60 °C for 4.5 h under microwave (MW) irradiation (Figure 2A). After solvent evaporation and precipitation (CH_2_Cl_2_/Et_2_O) to remove the excess repeating unit, PEG-[G1]-Cl with two terminal chloride groups was obtained in 99% yield. Subsequent azide substitution (6 equiv of NaN_3_ per Cl) was performed in DMSO (0.1 M per Cl) in the presence of catalytic 15-crown-5 (15C5, 0.1 equiv per Cl) at 80 °C for 8 h. Addition of 4 Å ms in this step was crucial to prevent ester hydrolysis. This side reaction, not observed with non-PEGylated dendrimers,^14^ is related to the hygroscopic nature of the PEG chain. After aqueous workup to remove the excess NaN_3_ and NaCl (the only species generated in the whole reaction sequence) and precipitation (MeOH/*i*PrOH), PEG-[G1]-N_3_ was obtained in 93% yield (Figure 2A). Implementation of this iterative AAC/azide substitution sequence to PEG-[G1]-N_3_ allowed growth of the block copolymer up to G5 with yields higher than 90% per step: G2 (4 terminal groups), G3 (8), G4 (16), and G5 (32) (Figure 2 and SI).

The completion of both steps was easily monitored by ^1^H NMR (characteristic signals of the methylene protons in *alpha* to the azide at 3.30–3.45 ppm, which overlap with the terminal methoxy group of PEG; Figure 2B,C) and IR (intense signal of azide at ca. 2100 cm^–1^, Figure 2D). The growth of the dendritic block was also evident by ^1^H NMR by the increased relative intensity of the methylene protons in *alpha* to the esters (H11) and triazols (H13), as well as the ethylene glycol protons from the TEG spacers (H2), after each sequential cycloaddition step (Figure S1). In addition, MALDI-TOF MS showed molecular weights in very good agreement with calculated values, confirming the efficiency of the iterative synthetic process. The monodispersity of the copolymers was confirmed by gel permeation chromatography (GPC) that showed the expected shift to shorter elution times from PEG–OH and to increasing G from G1 to G5 (Figure 2E). The efficiency of the reactions and the absence of byproducts allowed us to minimize the purification of every new dendrimer G after AAC to just a precipitation (CH_2_Cl_2_/Et_2_O), ensuring the complete recovery of the excess of ADC repeating unit. Azide substitutions were simply followed by an aqueous workup and precipitation (MeOH/*i*PrOH).

### AAC for the Peripheral Functionalization of PEG-Dendritic Block Copolymers

Having confirmed the robustness and fidelity of AAC for the synthesis of PEG-dendritic block copolymers, its application for a subsequent peripheral decoration of the dendritic block was explored. As opposed to classical dendritic families, the advantage of the triazol branching point in AAC-based dendrimers can be taken to explore the dendritic chemical space. By tuning the substituents at the internal alkyne of the ADC units, a vast number of useful building blocks can be envisioned for the peripheral decoration of the dendritic block or its later functionalization by alternative click reactions.

**Figure 3:**
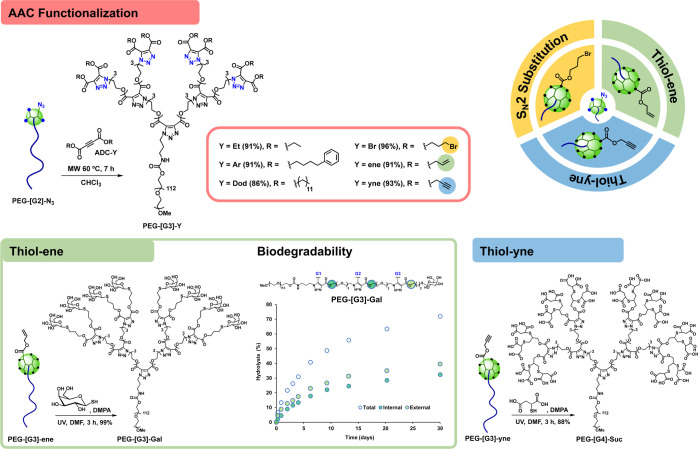
Peripheral functionalization of PEG-dendritic block copolymers by AAC, thiol–ene, and thiol–yne. Hydrolysis of AAC-based PEG-dendritic block copolymers was performed under physiological pH conditions (10 mM PB, pH 7.4, 37 °C).

To this end, the AAC coupling between PEG-[G2]-N_3_ and six ADC units (prepared in gram quantities by Fischer esterification from acetylene dicarboxylic acid and commercially available alcohols) was realized (Figure 3). On the one hand, ADC-Et, ADC-Ar, and ADC-Dod were designed to tune the hydrophilic–hydrophobic balance of the block copolymers and, hence, their ability to self-assemble into nanometric micelles for drug delivery applications. On the other hand, ADC-Br, ADC-ene, and ADC-yne were conceived to render heterolayered dendritic blocks amenable for further functionalization via S_N_2, thiol–ene, or thiol–yne reactions. Complete AAC conversion was reached in all cases (7 h, 60 °C MW, CHCl_3_), affording the expected functionalized copolymers in 86–96% yield (Figure 3) after simple purification by precipitation (MeOH/*i*PrOH). Interestingly, the presence of additional terminal alkenes and alkynes in ADC-ene and ADC-yne repeating units does not compromise the efficiency of the AAC. Complete selectivity for the more activated, internal alkyne was observed with both double-functionalized ADC, rendering PEG-[G3]-ene and PEG-[G3]-yne ready for a subsequent functionalization by orthogonal thiol–ene and thiol–yne reactions.

### Thiol–Ene and Thiol–Yne for the Functionalization of AAC-Based PEG-Dendritic Block Copolymers

Thiol–ene and thiol–yne represent two of the most utilized click reactions in materials science.^25^ Both exhibit high efficiency and yields, fast kinetics, regioselectivity, and broad functional group compatibility. Since these properties match the AAC philosophy, both chemistries were adopted to complement the strategy described herein, providing an additional repertoire for the functionalization of PEG-dendritic block copolymers. Two synthetic objectives were pursued, the preparation of glycodendrimers and dendritic polyelectrolytes (Figure 3).

Carbohydrate–lectin interactions represent the archetypal illustration of multivalency in nature, mediating major biological processes like cell–cell communication, fertilization, or pathogen infection, to mention a few.^26^ In this context, glycodendrimers with precise multivalency attract much attention for triggering or inhibiting natural processes^27^ and as nanotools to unravel the mechanisms controlling these complex interactions.^28^ As for dendritic polyelectrolytes, they have emerged as attractive building blocks for the preparation of polyion complexes (PIC) for drug delivery applications,^29^ where the rigidity of the dendritic scaffold translates into an enhanced stability toward ionic strength.^30^

Accordingly, the functionalization of PEG-[G3]-ene and PEG-[G3]-yne was respectively assayed with 1-thio-β-d-galactose and 2-mercaptosuccinic acid as model ligands toward the preparation of glycodendrimers and dendritic polyelectrolytes (Figure 3).^31,32^ Glycosylation of PEG-[G3]-ene with 1-thio-β-d-galactose (3 equiv per alkene) was performed in DMF in the presence of trace amounts of the photoinitiator 2,2-dimethoxy-2-phenylacetophenone (DMPA) by irradiation with UV (350 nm) for 3 h (Figure 3). After a simple purification by ultrafiltration, the glycodendrimer PEG-[G3]-Gal was obtained in 99% yield. ^1^H NMR analysis of the glycodendrimer confirmed complete disappearance of the characteristic signals of the terminal alkenes between 5.0 and 6.25 ppm. The same reaction conditions applied to PEG-[G3]-yne and 2-mercaptosuccinic acid (6 equiv per alkyne) afforded the polyanion PEG-[G4]-Suc in 88% yield after purification by precipitation (MeOH/*i*PrOH) (Figure 3). Monitoring of the reaction progress was also possible by ^1^H NMR by following the disappearance of the methylene protons at the propargyl groups at around 5.0 ppm. Of note, the branching point generated during the thiol–yne reaction doubles the multivalency of the block copolymer, opening the possibility for an orthogonal AAC/thiol–yne divergent growth.

### Degradability of AAC-Based PEG-Dendritic Block Copolymers

One of the main concerns about polymeric materials is their nondegradability under physiological conditions, a limitation that many dendritic families do not escape. As a result, biodegradable dendrimers attract great interest to prevent bioaccumulation and cytotoxicity.^33^ Carboxylic esters, such as those present in AAC-based dendrimers, are among a reduced number of linkages with a good compromise between synthetic manipulation and biodegradability *in vivo*.^34^ The degradability of AAC-based PEG-dendritic block copolymers was evaluated by ^1^H NMR monitoring the hydrolysis rate of the ester bonds of PEG-[G3]-Gal at physiological pH conditions (10 mM phosphate buffer PB, pH 7.4, 37 °C; see the SI). As shown in Figure 3, the hydrolysis was time-dependent, with more than 40% of the ester bonds hydrolyzed after 1 week, which suggests that these copolymers can be degraded into small pieces *in vivo* and thus easily excreted from the body, thereby avoiding systemic toxicity. No significant differences were observed in the rate of hydrolysis of the esters in different layers of the dendritic block.

### Assemblies of AAC-Based PEG-Dendritic Block Copolymers for Drug Delivery

In the following three sections, the usefulness of AAC-based PEG-dendritic block copolymers for the preparation of polymeric assemblies with potential applications in drug delivery will be discussed. As mentioned above, block copolymers are interesting materials with the ability to assemble in solution due to differences in the solubility or charge properties of the blocks.^1−6^ The resulting assemblies can encapsulate drugs and protect them from the surrounding media after *in vivo* administration, and eventually, deliver them to target cells and organs either by passive or active targeting, which makes them useful in cancer medicine, gene therapy, and diagnostics.^35,36^

**Figure 4:**
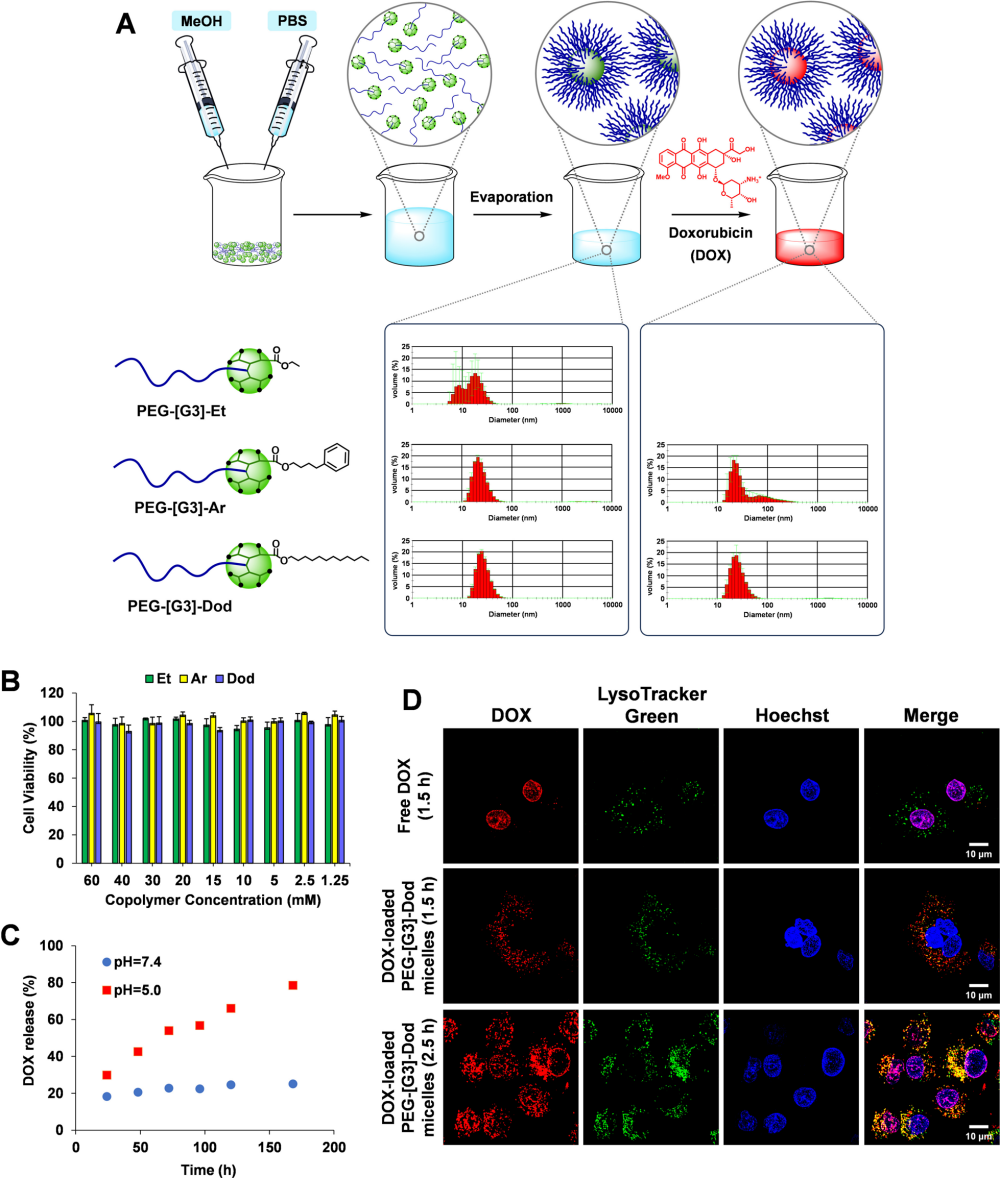
Influence of the hydrophilic–hydrophobic balance of the blocks on the ability of AAC-based PEG-dendritic block copolymers to self-assemble into nanometric micelles and encapsulate DOX. DLS histograms of blank and DOX-loaded micelles in PBS (8 mg of block copolymer/mL) (A). Cell viability (CCK-8 assay) of PEG-[G3]-Et, PEG-[G3]-Ar, and PEG-[G3]-Dod micelles after 48 h in A549 cells (B). pH-dependent release of DOX from PEG-[G3]-Dod micelles (C). Intracellular trafficking of DOX-loaded PEG-[G3]-Dod micelles in A549 cells was assessed by confocal microscopy (D).

**Figure 5:**
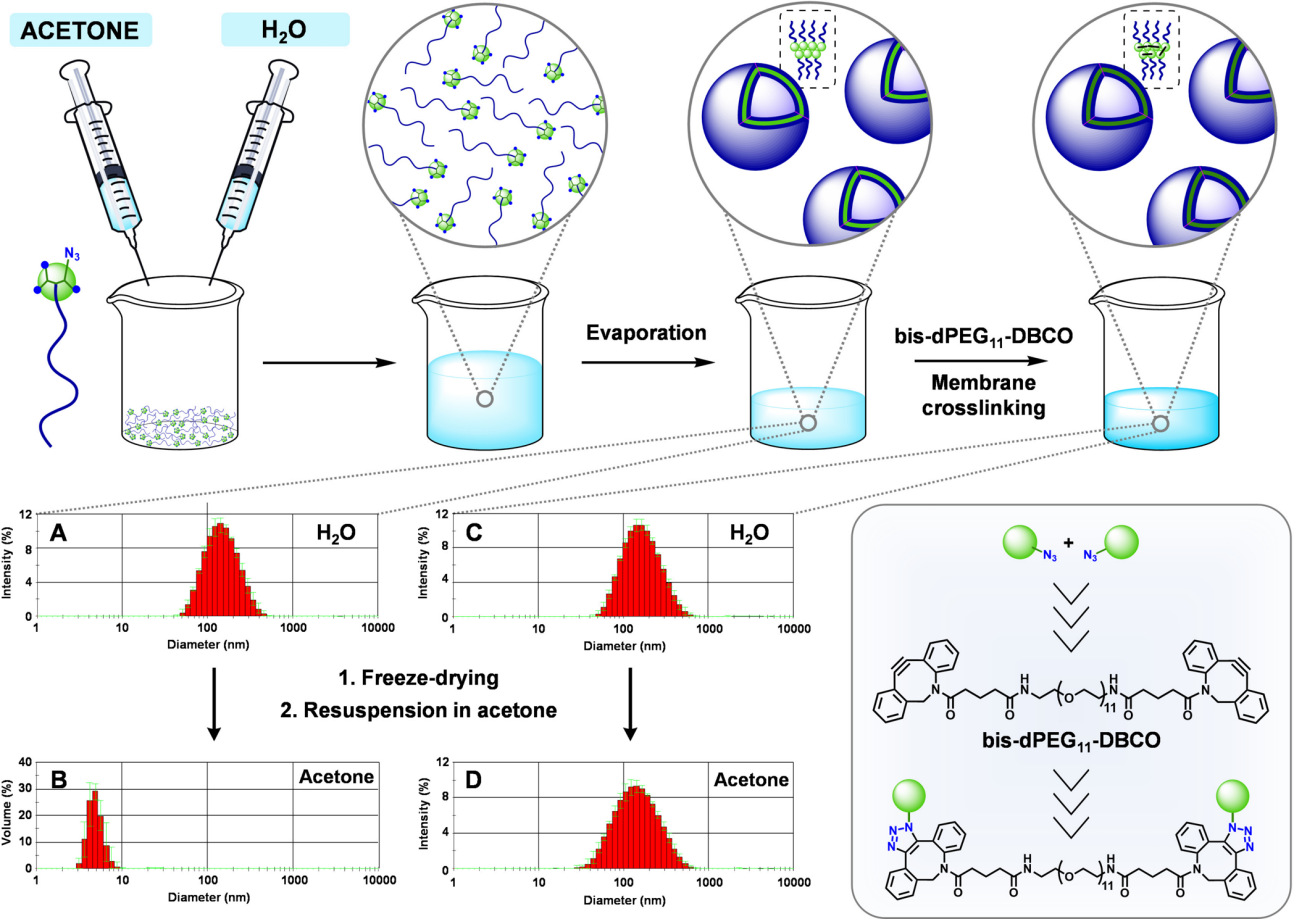
Preparation of polymersomes from PEG-[G2]-N_3_ and subsequent cross-linking of the membrane by SPAAC using a dicyclooctyne cross-linker. DLS histograms in H_2_O (A and C) and acetone (B and D) before (A and B) and after (C and D) cross-linking (4 mg/mL).

**Figure 6:**
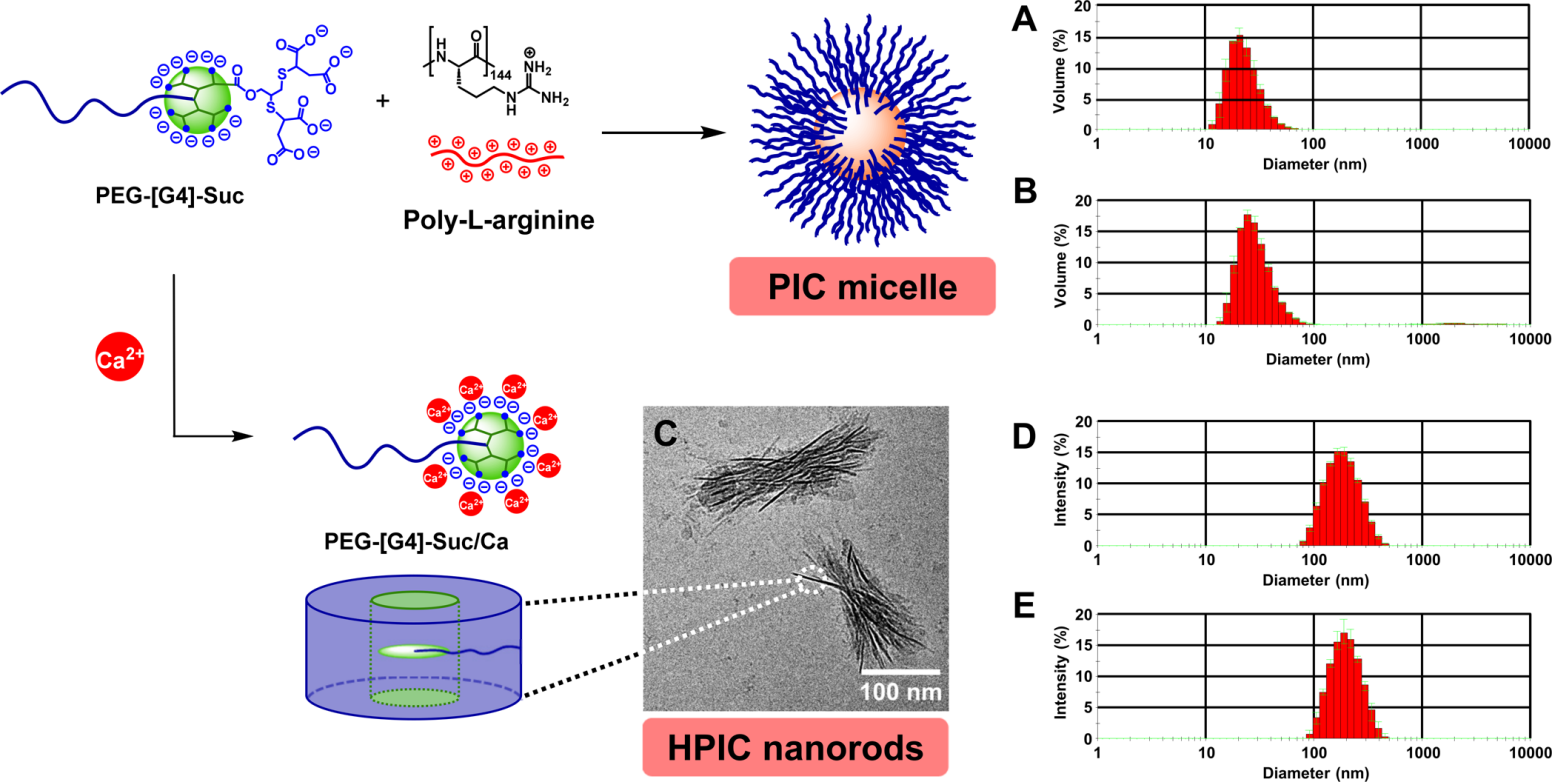
PIC micelles prepared from PEG-[G4]-Suc and Poly-Arg. DLS histograms upon formation (A) and after 24 h at 37 °C in the presence of 150 mM NaCl (B). HPIC nanorods were prepared from PEG-[G4]-Suc and calcium. Cryo-TEM image (C) and DLS histograms before (D) and after cross-linking (1,2-ethylenediamine, EDC) (E). While noncross-linked HPIC disintegrate after addition of EDTA, they remained stable if cross-linked (see the SI).

With this aim, three AAC-based block copolymers of Figure 3, namely, PEG-[G3]-Et (C_2_ aliphatic ester), PEG-[G3]-Ar (C_10_ aromatic ester), and PEG-[G3]-Dod (C_12_ aliphatic ester) were selected to assess the influence of the hydrophilic–hydrophobic balance of the blocks on their ability to self-assemble into nanometric micelles for the encapsulation of the anticancer drug doxorubicin (DOX) (Figure 4). A second section showcases how the presence of terminal azides in AAC-based PEG-dendritic block copolymers can be exploited in the preparation of cross-linked polymersomes for improved stability (Figure 5). Finally, the last section describes the preparation of polyion complex (PIC) micelles from polyanionic PEG-[G4]-Suc with oppositely charged cationic linear polymers and divalent cations (Figure 6).

### Amphiphilic Block Copolymer Micelles for the Delivery of Doxorubicin

Micelles from PEG-[G3]-Et, PEG-[G3]-Ar, and PEG-[G3]-Dod were prepared by following an evaporation method (Figure 4A). The copolymers were dissolved in MeOH and then, 10 mM PB pH 7.4, 150 mM NaCl was slowly added while vortexing (final volume ratio, 1:1). After evaporation of MeOH, the formation of micelles was examined by DLS. PEG-[G3]-Et, the copolymer with the smallest hydrophobic block, did not afford discrete micelles. A dual size distribution was observed by DLS, showing species smaller than 10 nm attributed to the free copolymer in solution. Conversely, PEG-[G3]-Ar and PEG-[G3]-Dod with larger hydrophobic blocks led to highly monodisperse micelles with a mean diameter of 25 nm, which matches that of a theoretical micelle with fully extended block copolymer chains. Interestingly, DLS studies on the effect of G on the self-assembly of the copolymers pointed to G3 as the preferred G, with G2 and G4 for the Ar-derivative also leading to second populations of larger assemblies (Figure S3).

The potential application of these micelles in drug delivery was assessed by analyzing their cytotoxicity profile and the encapsulation and release of doxorubicin (DOX). The cytotoxicity was assessed by CCK-8 assay after 48 h in A549 cells. Even at the maximum concentration analyzed (60 mM of copolymers, equivalent to ca. 0.50 mg/mL, Table S1), no effect on cell viability was observed (Figure 4B). DOX is a frontline chemotherapeutic drug widely used for the treatment of various cancers, lymphomas, and certain leukemias, which unfortunately produces severe side-effects including life-threatening cardiotoxicity, forcing the treatment to become dose-limiting and the search for efficient DDS.^37^ The encapsulation of DOX was achieved by adding an aqueous solution of the drug to freshly prepared PEG-[G3]-Ar and PEG-[G3]-Dod micelles (40 mol % DOX relative to the dendrimer peripheral Ar or Dod groups) followed by dialysis against PBS. As seen in Figure 4A, DOX-loaded PEG-[G3]-Dod micelles were indistinguishable from the blank micelles by DLS, affording an unoptimized encapsulation efficiency (EE) of 80% and a drug loading (DL) of 19%. Conversely, the same procedure applied to PEG-[G3]-Ar led to micelle aggregation, highlighting large differences in drug encapsulation induced by subtle structural variations between the Ar and Dod groups. In line with this, when the critical micelle concentration (CMC) of both black micelles was determined using pyrene as a fluorescent probe, PEG-[G3]-Dod afforded a smaller value (12 vs 42 μg/mL, see the SI). As a result of this screening, PEG-[G3]-Dod, the copolymer with the largest hydrophobic block, emerged as a promising candidate for evaluation in DD.

Since cell internalization of polymeric micelles typically occurs by endocytosis, the *in vitro* release profile of DOX from PEG-[G3]-Dod micelles was analyzed at two different pH values: 5.0 representative of acidic endosome/lysosome organelles, and 7.4 for the extracellular milieu. As shown in Figure 4C, almost 80% of the drug was released in a sustained way within 1 week at pH 5.0, while only a modest burst release was observed at pH 7.4. This selective release of DOX under acidic conditions encouraged us to perform a cell internalization study to confirm the pH-triggered release of the drug after cell internalization by endocytosis. Thus, DOX-loaded PEG-[G3]-Dod micelles were incubated with A549 cells, and the intracellular trafficking of DOX was monitored by confocal microscopy and compared to that of the free drug. As seen in Figure 4D, after just 1.5 h of incubation, free DOX (red) was exclusively detected in cell nuclei as determined by complete colocalization with Hoechst (blue). Because of its hydrophobic character, free DOX can cross cell membranes by diffusion and quickly migrate to the cell nuclei. Conversely, micellar DOX exclusively colocalized at the same time with LysoTracker Green (green), a well-known endosome/lysosome marker. It was only after 1 h of additional incubation time that, in response to the acidic environment of the endosome/lysosome, localization of DOX was observed in both the endosome/lysosome and nucleus. This is interpreted as the result of a pH-triggered release of the drug followed by endosomal escape and migration to the cell nuclei.

Overall, PEG-[G3]-Dod has been identified as a promising prototype for DD. The small size of these micelles, their stability at physiological pH and ability to encapsulate drugs, along with an expected longer circulation time *in vivo* and improved biodistribution compared to the free drug, are interesting properties that deserve further investigation. The easy access to a large number of ADC repeating units structurally related to ADC-Dod suggests a straightforward optimization via adjustment of the dendritic properties.

### Membrane Cross-Linked Polymersomes

A potential limitation of polymeric micelles and vesicles (polymersomes) prepared by assembly of amphiphilic block copolymers is their dynamic nature, which might lead to instability at the higher temperatures and lower concentrations faced after administration *in vivo*. As a result, there has been significant effort toward their stabilization by selective cross-linking of the blocks at the micelle core or polymersome membrane to prevent premature disintegration.^38,39^

In this context, the advantage of the presence of terminal azides in AAC-based PEG-dendritic block copolymers has been taken for the cross-linked stabilization of polymersomes using the strain-promoted azide–alkyne cycloaddition (SPAAC).^40^ PEG-[G2]-N_3_ was chosen to assess the feasibility of this approach using a dicyclooctyne (bis-dPEG_11_-DBCO) cross-linker (Figure 5).

Polymersomes from PEG-[G2]-N_3_ were obtained with a mean diameter of ca. 160 nm by DLS following an evaporation method (acetone/H_2_O; 1:1) (Figure 5A). Their vesicular structure with a uniform lamella was confirmed by cryo-transmission electron microscopy (cryo-TEM) (Figure S5). When these polymersomes were freeze-dried and resuspended in acetone (a good solvent for the block copolymer), only species smaller than 10 nm were observed by DLS (Figure 5B), attributed to the free block copolymer in solution, confirming the disintegration of the assembly. Conversely, when the polymersomes were treated with bis-dPEG_11_-DBCO for 8 h at rt (0.15 equiv. per azide), the size was retained after freeze-drying and resuspension in acetone (Figure 5D), supporting the stabilization imparted by the SPAAC cross-linking. Of note, the substoichiometric amount of added cross-linker preserves a relatively high number of unreacted azides in the cross-linked polymersomes, amenable for subsequent functionalization with ligands, imaging probes, or prodrugs using any of the azide–alkyne cycloaddition variants.

### Polyion Complexes (PICs) from Block Copolymers

Since the emergence of DDS, polyion complex (PIC) micelles prepared from oppositely charged PEGylated copolymers (or a block copolymer and a polyelectrolyte) at stoichiometric charge ratios have attracted much attention.^41,42^ The presence of the PEG allows confining the electrostatic interaction between the charged blocks within a nanometric core, protected from the surrounding media.^43−45^ This architecture results in neutral micelles with a narrow size distribution, well suited for the delivery of small drugs and biopharmaceuticals.^46^

Nonetheless, the electrostatic interaction driving the formation of PIC micelles also compromises their stability at ionic strengths as low as 150 mM, characteristic of physiological conditions. To overcome this limitation, our group proposed the use of charged dendrimers. The intrinsic rigidity and globular nature of dendrimers^47^ result in PIC assemblies (micelles and vesicles) with unprecedented stability up to ionic strengths higher than 3 M, the highest achieved for PIC.^30,48−51^

The usefulness of AAC-based PEG-dendritic block copolymers in the preparation of PIC micelles was analyzed with PEG-[G4]-Suc (Figure 3), an anionic dendritic copolymer with 32 terminal carboxylates, and poly-l-arginine (Poly-Arg, DP 144) as oppositely charged cationic polymer (Figure 6). Upon mixing solutions of both polyelectrolytes in 10 mM PB at a stoichiometric charge ratio, monodisperse micelles were obtained with a mean diameter of 24 nm by DLS (Figure 6A). This value, which matches the expected diameter for a micelle with fully extended block copolymer chains, remained unaffected, even after heating for 24 h at 37 °C in the presence of 150 mM NaCl (Figure 6B).

Encouraged by this result, we decided to challenge PEG-[G4]-Suc in the formation of hybrid polyion complexes (HPIC), a specific type of PIC prepared by complexation of anionic block copolymers to multivalent metal cations.^52,53^ Upon complexation, the electrostatic neutralization of the polyanion leads to hydrophobization and spontaneous self-assembly into diverse structural morphologies depending on the length of the blocks. HPIC have been developed as powerful contrast agents for MRI,^54^ templates for the preparation of core cross-linked micelles^55^ and metal nanoparticles,^56−58^ in the construction of Li batteries^59^ or as platforms for renewable catalysis.^60^

To test the formation of HPIC, an equimolecular amount of calcium ions (16 equiv) was added to a solution of PEG-[G4]-Suc (16 terminal mercaptosuccinic acids) in 10 mM PB. Nanometric assemblies were observed by DLS with a mean diameter of 190 nm, which remained stable for days (Figure 6D). Analysis of these complexes by cryo-TEM revealed bundles of ca. 150–200 nm of length and 50 nm wide composed of nanorods with average diameter of 4.0 ± 0.5 nm (Figure 6C). This cross-sectional diameter is compatible with a columnar assembly of hydrophobized dendron blocks surrounded by a palisade of PEG chains (not observable because of low contrast by cryo-TEM). The presence of these bundles in the dried state was also confirmed by SEM (Figure S6). Interestingly, the complex formation completely vanished when the HPIC solution was treated with an equimolecular amount of ethylenediaminetetraacetic acid (EDTA), a calcium chelating agent (DLS histogram in Figure S7), confirming the calcium complexation as the driving force in the production of HPIC.

As described by Bronich et al. for HPIC micelles,^55,61^ these calcium HPIC were exploited as templates for the synthesis of core cross-linked nanorods with potential application in cancer therapy by encapsulation of platinum anticancer agents. To this end, HPIC were treated with 1,2-ethylenediamine and EDC to afford amide cross-linking between carboxylic acids. Analysis by DLS revealed little change in size (Figure 6E), suggesting that cross-linking was limited to intrarod reactions. When these cross-linked HPICs were treated with EDTA to remove calcium ions under same conditions as above, no effect on size was observed by DLS (Figure S7), confirming the efficiency of the covalent cross-linking replacing calcium ions. Overall, we have demonstrated the ability of PEG-[G4]-Suc (a copolymer synthesized in gram quantities in just 6 steps from PEG-N_3_ and two readily accessible ADC units) to participate in the preparation of PIC and HPIC with promising applications in DD.

## Conclusions

The AAC/azide substitution tandem is revealed as a flexible, reliable, atom-economical, and user-friendly strategy for the synthesis and functionalization of biodegradable PEG-dendritic block copolymers. Using a PEG-N_3_ with a terminal azide group as focal point for a divergent dendritic growth and the acetylenedicarboxylate ADC-TEG-Cl as repeating unit, a G5 copolymer with 32 terminal groups was readily prepared in gram quantities and excellent yield. The great orthogonality of the sequence was exploited for the incorporation of additional functionality in the ADC repeating units, such as terminal alkenes and alkynes, for the subsequent functionalization of the copolymers by alternative click thiol–ene and thiol–yne reactions. As a result, a collection of AAC-based copolymers with tunable solubility and charge was obtained as a platform for the preparation of various types of nanoassemblies with promising applications in DD: amphiphilic block copolymer micelles, cross-linked polymersomes, and various types of polyion complexes.

## Supporting Information Available

Experimental procedures and characterization (PDF)
