# Supplementary material for: Modular Synthesis of PEG-Dendritic Block Copolymers by Thermal Azide–Alkyne Cycloaddition with Internal Alkynes and Evaluation of their Self-Assembly for Drug Delivery Applications
Source: Biomacromolecules. 2024 Apr 13;25(5):2780–91. doi: 10.1021/acs.biomac.3c01429 (PMC11094729; doi:10.1021/acs.biomac.3c01429)
Supplement: Supplementary file 1 — bm3c01429_si_001.pdf [file bm3c01429_si_001.pdf]

Supporting Information for:

Modular Synthesis of PEG-Dendritic Block Copolymers by Thermal Azide-Alkyne Cycloaddition with Internal Alkynes and Evaluation of their Self-Assembly for Drug Delivery Applications

*Samuel Parcero-Bouzas, Juan Correa, Celia Jimenez-Lopez, Bruno Delgado Gonzalez, and Eduardo Fernandez-Megia**

Centro Singular de Investigación en Química Biolóxica e Materiais Moleculares (CIQUS), Departamento de Química Orgánica, Universidade de Santiago de Compostela, Jenaro de la Fuente s/n, 15782 Santiago de Compostela, Spain.

Table of Contents

1. Synthesis and Characterization of New Compounds S3

2. Degradability of the Dendritic Structure S46

3. Amphiphilic and Doxorubicin (DOX) Loaded Micelles S47

4. Membrane Crosslinked Polymersomes S53

5. PIC and Hybrid-PIC S54

1. Synthesis and Characterization of New Compounds

PEG-N_3. Et_3N (150 µL, 1.08 mmol) was added to a solution of PEG-NHS (1.87 g, 0.359 mmol) and 3-azidopropan-1-amine (180 mg, 1.79 mmol) in CH_2Cl_2 (8 mL). The mixture was stirred at rt for 12 h and then, concentrated and precipitated from CH_2Cl_2/Et_2O (-20 °C) to give PEG-N_3 (1.73 g, 93%) as a white solid. ^1H NMR (500 MHz, $CDCl_3$) δ: 5.02 (bs, 1H), 4.22 (t, J = 4.6 Hz, 2H), 3.86 – 3.43 (m, ~450H), 3.42 – 3.33 (m, 5H), 3.27 (q, J = 6.4 Hz, 2H), 1.79 (quint, J = 6.6 Hz, 2H). ^{13}C NMR (126 MHz, $CDCl_3$) δ: 156.5, 72.0, 71.7, 71.4, 70.6, 69.8, 69.6, 64.0, 59.0, 49.0, 38.4, 29.2. IR (ATR, cm^{-1}): 2866, 2095, 1722, 1094. MALDI-TOF MS (DHB, linear mode): *m/z* Calcd: M_p ($[M+Na]^+$) 5144, M_n 5169; Found: M_p 5113, M_n 5201, M_w 5247.

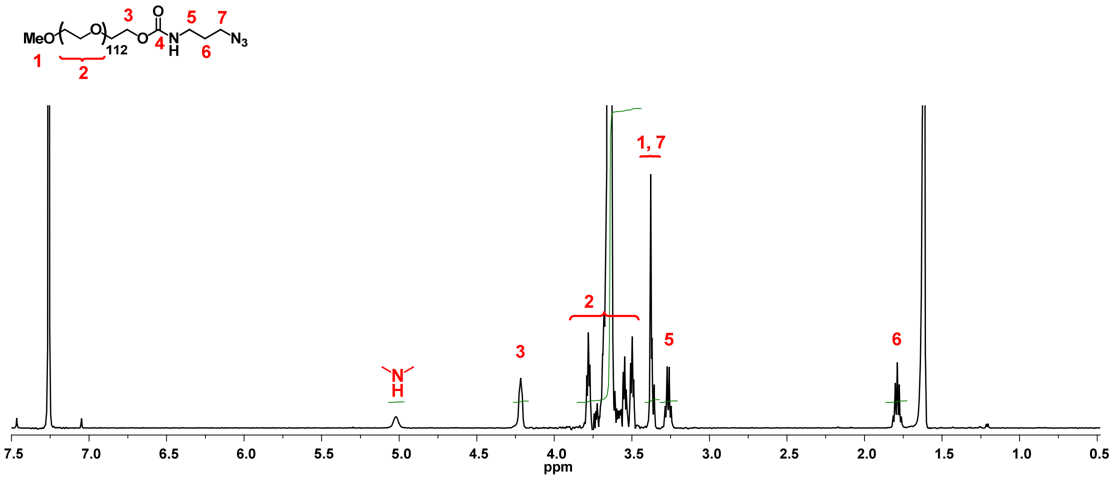

^1H NMR spectrum of PEG-N_3 ($CDCl_3$)

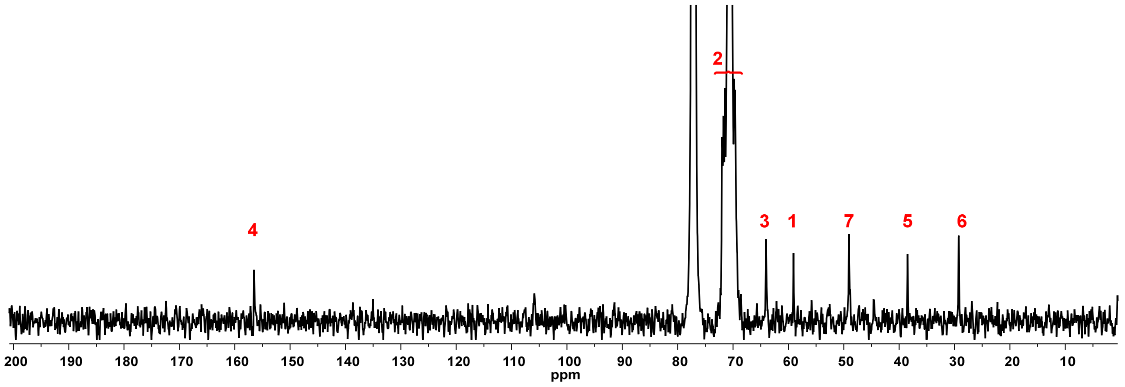

^{13}C NMR spectrum of PEG-N_3 ($CDCl_3$)

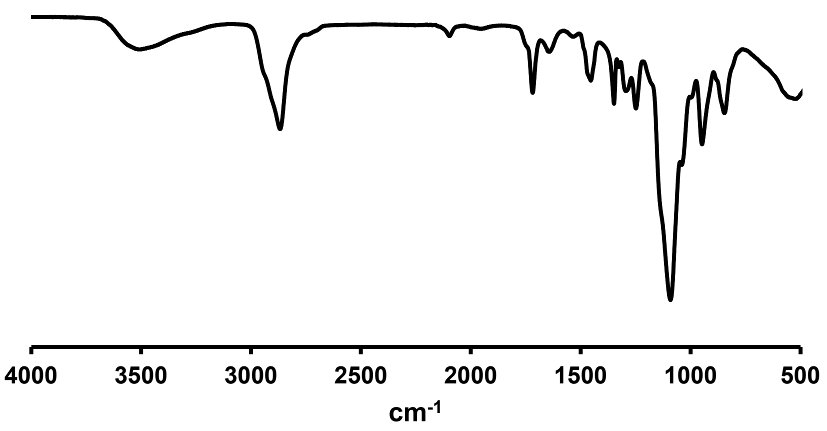

IR spectrum of PEG-N_3

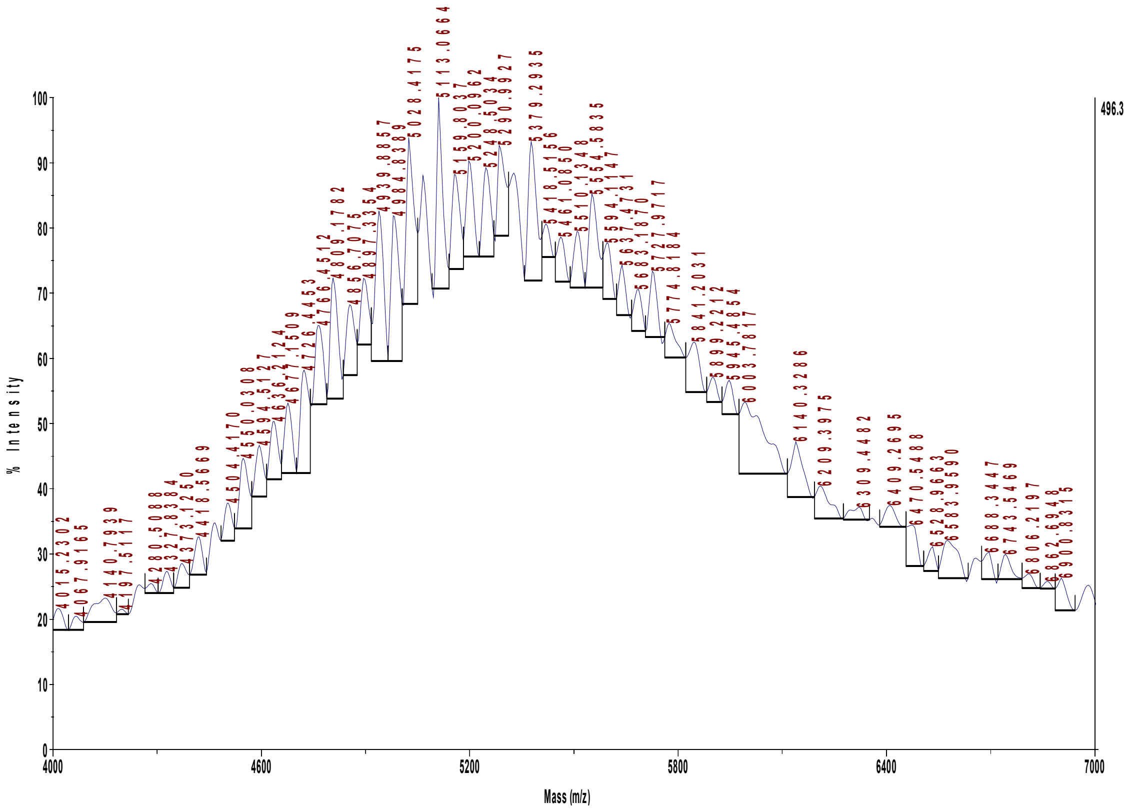

MALDI-TOF spectrum of PEG-N_3

PEG-[G1]-Cl. A solution of PEG-N_3 (500 mg, 0.096 mmol) and ADC-TEG-Cl (100 mg, 0.241 mmol) in $CHCl_3$ (1 mL) was heated under microwave radiation (60 ºC, 20 W, 14 psi airflow) for 4.5 h. Then, the reaction mixture was concentrated and precipitated from CH_2Cl_2/Et_2O (-20 °C) to give PEG-[G1]-Cl (539 mg, 99%) as a white solid. ^1H NMR (500 MHz, $CDCl_3$) δ: 5.38 (bs, 1H), 4.68 (t, J = 6.9 Hz, 2H), 4.63 – 4.57 (m, 2H), 4.57 – 4.51 (m, 2H), 4.25 (t, J = 4.6 Hz, 2H), 3.91 – 3.43 (m, ~470H), 3.40 (s, 3H), 3.22 (q, J = 6.5 Hz, 2H), 2.17 (quint, J = 7.0 Hz, 2H). ^{13}C NMR (101 MHz, $CDCl_3$) δ: 159.9, 158.1, 156.4, 140.0, 130.0, 71.8, 71.2, 70.4, 69.4, 68.7, 68.4, 65.6, 64.7, 63.9, 58.9, 47.9, 42.7, 37.5, 30.2. IR (ATR, cm^{-1}): 2885, 1722, 1105. MALDI-TOF MS (DHB, linear mode): *m/z* Calcd: M_p ($[M+H]^+$) 5537, M_n 5562; Found: M_p 5533, M_n 5570, M_w 5597.

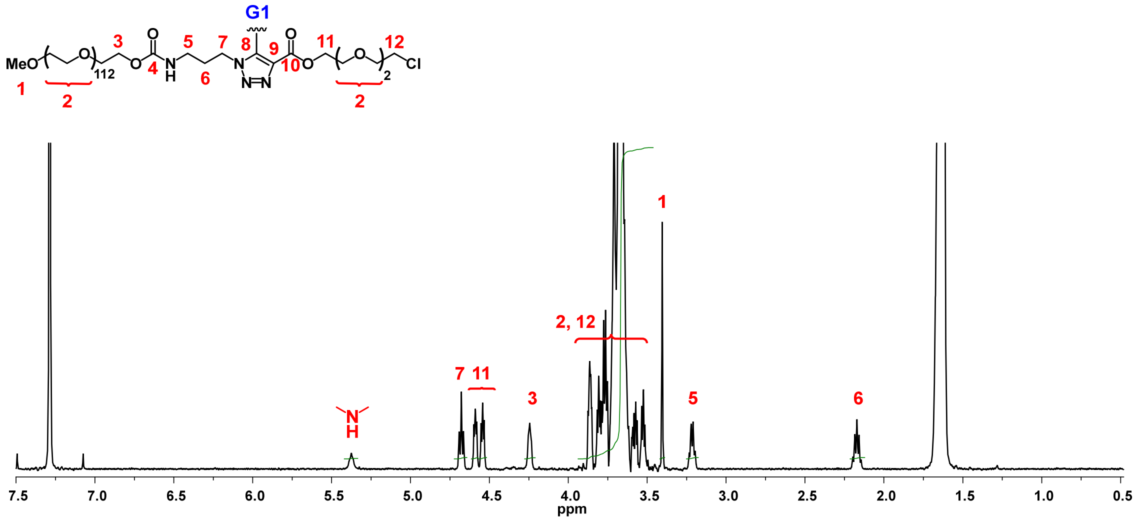

^1H NMR spectrum of PEG-[G1]-Cl ($CDCl_3$)

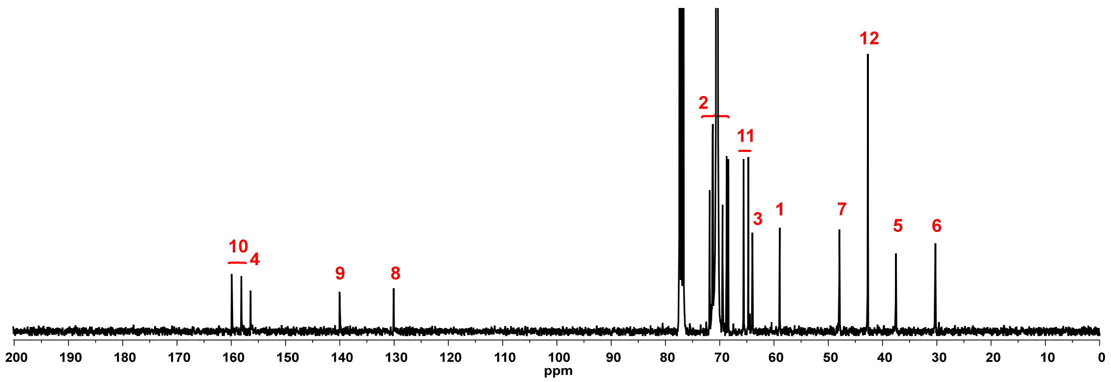

^{13}C NMR spectrum of PEG-[G1]-Cl ($CDCl_3$)

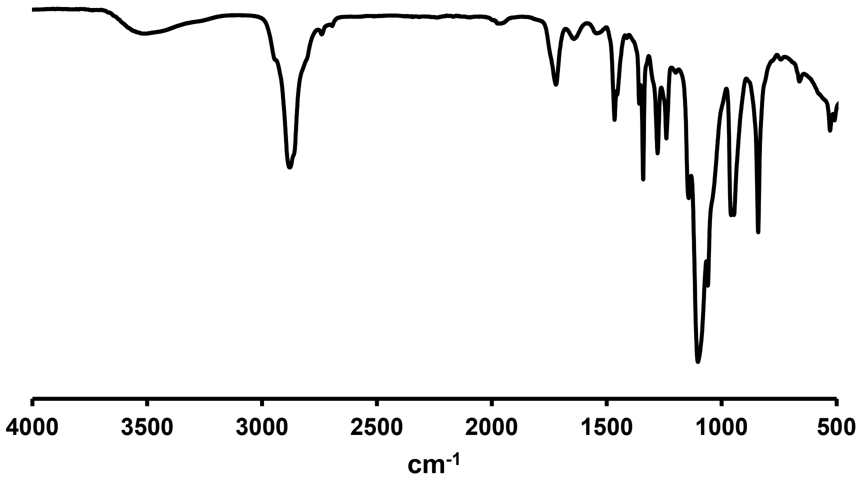

IR spectrum of PEG-[G1]-Cl

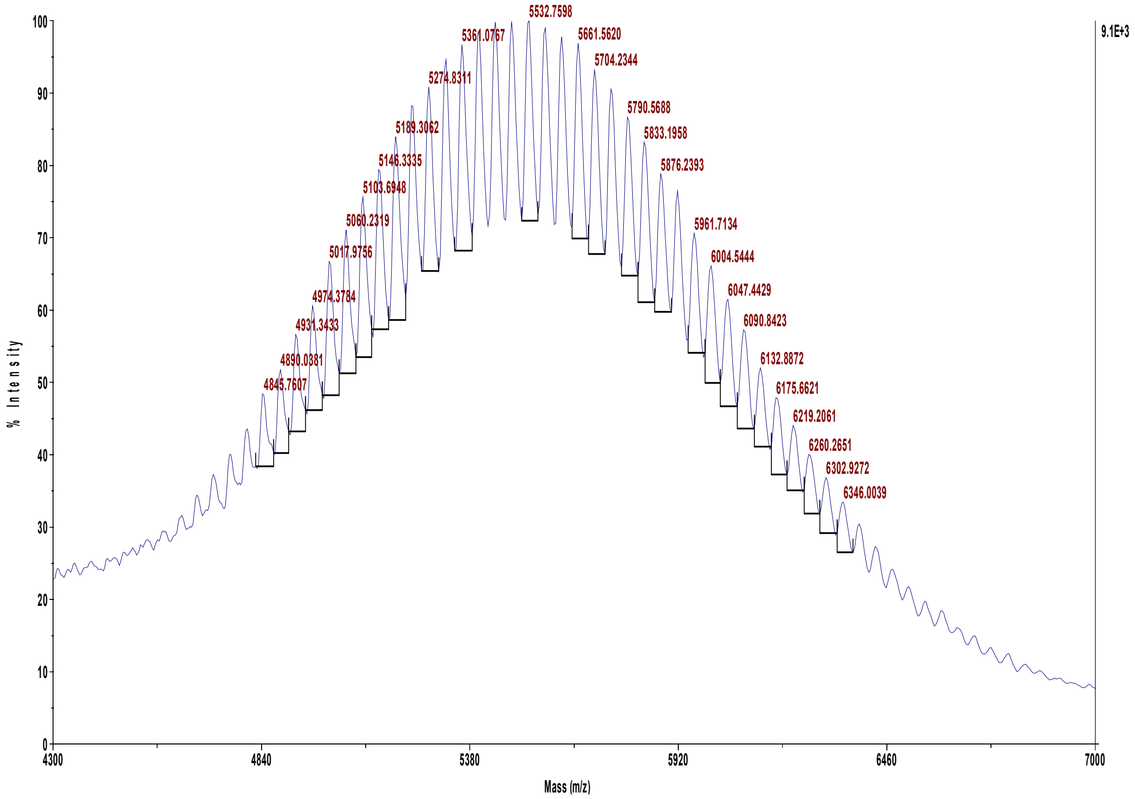

MALDI-TOF spectrum of PEG-[G1]-Cl

PEG-[G1]-N_3. A suspension of PEG-[G1]-Cl (500 mg, 0.089 mmol), NaN_3 (69 mg, 1.07 mmol), 15-crown-5 (3.9 mg, 0.018 mmol), and molecular sieves (4Å, 100 mg) in dry DMSO (1.78 mL) was stirred under Ar at rt for 5 h and then, heated at 80 °C for 8 h. The resulting suspension was diluted with $CHCl_3$ to double the original volume and filtered. The filtrate was diluted with $CHCl_3$ (100 mL), washed with MilliQ H_2O (3 x 100 mL) and brine (1 x 100 mL), dried, and concentrated. The obtained residue was precipitated from MeOH/*i*PrOH (-20 °C) to give PEG-[G1]-N_3 (467 mg, 93%) as a pale-yellow solid. ^1H NMR (500 MHz, $CDCl_3$) δ: 5.30 (bs, 1H), 4.65 (t, *J* = 6.9 Hz, 2H), 4.59 – 4.54 (m, 2H), 4.54 – 4.47 (m, 2H), 4.22 (t, *J* = 4.6 Hz, 2H), 3.91 – 3.43 (m, ~466H), 3.42 – 3.31 (m, 7H), 3.19 (q, *J* = 6.2 Hz, 2H), 2.14 (quint, *J* = 6.7 Hz, 2H). IR (ATR, cm^{-1}): 2885, 2102, 1724, 1105.

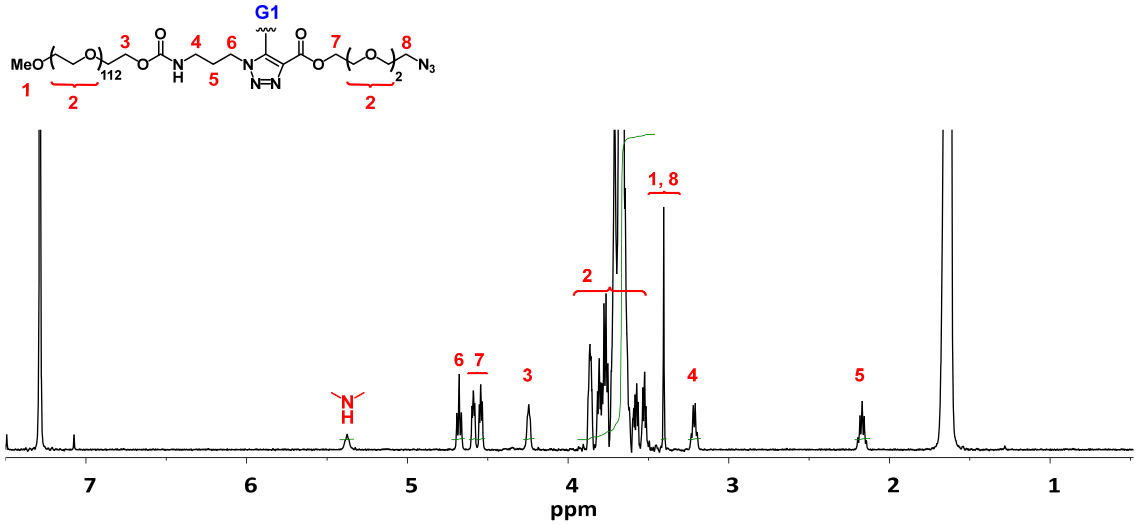

^{1}H NMR spectrum of PEG-[G1]-N_3 ($CDCl_3$)

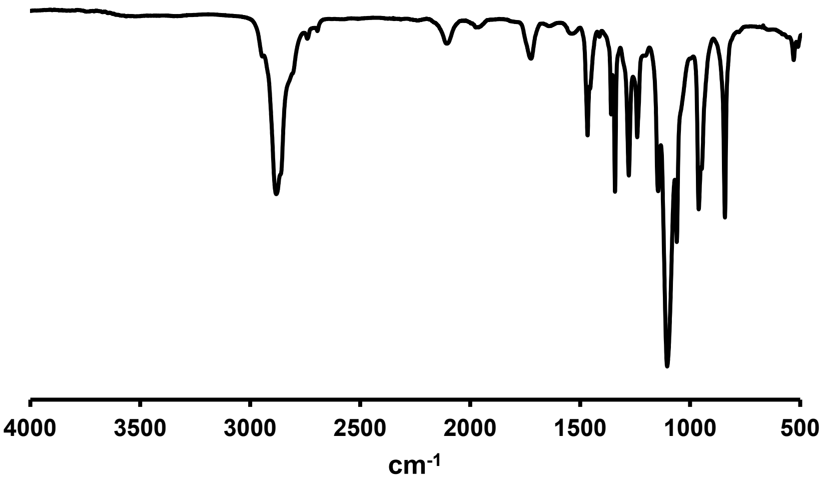

IR spectrum of PEG-[G1]-N_3

PEG-[G2]-Cl. A solution of PEG-[G1]-N_3 (468 mg, 0.083 mmol) and ADC-TEG-Cl (173 mg, 0.42 mmol) in $CHCl_3$ (1.7 mL) was heated under microwave radiation (60 ºC, 20 W, 14 psi airflow) for 4.5 h. Then, the reaction mixture was concentrated and precipitated from CH_2Cl_2/Et_2O (-20 °C) to give PEG-[G2]-Cl (533 mg, 99%) as a white solid. ^{1}H NMR (500 MHz, $CDCl_3$) δ: 5.35 (bs, 1H), 4.86 – 4.76 (m, 4H), 4.65 (t, J = 6.9 Hz, 2H), 4.56 – 4.42 (m, 12H), 4.22 (t, J = 4.6 Hz, 2H), 3.94 – 3.43 (m, ~506H), 3.38 (s, 3H), 3.19 (q, J = 6.3 Hz, 2H), 2.14 (quint, J = 6.7 Hz, 2H). ^{13}C NMR (101 MHz, $CDCl_3$) δ: 159.9, 158.2, 156.5, 140.0, 139.6, 139.5, 131.6, 131.3, 130.2, 71.7, 71.6, 71.0, 70.5, 69.5, 69.4, 69.2, 68.7, 68.6, 68.5, 68.4, 65.6, 65.5, 64.7, 64.6, 63.9, 59.0, 50.2, 50.1, 48.0, 42.8, 37.6, 30.2. IR (ATR, cm^{-1}): 2885, 1732, 1109. MALDI-TOF MS (DHB, linear mode): *m/z* Calcd: M_p ($[M+Na]^+$) 6403, M_n 6428; Found: M_p 6353, M_n 6319, M_w 6378.

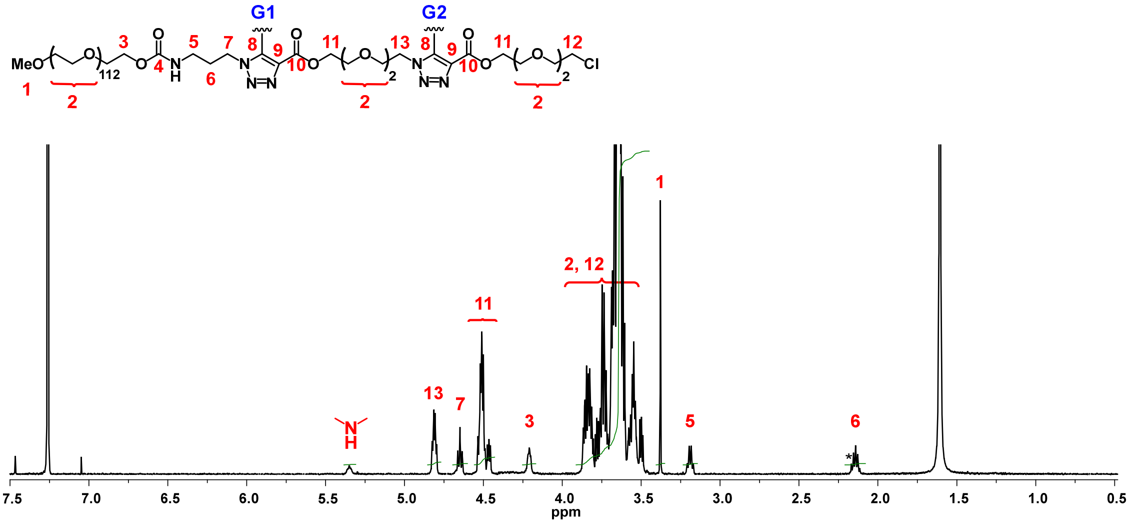

^{1}H NMR spectrum of PEG-[G2]-Cl ($CDCl_3$)

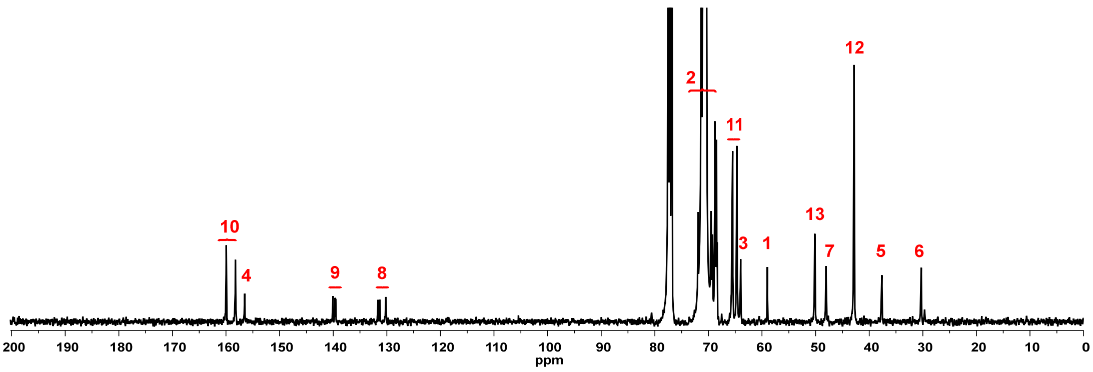

^{13}C NMR spectrum of PEG-[G2]-Cl ($CDCl_3$)

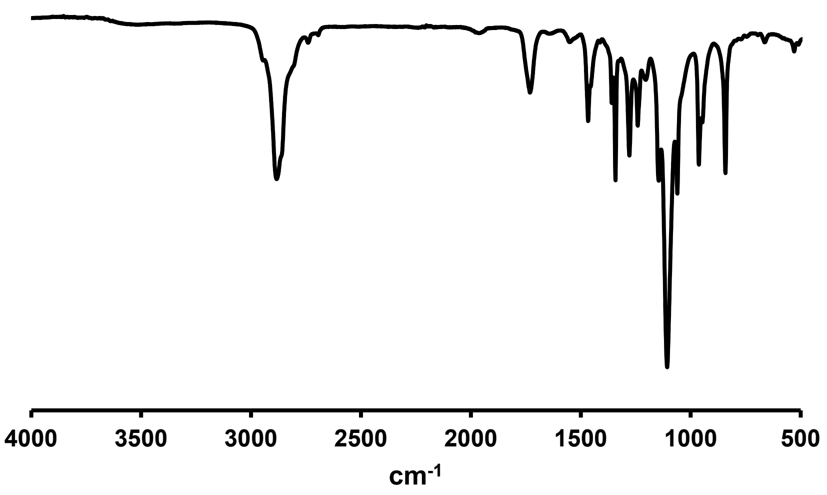

IR spectrum of PEG-[G2]-Cl

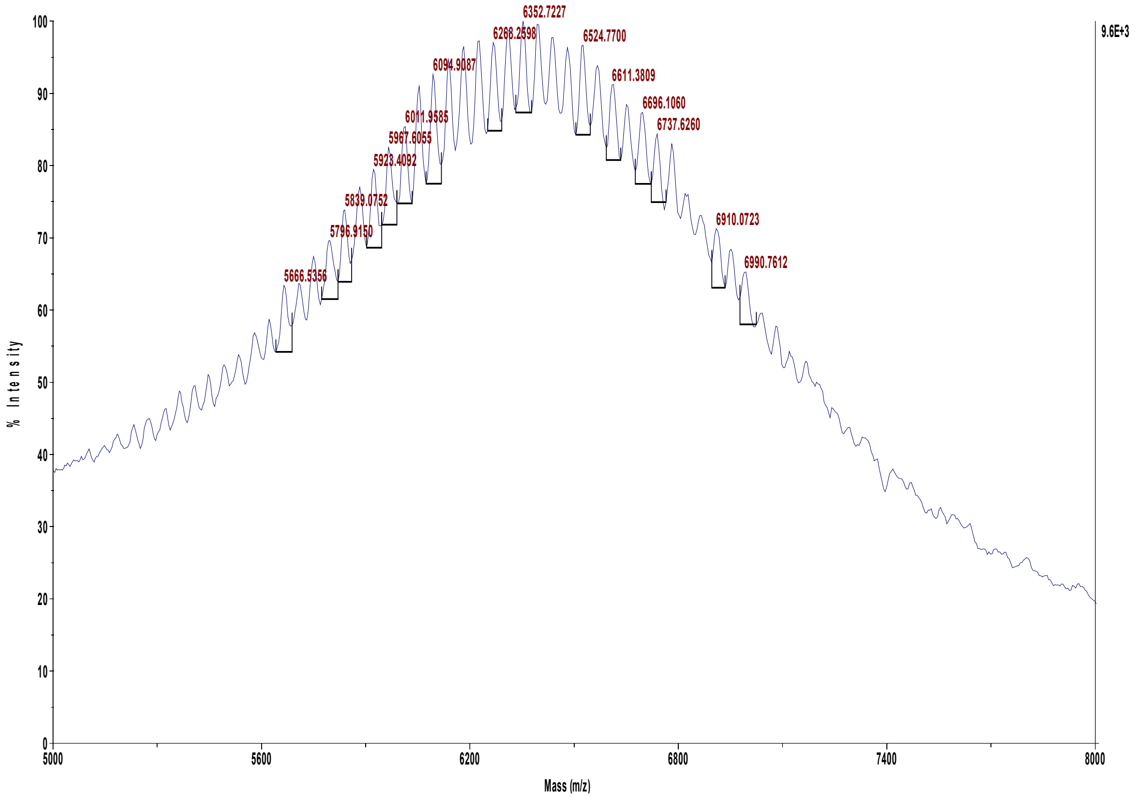

MALDI-TOF spectrum of PEG-[G2]-Cl

PEG-[G2]-N_3. A suspension of PEG-[G2]-Cl (500 mg, 0.078 mmol), NaN_3 (121 mg, 1.86 mmol), 15-crown-5 (6 mg, 0.03 mmol), and molecular sieves (4Å, 100 mg) in dry DMSO (3.1 mL) was stirred under Ar at rt for 5 h and then, heated at 80 °C for 8 h. The resulting suspension was diluted with $CHCl_3$ to double the original volume and filtered. The filtrate was diluted with $CHCl_3$ (100 mL), washed with MilliQ H_2O (3 x 100 mL) and brine (1 x 100 mL), dried, and concentrated. The obtained residue was precipitated from MeOH/*i*PrOH (-20 °C) to give PEG-[G2]-N_3 (473 mg, 94%) as a pale-yellow solid. ^{1}H NMR (500 MHz, $CDCl_3$) δ: 5.35 (bs, 1H), 4.86 – 4.76 (m, 4H), 4.65 (t, J = 6.8 Hz, 2H), 4.56 – 4.42 (m, 12H), 4.22 (t, J = 4.6 Hz, 2H), 3.94 – 3.43 (m, ~498H), 3.40 – 3.33 (m, 11H), 3.19 (q, J = 6.3 Hz, 2H), 2.14 (quint, J = 6.7 Hz, 2H). IR (ATR, cm^{-1}): 2885, 2110, 1732, 1108.

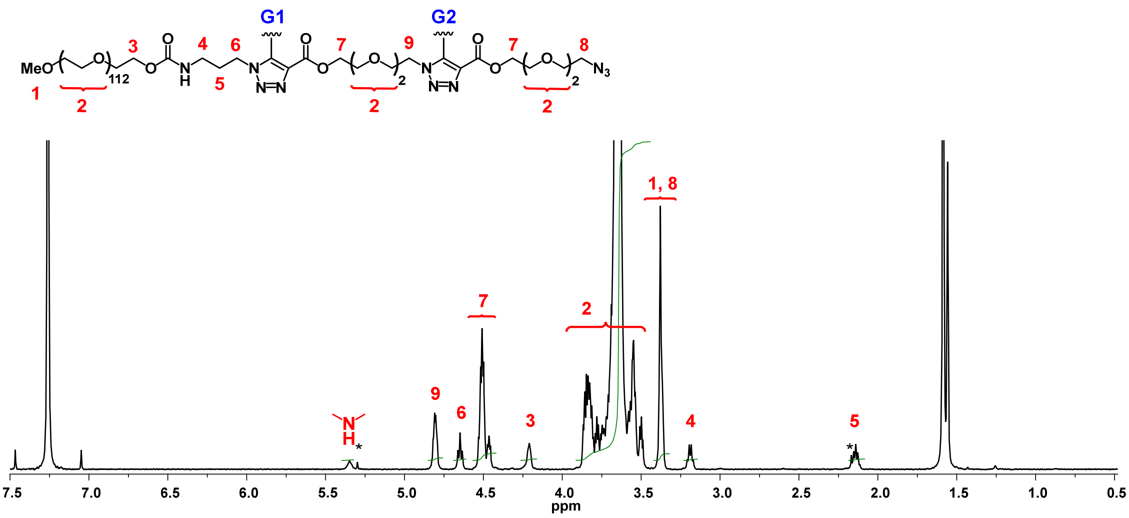

^{1}H NMR spectrum of PEG-[G2]-N_3 ($CDCl_3$)

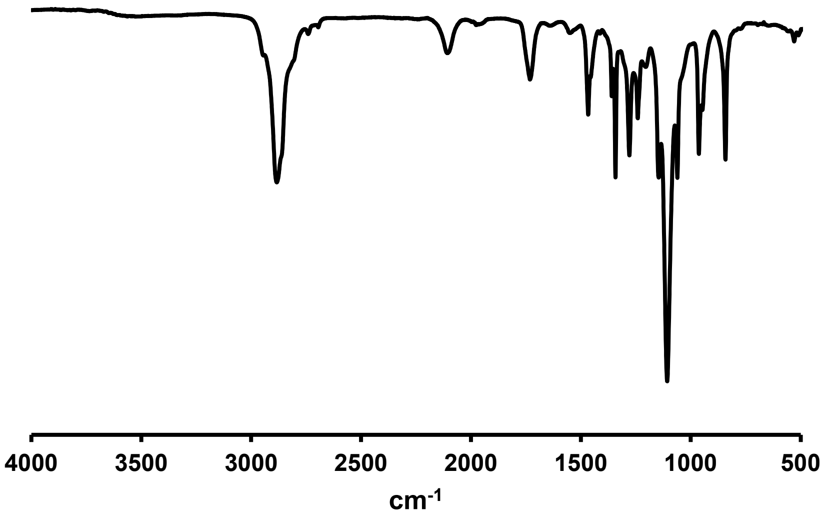

IR spectrum of PEG-[G2]-N_3

PEG-[G3]-Cl. A solution of PEG-[G2]-N_3 (452 mg, 0.070 mmol) and ADC-TEG-Cl (290 mg, 0.70 mmol) in $CHCl_3$ (2.8 mL) was heated under microwave radiation (60 ºC, 20 W, 14 psi airflow) for 4.5 h. Then, the reaction mixture was concentrated and precipitated from CH_2Cl_2/Et_2O (-20 °C) to give PEG-[G3]-Cl (550 mg, 97%) as a white solid. ^1H NMR (500 MHz, $CDCl_3$) δ: 5.35 (bs, 1H), 4.80 (s, 12H), 4.64 (t, J = 6.8 Hz, 2H), 4.57 – 4.40 (m, 28H), 4.26 – 4.18 (m, 2H), 3.95 – 3.45 (m, ~578H), 3.38 (s, 3H), 3.19 (q, J = 6.7 Hz, 2H), 2.14 (quint, J = 6.7 Hz, 2H). ^{13}C NMR (75 MHz, $CDCl_3$) δ: 159.6, 157.9, 155.8, 139.8, 139.4, 139.3, 138.9, 131.3, 130.9, 129.8, 71.3, 70.8, 70.1, 68.9, 68.3, 68.1, 65.1, 64.2, 63.3, 58.3, 49.5, 47.5, 41.8, 36.6, 29.2. IR (ATR, cm^{-1}): 2885, 1732, 1113.

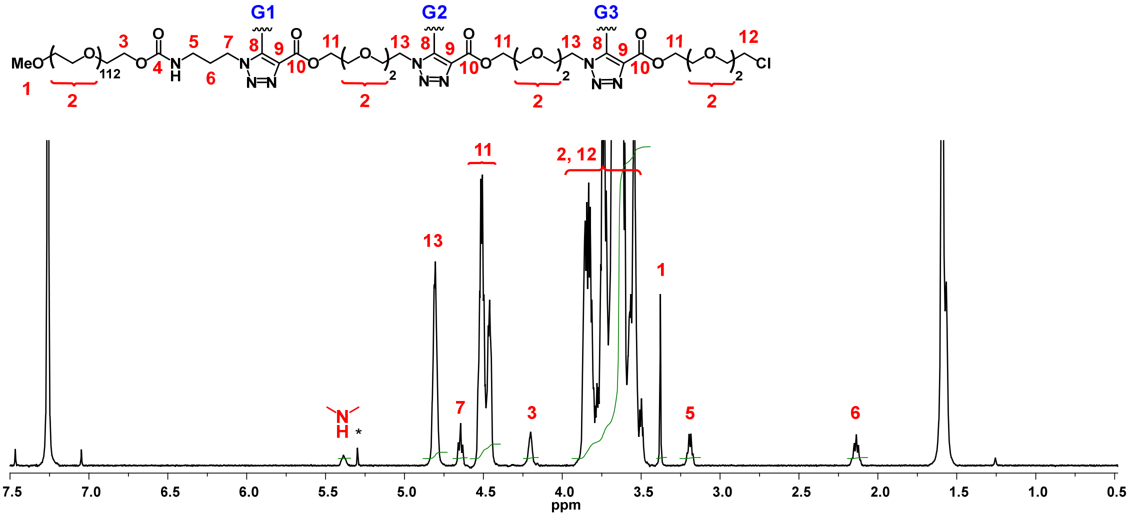

^1H NMR spectrum of PEG-[G3]-Cl ($CDCl_3$)

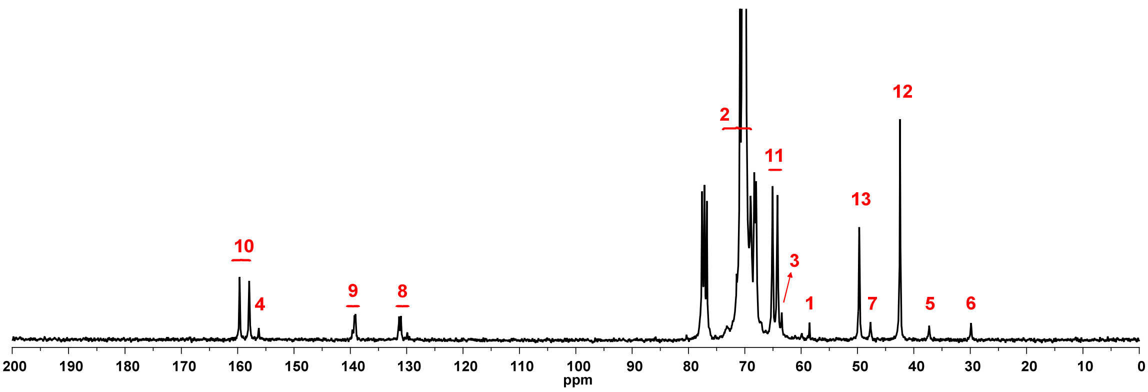

^{13}C NMR spectrum of PEG-[G3]-Cl ($CDCl_3$)

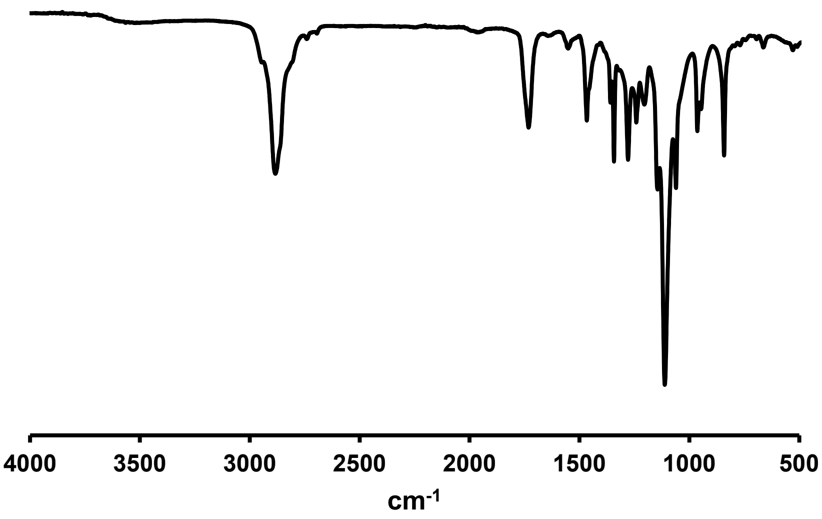

IR spectrum of PEG-[G3]-Cl

PEG-[G3]-N_3. A suspension of PEG-[G3]-Cl (500 mg, 0.062 mmol), NaN_3 (192 mg, 2.95 mmol), 15-crown-5 (11 mg, 0.049 mmol), and molecular sieves (4Å, 100 mg) in dry DMSO (3.1 mL) was stirred under Ar at rt for 5 h and then, heated at 80 °C for 8 h. The resulting suspension was diluted with $CHCl_3$ to double the original volume and filtered. The filtrate was diluted with $CHCl_3$ (100 mL), washed with MilliQ H_2O (3 x 100 mL) and brine (1 x 100 mL), dried, and concentrated. The obtained residue was precipitated from MeOH/*i*PrOH (-20 °C) to give PEG-[G3]-N_3 (452 mg, 90%) as a pale-yellow solid. ^1H NMR (500 MHz, $CDCl_3$) δ: 4.85 – 4.77 (m, 12H), 4.64 (t, *J* = 6.8 Hz, 2H), 4.57 – 4.40 (m, 28H), 4.23 – 4.16 (m, 2H), 3.95 – 3.45 (m, 560H), 3.42 – 3.33 (m, 19H), 3.19 (q, *J* = 6.7 Hz, 2H), 2.14 (quint, *J* = 6.7 Hz, 2H). IR (ATR, cm^{-1}): 2881, 2102, 1729, 1104.

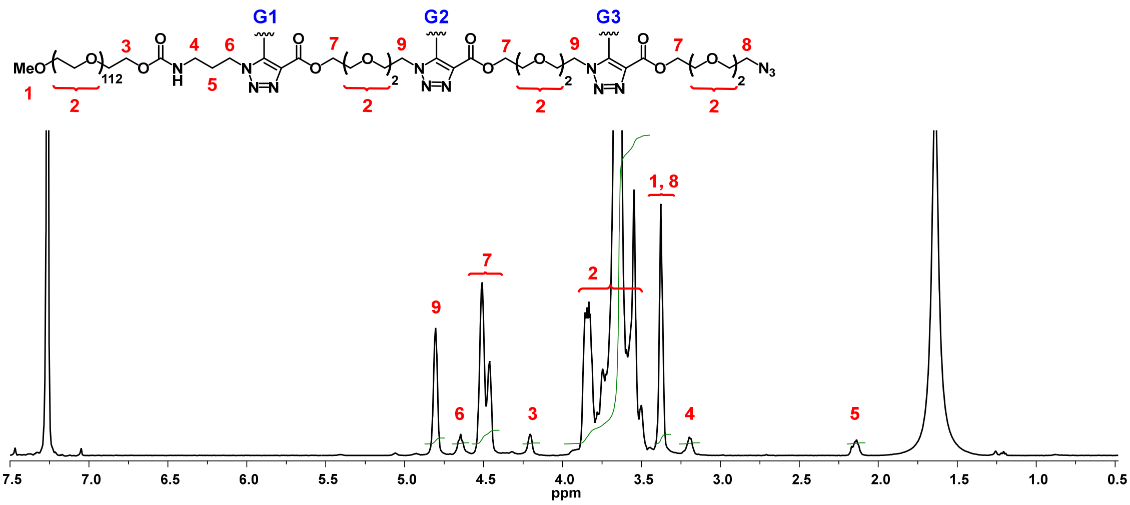

^{1}H NMR spectrum of PEG-[G3]-N_3 ($CDCl_3$)

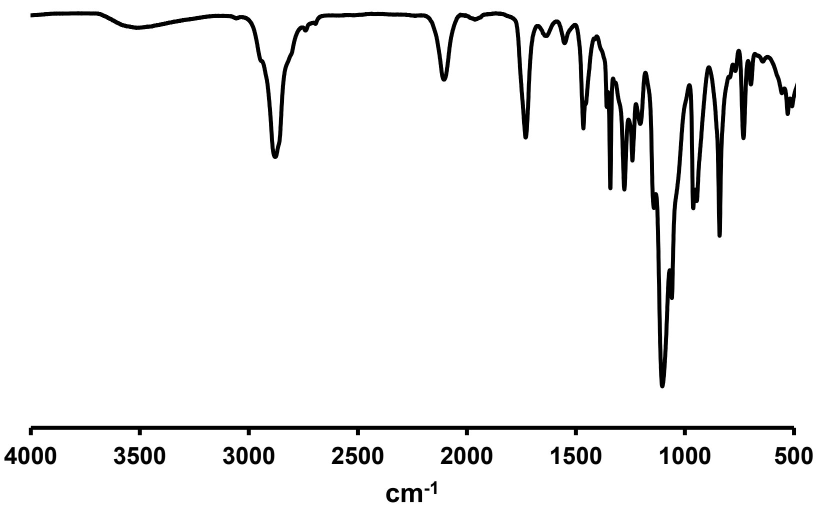

IR spectrum of PEG-[G3]-N_3

PEG-[G4]-Cl. A solution of PEG-[G3]-N_3 (452 mg, 0.055 mmol) and ADC-TEG-Cl (460 mg, 1.11 mmol) in $CHCl_3$ (4.4 mL) was heated under microwave radiation (60 ºC, 20 W, 14 psi airflow) for 6 h. Then, the reaction mixture was concentrated and precipitated from CH_2Cl_2/Et_2O (-20 °C) to give PEG-[G4]-Cl (606 mg, 93%) as a yellow solid. ^{1}H NMR (500 MHz, $CDCl_3$) δ: 5.40 (bs, 1H), 4.84 – 4.76 (m, 28H), 4.64 (t, J = 6.9 Hz, 2H), 4.56 – 4.41 (m, 60H), 4.26 – 4.18 (m, 2H), 3.95 – 3.45 (m, ~722H), 3.38 (s, 3H), 3.18 (q, J = 6.3 Hz, 2H), 2.13 (quint, J = 6.7 Hz, 2H). ^{13}C NMR (75 MHz, $CDCl_3$) δ: 160.1, 158.4, 156.7, 140.2, 139.8, 139.6, 131.7, 131.4, 71.9, 71.3, 70.5, 69.4, 69.2, 68.8, 68.6, 68.5, 68.4, 65.5, 64.6, 63.9, 59.0, 50.1, 48.1, 42.8, 37.6, 30.2, 29.6. IR (ATR, cm^{-1}): 2868, 1732, 1105.

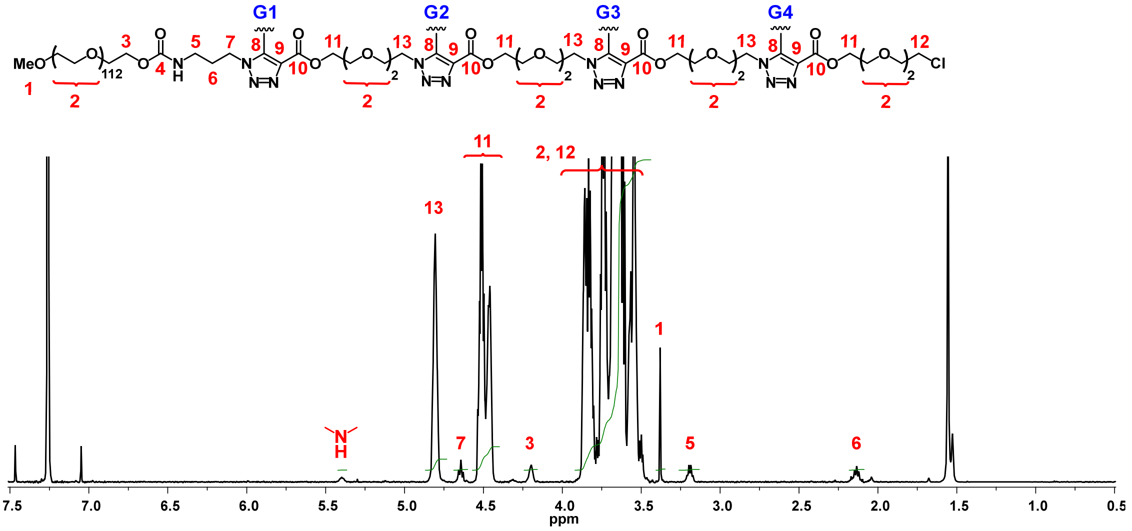

^{1}H NMR spectrum of PEG-[G4]-Cl ($CDCl_3$)

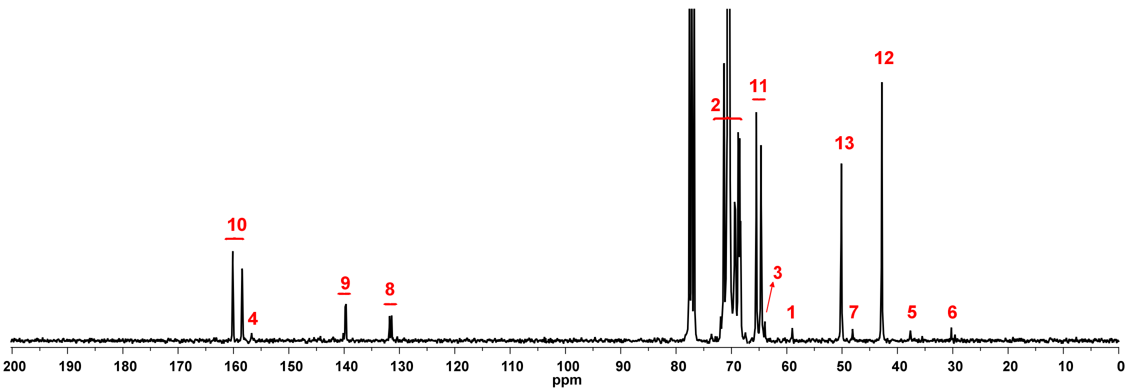

^{13}C NMR spectrum of PEG-[G4]-Cl ($CDCl_3$)

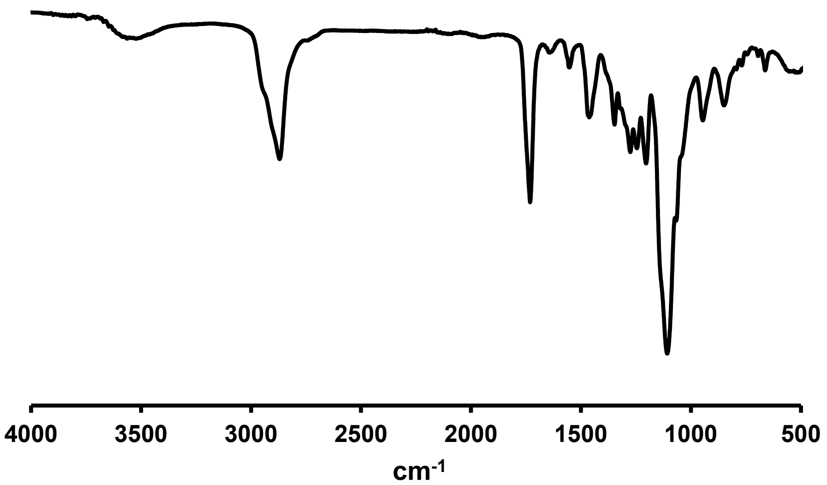

IR spectrum of PEG-[G4]-Cl

PEG-[G4]-N_3. A suspension of PEG-[G4]-Cl (500 mg, 0.042 mmol), NaN_3 (265 mg, 4.07 mmol), 15-crown-5 (15 mg, 0.068 mmol), and molecular sieves (4Å, 100 mg) in dry DMSO (6.8 mL) was stirred under Ar at rt for 5 h and then, heated at 80 °C for 8 h. The resulting suspension was diluted with $CHCl_3$ to double the original volume and filtered. The filtrate was diluted with $CHCl_3$ (100 mL), washed with MilliQ H_2O (3 x 100 mL) and brine (1 x 100 mL), dried, and concentrated. The obtained residue was precipitated from MeOH/*i*PrOH (-20 °C) to give PEG-[G4]-N_3 (463 mg, 92%) as a pale-yellow solid. ^1H NMR (500 MHz, $CDCl_3$) δ: 4.86 – 4.75 (m, 28H), 4.64 (t, *J* = 6.9 Hz, 2H), 4.56 – 4.41 (m, 60H), 4.23 – 4.15 (m, 2H), 3.95 – 3.45 (m, ~690H), 3.42 – 4.30 (m, 35H), 3.19 (q, *J* = 6.3 Hz, 2H), 2.13 (quint, *J* = 6.7 Hz, 2H). IR (ATR, cm^{-1}): 2874, 2106, 1729, 1105.

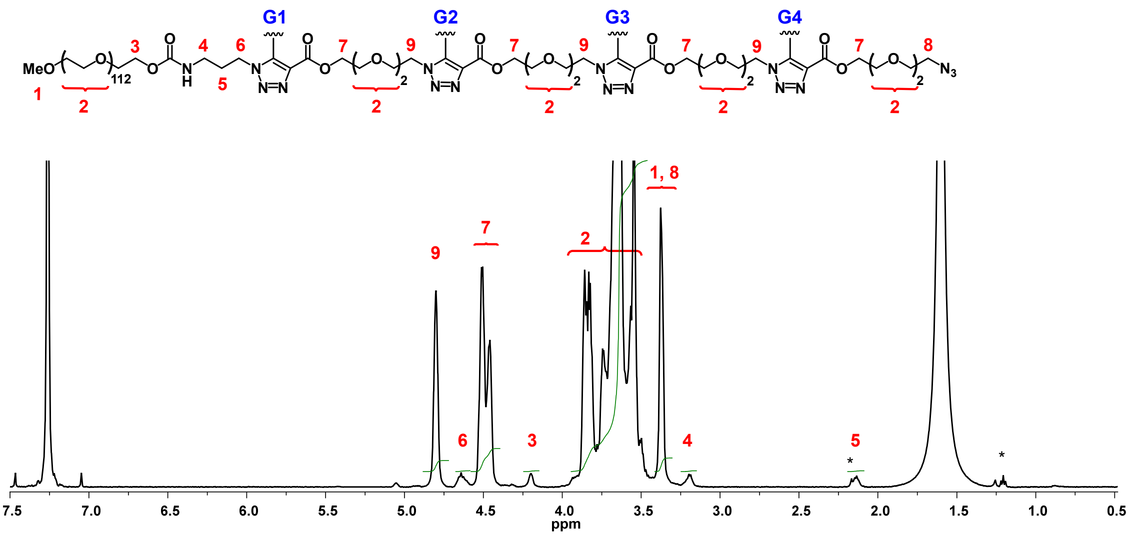

^{1}H NMR spectrum of PEG-[G4]-N_3 ($CDCl_3$)

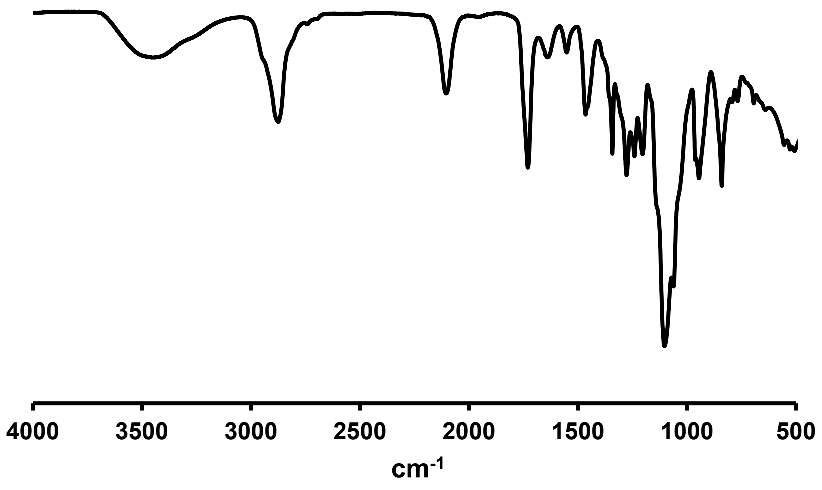

IR spectrum of PEG-[G4]-N_3

PEG-[G5]-Cl. A solution of PEG-[G4]-N_3 (457 mg, 0.038 mmol) and ADC-TEG-Cl (640 mg, 1.54 mmol) in $CHCl_3$ (6.1 mL) was heated under microwave radiation (60 ºC, 20 W, 14 psi airflow) for 10 h. Then, the reaction mixture was concentrated and precipitated from CH_2Cl_2/Et_2O (-20 °C) to give PEG-[G5]-Cl (645 mg, 91%) as a yellow solid. ^{1}H NMR (500 MHz, $CDCl_3$) δ: 4.86 – 4.75 (m, 60H), 4.69 – 4.58 (m, 2H), 4.56 – 4.41 (m, 124H), 4.22 – 4.16 (m, 2H), 3.95 – 3.45 (m, ~1010H), 3.38 (s, 3H), 3.19 (q, J = 6.3 Hz, 2H), 2.13 (quint, J = 6.7 Hz, 2H). ^{13}C NMR (126 MHz, $CDCl_3$) δ: 159.8, 158.1, 139.5, 139.4, 131.5, 131.2, 71.2, 70.4, 702, 69.2, 69.1, 68.6, 68.5, 68.3, 68.2, 65.4, 64.5, 58.8, 53.5, 50.0, 42.7. IR (ATR, cm^{-1}): 2872, 1731, 1105.

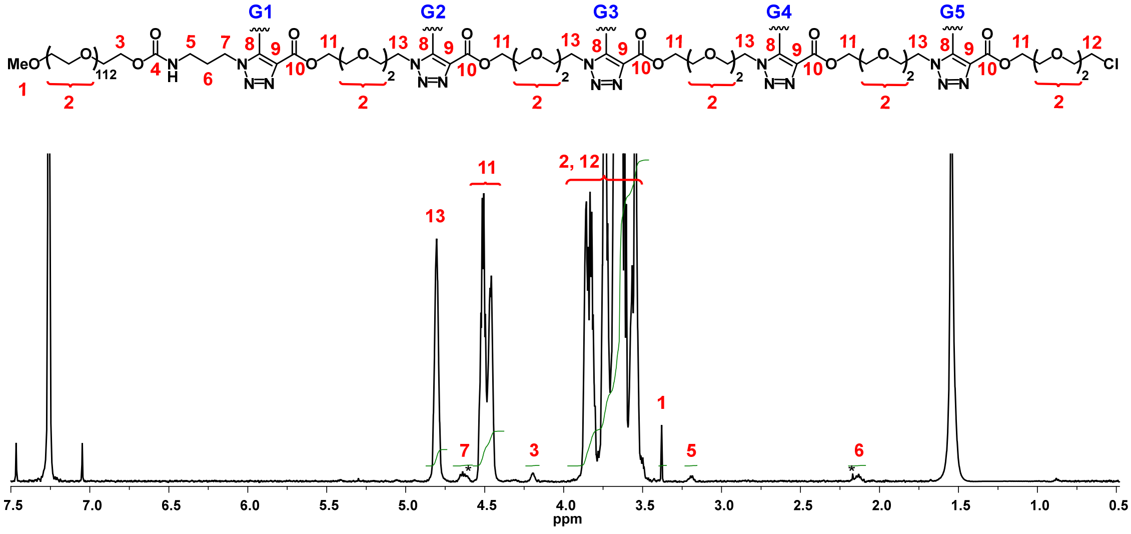

^{1}H NMR spectrum of PEG-[G5]-Cl ($CDCl_3$)

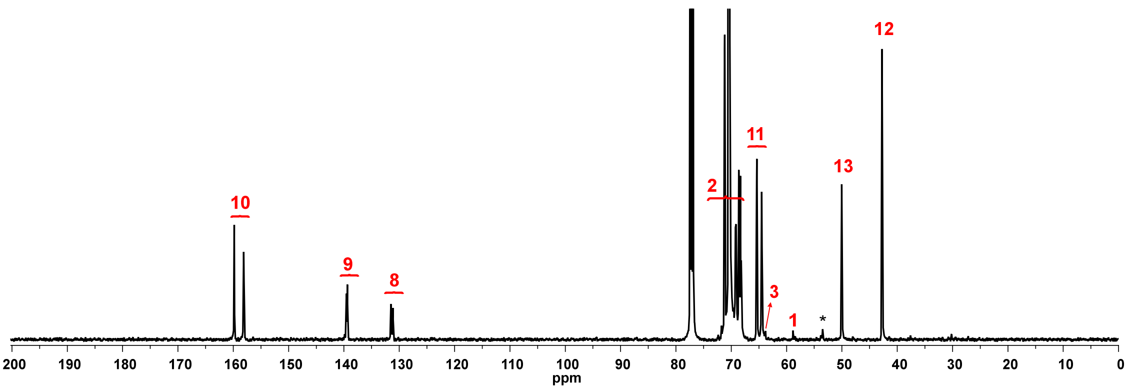

^{13}C NMR spectrum of PEG-[G5]-Cl ($CDCl_3$)

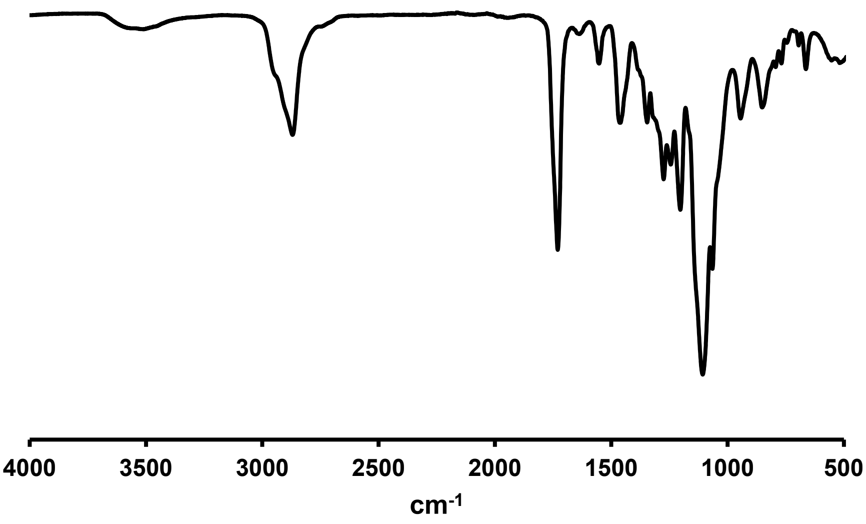

IR spectrum of PEG-[G5]-Cl

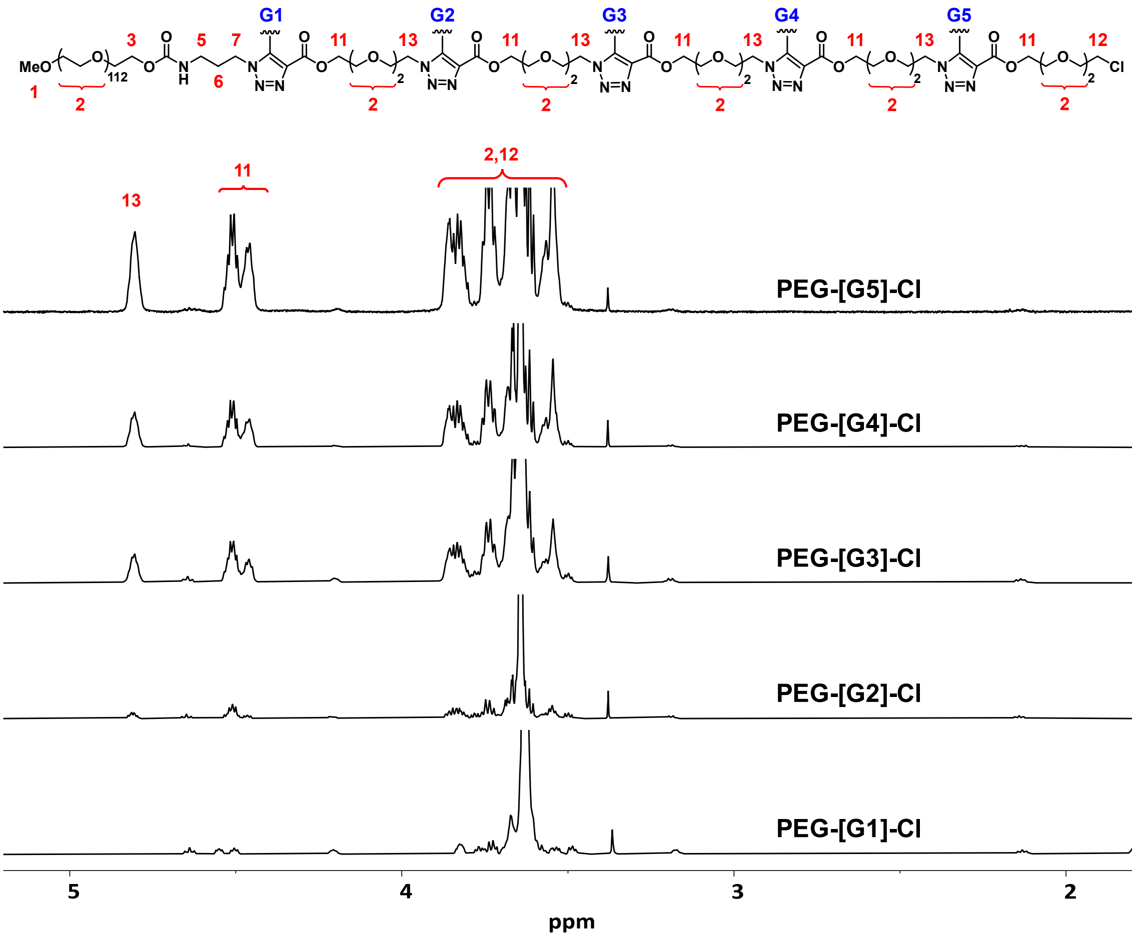

Figure S1. Monitoring the dendritic growth of five generations of AAC-based PEG-dendritic block copolymers by ^{1}H NMR ($CDCl_3$)

PEG-[G3]-Et. A solution of PEG-[G2]-N_3 (110 mg, 0.017 mmol) and ADC-Et (29 mg, 0.17 mmol) in $CHCl_3$ (0.7 mL) was heated under microwave radiation (60 ºC, 20 W, 14 psi airflow) for 7 h. Then, the reaction mixture was concentrated and precipitated from MeOH/*i*PrOH (-20 °C) to give PEG-[G3]-Et (111 mg, 91%) as a white solid. ^{1}H NMR (500 MHz, $CDCl_3$) δ: 5.39 (bs, 1H), 4.84 – 4.74 (m, 12H), 4.64 (t, *J* = 6.9 Hz, 2H), 4.57 – 4.35 (m, 28H), 4.23 – 4.18 (m, 2H), 3.98 – 3.44 (m, ~498H), 3.38 (s, 3H), 3.27 – 3.18 (q, *J* = 6.3 Hz, 2H), 2.14 (quint, *J* = 6.7 Hz, 2H), 1.49 – 1.33 (m, 24H). ^{13}C NMR (126 MHz, $CDCl_3$) δ: 160.1, 159.8, 158.5, 158.1, 156.4, 140.0, 139.8, 139.7, 139.5, 131.5, 131.4, 131.2, 131.1, 131.0, 130.1, 71.9, 71.6, 71.2, 70.4, 69.7, 69.3, 69.2, 68.6, 68.2, 65.5, 65.4, 64.6, 64.5, 63.9, 62.7, 61.7, 61.7, 58.9, 50.1, 49.9, 48.0, 37.6, 30.6, 29.9, 14.1, 13.8. IR (ATR, cm^{-1}): 2884, 1732, 1106.

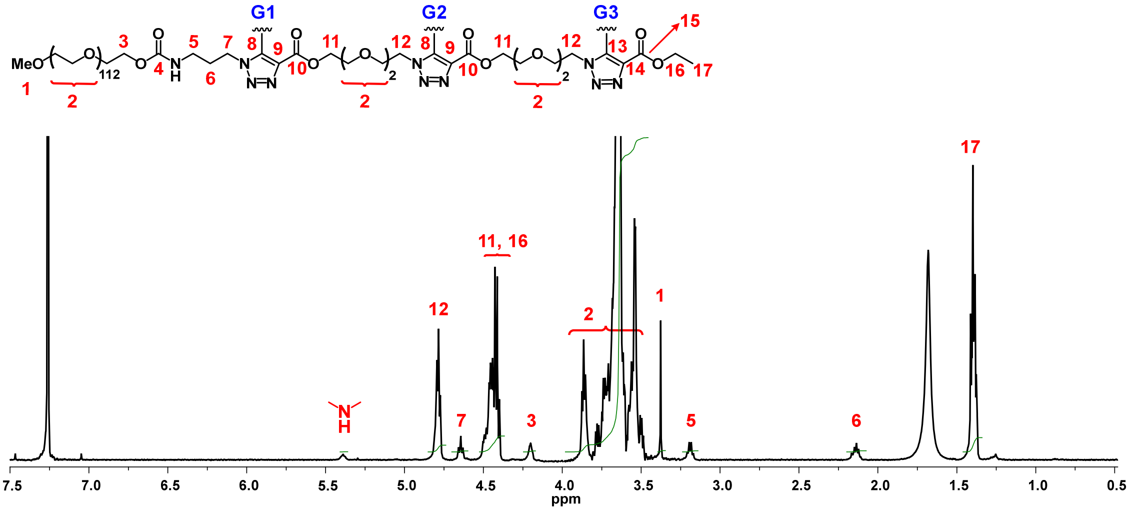

^{1}H NMR spectrum of PEG-[G3]-Et ($CDCl_3$)

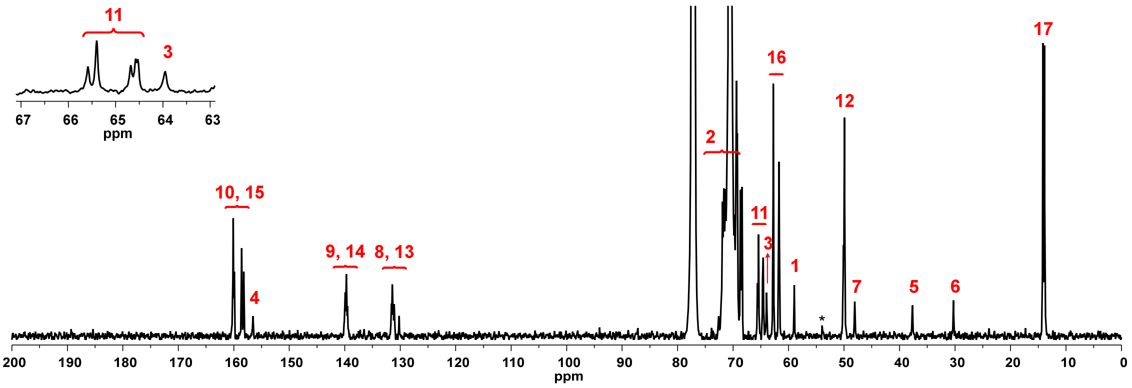

^{13}C NMR spectrum of PEG-[G3]-Et (CDCl$_3$)

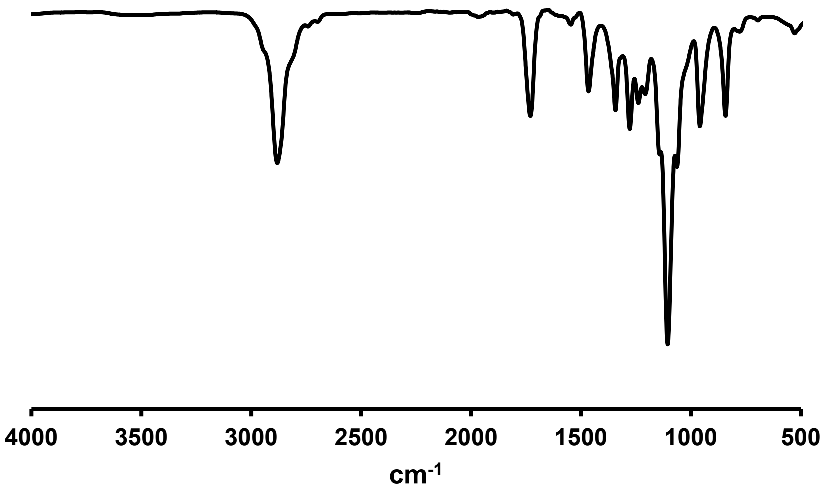

IR spectrum of PEG-[G3]-Et

PEG-[G3]-Dod. A solution of PEG-[G2]-N_3 (100 mg, 0.015 mmol) and ADC-Dod (69 mg, 0.15 mmol) in $CHCl_3$ (0.6 mL) was heated under microwave radiation (60 ºC, 20 W, 14 psi airflow) for 7 h. Then, the reaction mixture was concentrated and precipitated from MeOH/*i*PrOH (-20 °C) to give PEG-[G3]-Dod (110 mg, 86%) as a white solid. ^{1}H NMR (500 MHz, $CDCl_3$) δ: 5.37 (bs, 1H), 4.88 – 4.70 (m, 12H), 4.64 (t, *J* = 7.0 Hz, 2H), 4.57 – 4.42 (m, 12H), 4.34 (t, *J* = 7.0 Hz, 16H), 4.23 – 4.17 (m, 2H), 3.94 – 3.43 (m, ~498H), 3.38 (s, 3H), 3.19 (q, *J* = 6.4 Hz, 2H), 2.14 (quint, 6.7 Hz, 2H), 1.82 – 1.71 (m, 16H), 1.48 – 1.17 (m, 144H), 0.88 (t, *J* = 6.8 Hz, 24H). ^{13}C NMR (126 MHz, $CDCl_3$) δ: 160.3, 159.9, 158.6, 158.2, 156.5, 140.0, 139.9, 131.6, 131.1, 130.8, 130.2, 77.4, 77.4, 77.2, 76.9, 71.9, 70.5, 69.4, 69.2, 68.7, 68.4, 66.9, 66.0, 6599, 65.6, 65.4, 64.7, 64.5, 63.9, 59.0, 49.9, 48.1, 37.6, 31.9, 30.3, 29.6, 29.5, 29.3, 29.2, 28.5, 28.3, 25.8, 22.6, 14.1. IR (ATR, cm^{-1}): 2885, 1732, 1103.

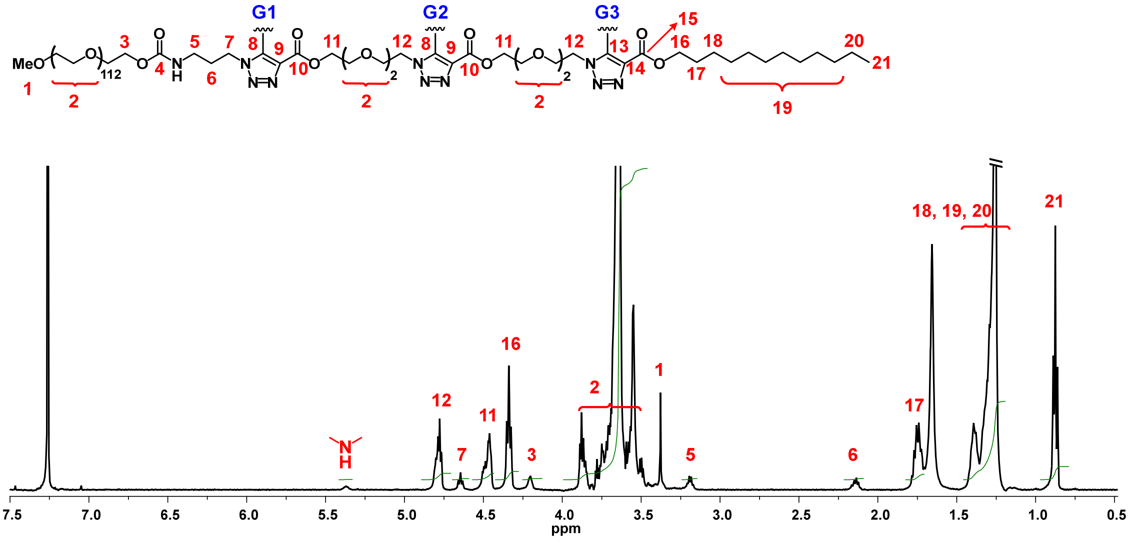

^{1}H NMR spectrum of PEG-[G3]-Dod ($CDCl_3$)

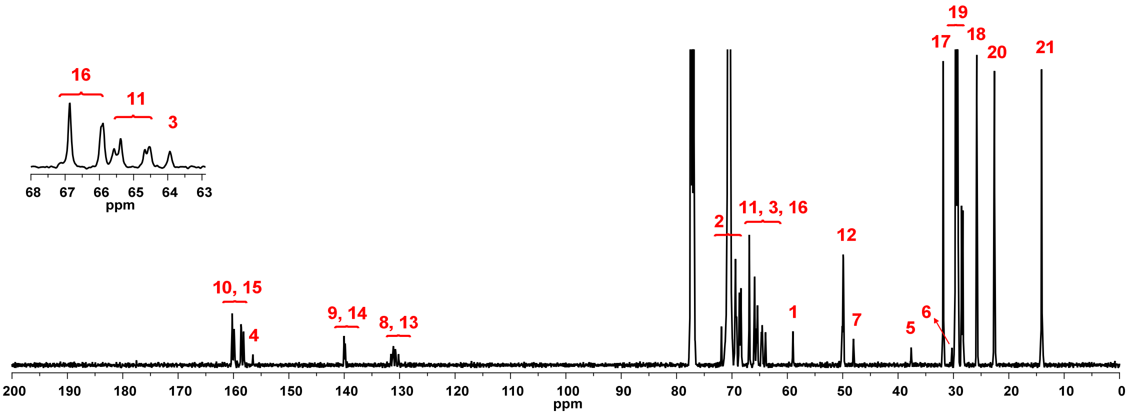

^{13}C NMR spectrum of PEG-[G3]-Dod ($CDCl_3$)

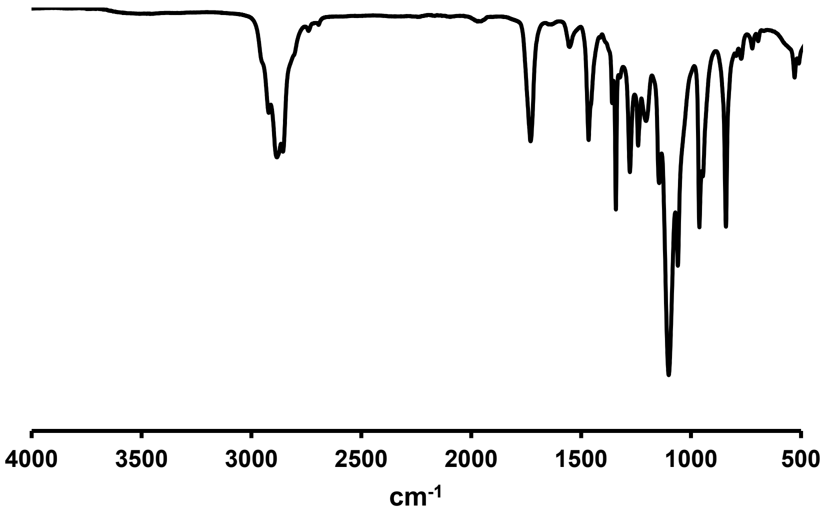

IR spectrum of PEG-[G3]-Dod

PEG-[G3]-Ar. A solution of PEG-[G2]-N_3 (150 mg, 0.023 mmol) and ADC-Ar (87 mg, 0.23 mmol) in $CHCl_3$ (0.9 mL) was heated under microwave radiation (60 ºC, 20 W, 14 psi airflow) for 7 h. Then, the reaction mixture was concentrated and precipitated from MeOH/*i*PrOH (-20 °C) to give PEG-[G3]-Ar (169 mg, 91%) as a white solid. ^1H NMR (500 MHz, $CDCl_3$) δ: 7.29 – 7.23 (m, 8H), 7.19 – 7.14 (m, 24H), 5.35 (bs, 1H), 4.84 – 4.70 (m, 12H), 4.63 (t, *J* = 7.0 Hz, 2H), 4.53 – 4.39 (m, 12H), 4.39 – 4.28 (m, 16H), 4.24 – 4.16 (m, 2H), 3.92 – 3.45 (m, ~498H), 3.38 (s, 3H), 3.17 (q, *J* = 6.4 Hz, 2H), 2.64 (t, *J* = 7.1 Hz, 16H), 2.12 (quint, 6.7 Hz, 2H), 1.84 – 1.65 (m, 32H). ^{13}C NMR (126 MHz, $CDCl_3$) δ: 159.9, 159.7, 159.6, 158.3, 157.9, 156.2, 141.5, 141.4, 141.3, 139.6, 139.4, 139.2, 131.3, 131.0, 130.7, 130.6, 129.9, 129.7, 128.7, 128.5, 128.1, 127.5, 125.6, 125.5, 71.6, 71.4, 71.1, 70.8, 70.3, 70.0, 69.9, 69.7, 69.5, 69.2, 69.1, 68.9, 68.4, 68.1, 66.2, 65.3, 65.1, 64.4, 64.2, 63.6, 58.7, 49.8, 49.7, 49.5, 37.4, 35.0, 30.0, 27.8, 27.6, 27.3, 27.2. IR (ATR, cm^{-1}): 2876, 1732, 1106.

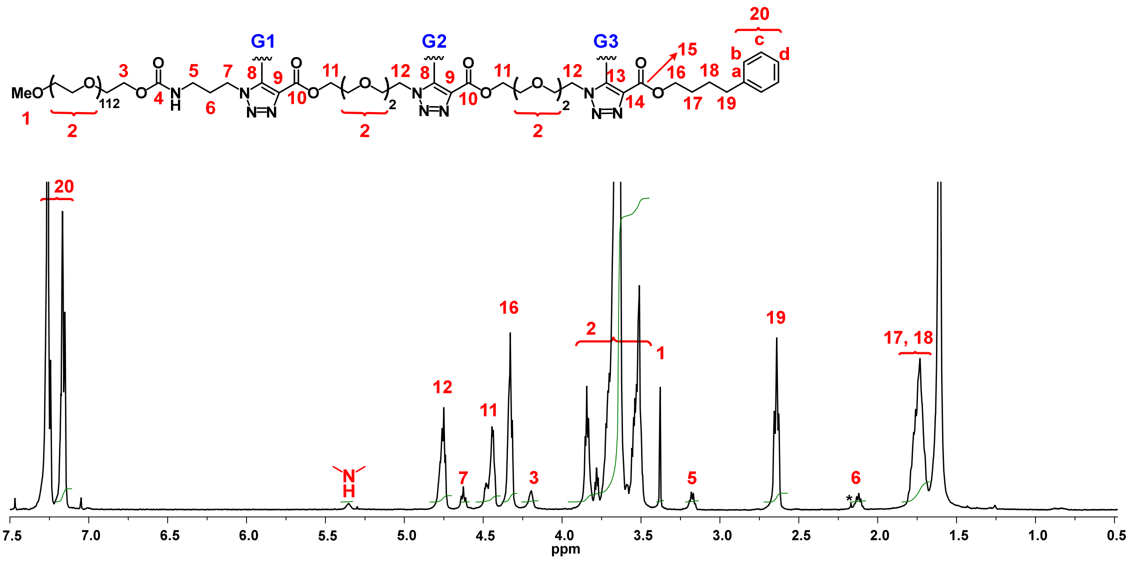

^1H NMR spectrum of PEG-[G3]-Ar ($CDCl_3$)

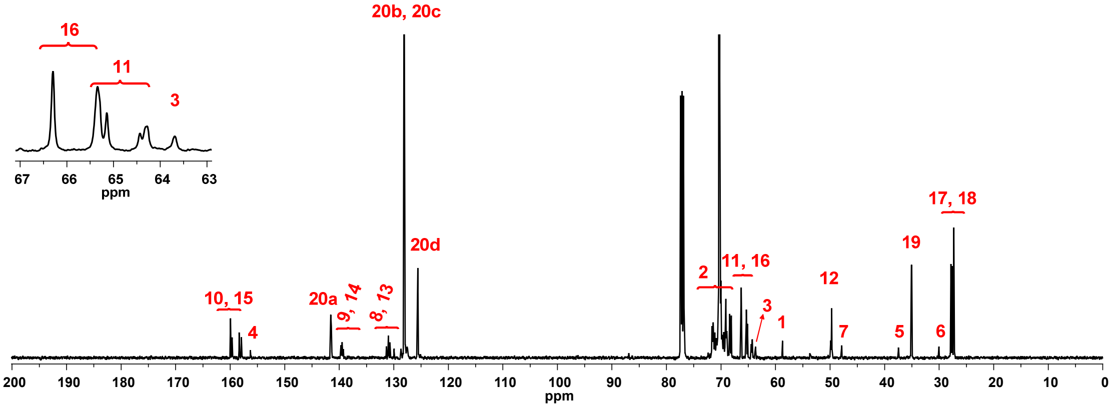

^{13}C NMR spectrum of PEG-[G3]-Ar ($CDCl_3$)

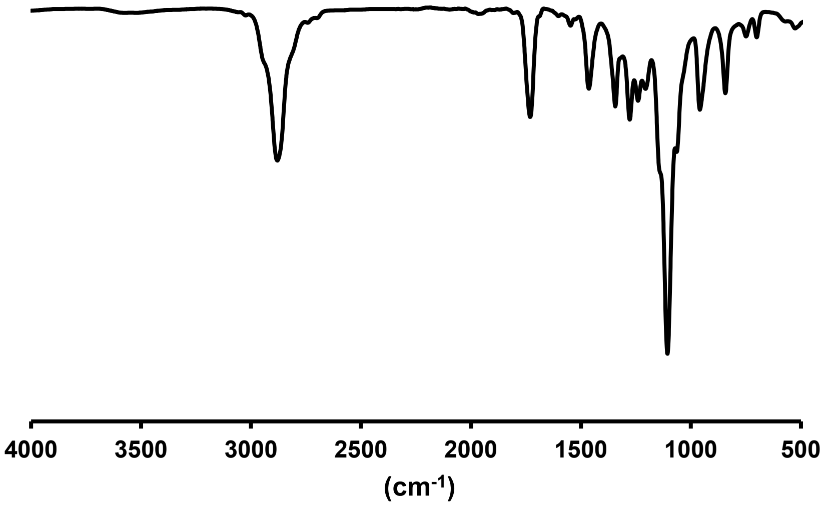

IR spectrum of PEG-[G3]-Ar

PEG-[G3]-Br. A solution of PEG-[G2]-N_3 (150 mg, 0.023 mmol) and ADC-Br (82 mg, 0.23 mmol) in $CHCl_3$ (0.9 mL) was heated under microwave radiation (60 ºC, 20 W, 14 psi airflow) for 7 h. Then, the reaction mixture was concentrated and precipitated from MeOH/*i*PrOH (-20 °C) to give PEG-[G3]-Br (174 mg, 96%) as a white solid. ^{1}H NMR (500 MHz, $CDCl_3$) δ: 5.35 (bs, 1H), 4.85 – 4.76 (m, 12H), 4.64 (t, *J* = 7.0 Hz, 2H), 4.55 – 4.42 (m, 28H), 4.23-4.17 (m, 2H), 3.92 – 3.45 (m, ~514H), 3.38 (s, 3H), 3.19 (q, *J* = 6.4 Hz, 2H), 2.38 – 2.25 (m, 16H), 2.14 (quint, 6.7 Hz, 2H). ^{13}C NMR (126 MHz, $CDCl_3$) δ: 159.7, 158.2, 158.0, 156.4, 140.0, 139.7, 139.4, 139.2, 131.5, 131.3, 131.2, 131.1, 131.0, 130.9, 130.4, 129.7, 71.7, 71.5, 71.2, 70.9, 70.4, 69.6, 69.2, 69.1, 68.5, 68.2, 65.4, 65.2, 64.4, 64.2, 63.8, 63.3, 63.1, 58.8, 53.9, 53.6, 49.9, 47.9, 37.5, 31.4, 31.1, 31.1, 31.0, 30.1, 29.2. IR (ATR, cm^{-1}): 2876, 1732, 1104.

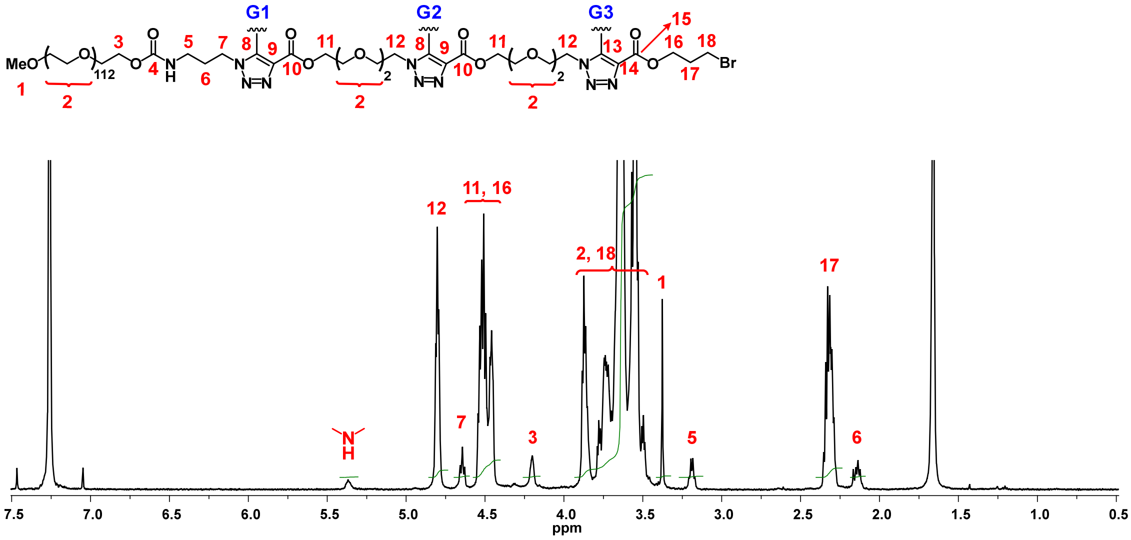

^{1}H NMR spectrum of PEG-[G3]-Br ($CDCl_3$)

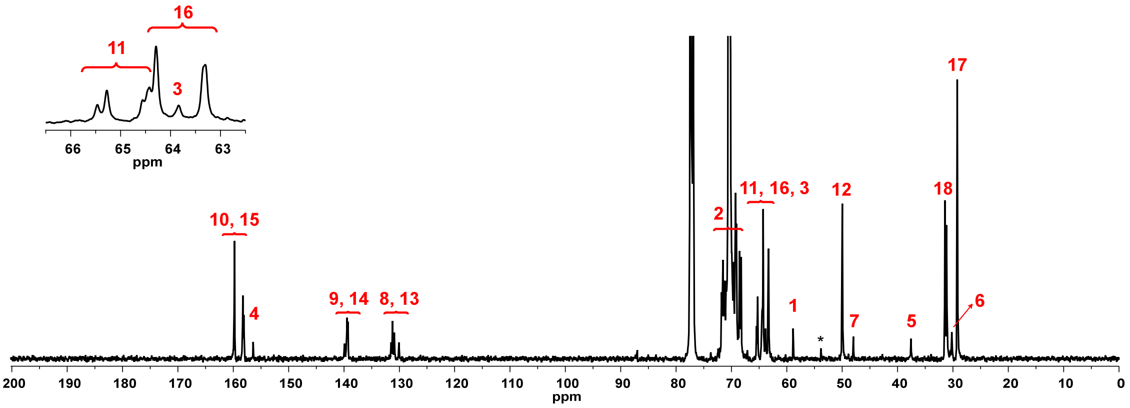

^{13}C NMR spectrum of PEG-[G3]-Br ($CDCl_3$)

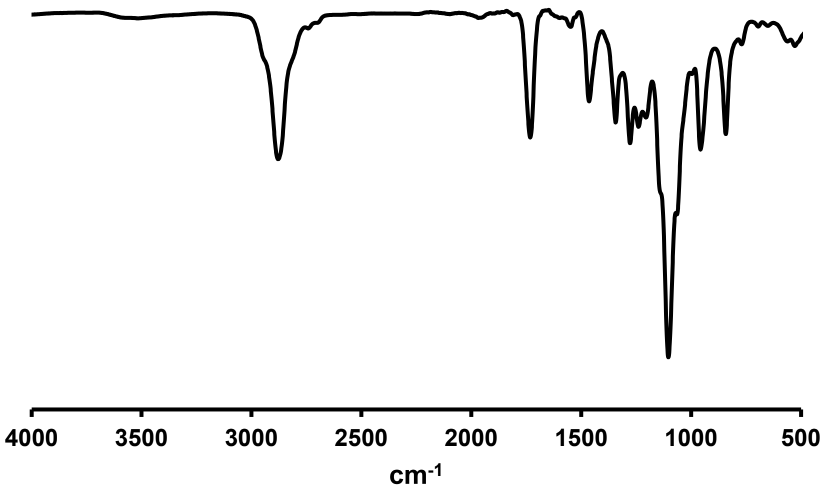

IR spectrum of PEG-[G3]-Br

PEG-[G3]-ene. A solution of PEG-[G2]-N_3 (537 mg, 0.083 mmol) and ADC-ene (161 mg, 0.83 mmol) in $CHCl_3$ (3.3 mL) was heated under microwave radiation (60 ºC, 20 W, 14 psi airflow) for 7 h. Then, the reaction mixture was concentrated and precipitated from MeOH/*i*PrOH (-20 °C) to give PEG-[G3]-ene (548 mg, 91%) as a yellow solid. ^{1}H NMR (500 MHz, $CDCl_3$) δ: 6.08 – 5.90 (m, 8H), 5.46 – 5.36 (m, 8H), 5.35 – 5.24 (m, 8H), 4.90 – 4.75 (m, 12H), 4.64 (t, *J* = 6.9 Hz, 2H), 4.53 – 4.40 (m, 28H), 4.23 – 4.16 (m, 2H), 3.89 – 3.44 (m, ~498H), 3.38 (s, 3H), 3.19 (q, *J* = 6.4 Hz, 2H), 2.13 (quint, *J* = 6.7 Hz, 2H). ^{13}C NMR (126 MHz, $CDCl_3$) δ: 159.8, 159.7, 158.2, 156.6, 156.2, 140.2, 139.7, 139.6, 139.5, 139.1, 131.6, 131.5, 131.4, 131.3, 131.1, 130.7, 130.6, 119.8, 119.7, 119.2, 119.1, 71.8, 71.1, 70.5, 69.9, 69.3, 69.2, 68.6, 68.3, 67.2, 66.2, 65.5, 65.3, 64.6, 64.5, 63.9, 58.9, 49.9, 48.2, 47.8, 37.8, 37.3, 30.3, 29.8. IR (ATR, cm^{-1}): 2882, 1729, 1104.

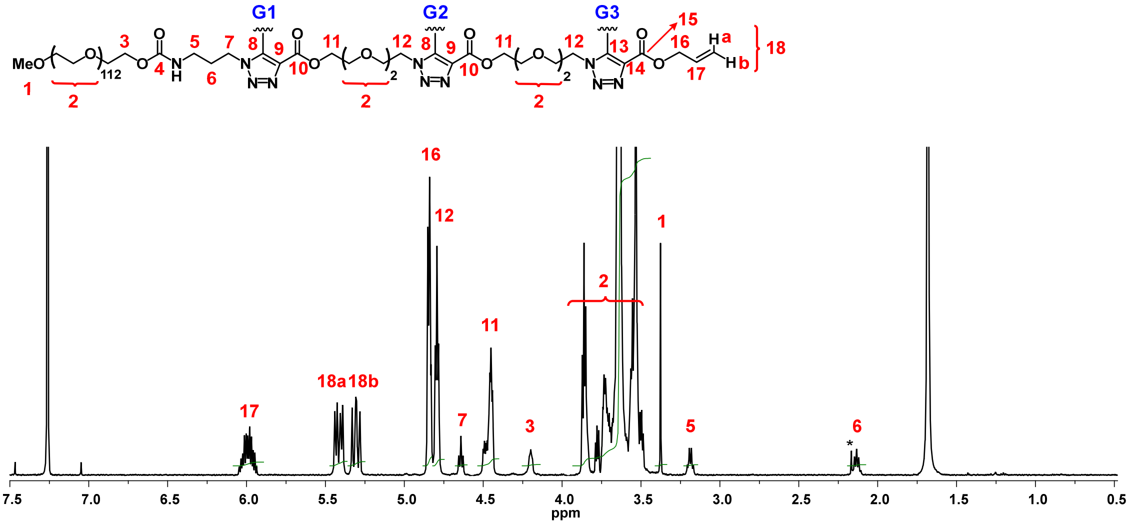

^{1}H NMR spectrum of PEG-[G3]-ene ($CDCl_3$)

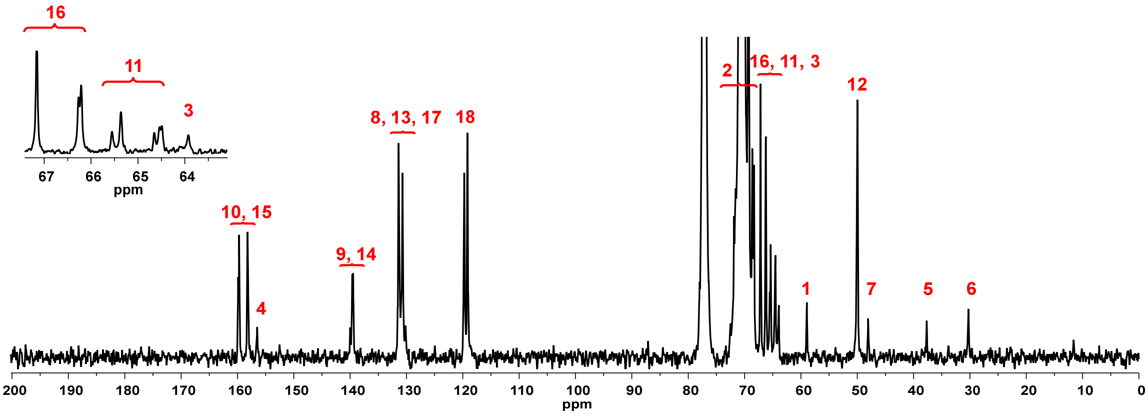

^{13}C NMR spectrum of PEG-[G3]-ene ($CDCl_3$)

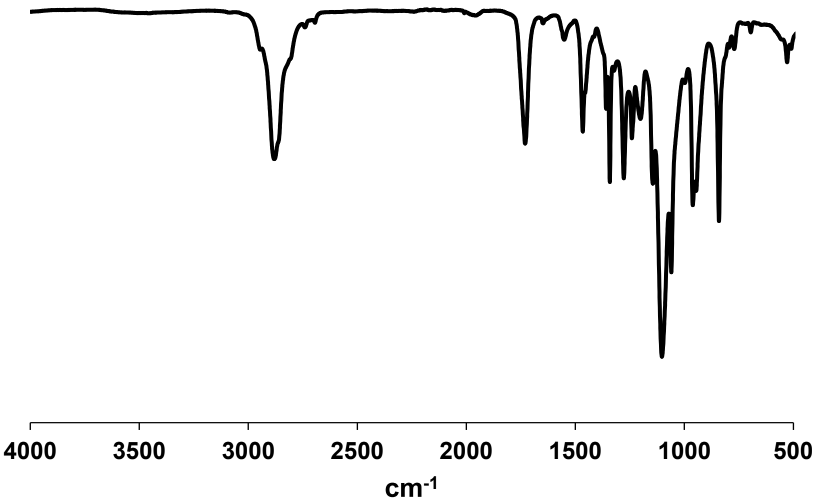

IR spectrum of PEG-[G3]-ene

PEG-[G3]-yne. A solution of PEG-[G2]-N_3 (321 mg, 0.049 mmol) and ADC-yne (94 mg, 0.49 mmol) in $CHCl_3$ (2 mL) was heated under microwave radiation (60 ºC, 20 W, 14 psi airflow) for 7 h. Then, the reaction mixture was concentrated and precipitated from MeOH/*i*PrOH (-20 °C) to give PEG-[G3]-yne (333 mg, 93%) as a yellow solid. ^{1}H NMR (500 MHz, $CDCl_3$) δ: 5.01 – 4.92 (m, 16H), 4.87 – 4.76 (m, 12H), 4.65 (t, J = 6.9 Hz, 2H), 4.54 – 4.41 (m, 12H), 4.23 – 4.16 (m, 2H), 3.91 – 3.44 (m, ~535H), 3.38 (s, 3H), 3.19 (q, J = 6.4 Hz, 2H), 2.68-3.61 (m, 4H), 2.60 – 2.50 (m, 4H), 2.13 (quint, J = 6.7 Hz, 2H). ^{1}H NMR (500 MHz, CD_3OD) δ: 5.05 (s, 8H), 5.00 (s, 8H), 4.90 – 4.81 (m, 12H), 4.69 (t, J = 6.9 Hz, 2H), 4.56 – 4.43 (m, 12H), 4.20 – 4.15 (m, 2H), 3.93 – 3.47 (m, ~498H), 3.38 (s, 3H), 3.20 (t, J = 6.3 Hz, 2H), 3.17 – 3.12 (m, 4H), 3.09 – 3.05 (m, 4H), 2.21 – 2.13 (m, 2H). ^{13}C NMR (126 MHz, $CDCl_3$) δ: 160.3, 159.4, 158.6, 158.5, 158.4, 158.1, 158.0, 156.9, 140.3, 139.9, 139.8, 139.5, 139.4, 139.3, 139.2, 131.9, 131.6, 131.5, 131.4, 131.2, 130.5, 88.1, 86.9, 77.0, 76.6, 76.5, 76.3, 76.2, 72.2, 72.0, 71.7, 71.4, 70.8, 70.5, 70.3, 70.0, 69.8, 69.6, 69.5, 68.9, 68.6, 65.9, 65.8, 64.9, 64.2, 59.3, 54.4, 54.3, 53.4, 53.3, 50.5, 50.0, 48.4, 38.1, 37.8, 30.6, 30.0. IR (ATR, cm-^{1}): 2868, 2128, 1737, 1104.

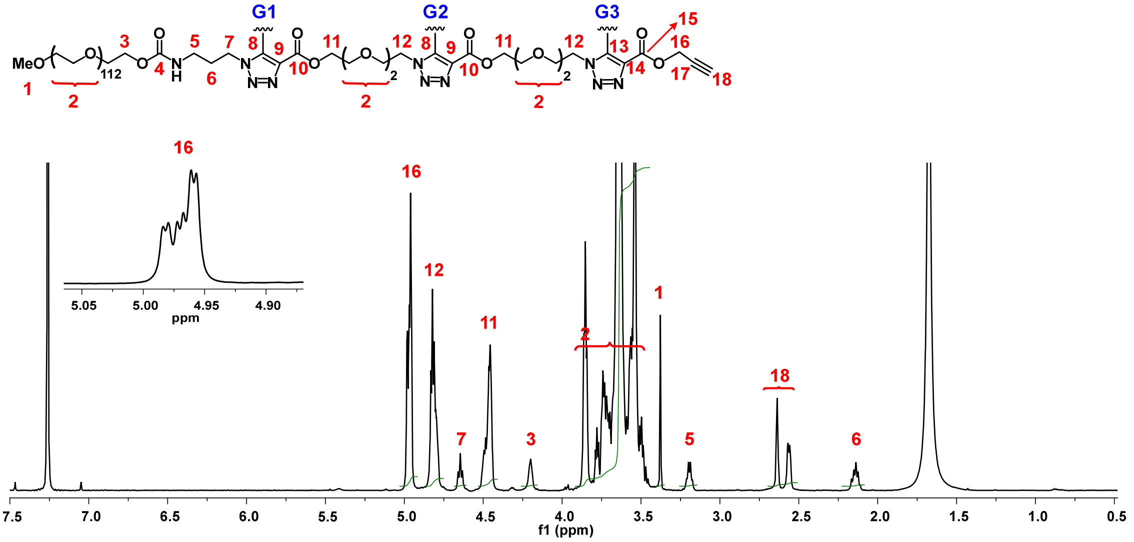

^{1}H NMR spectrum of PEG-[G3]-yne ($CDCl_3$)

Because of the appearance of a small signal at 2130 cm^{-1} in the IR spectrum of PEG-[G3]-yne (due to the presence of the terminal alkyne) that interferes with the intense band at 2100 cm^{-1} typical of azides, monitoring of this AAC functionalization was also done by ^{1}H NMR as shown below.

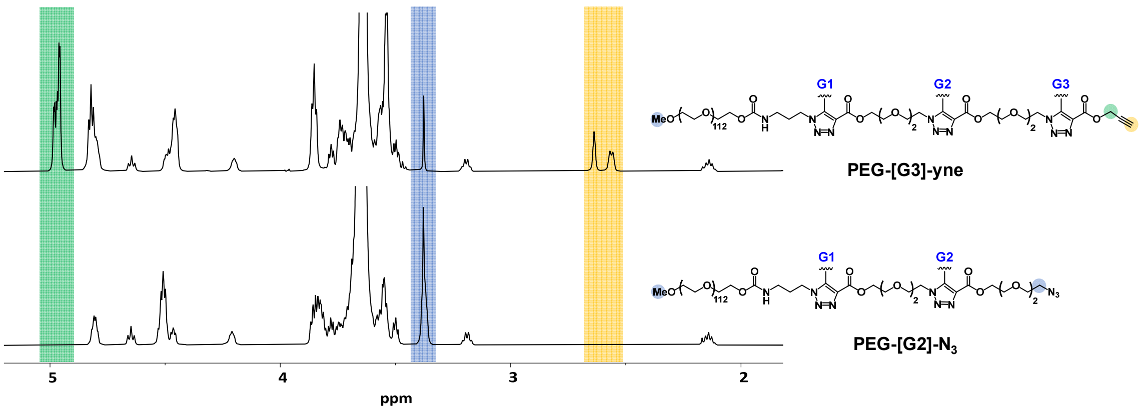

Monitoring of complete PEG-[G3]-yne formation by ^{1}H NMR ($CDCl_3$)

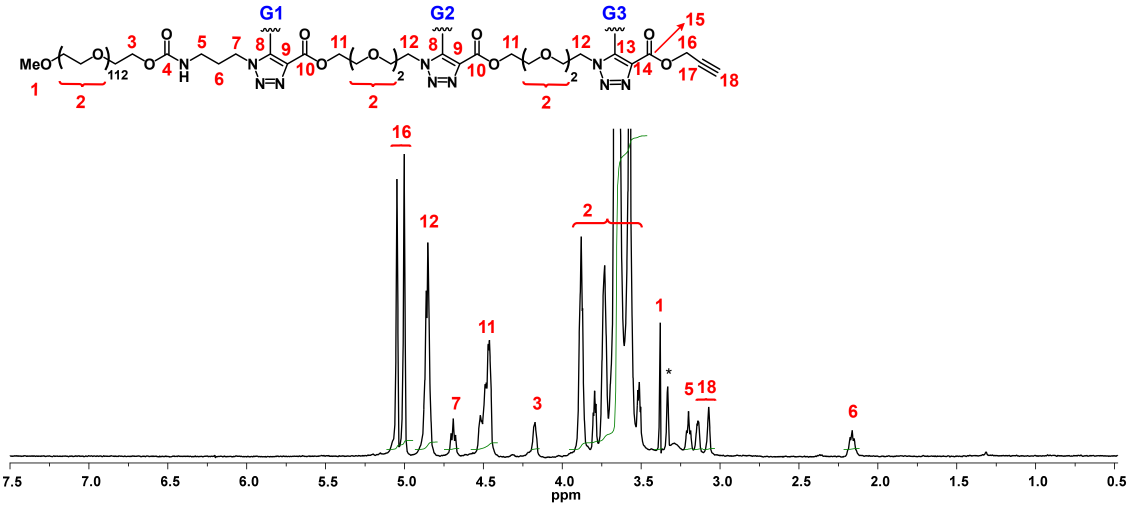

^{1}H NMR spectrum of PEG-[G3]-yne (Diffusion filter 50 ms, CD_3OD)

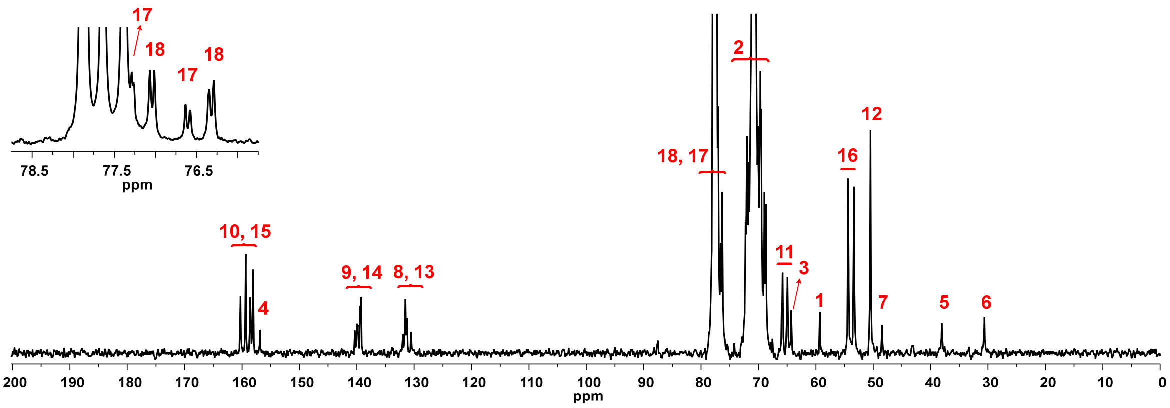

^{13}C NMR spectrum of PEG-[G3]-yne (CDCl$_3$)

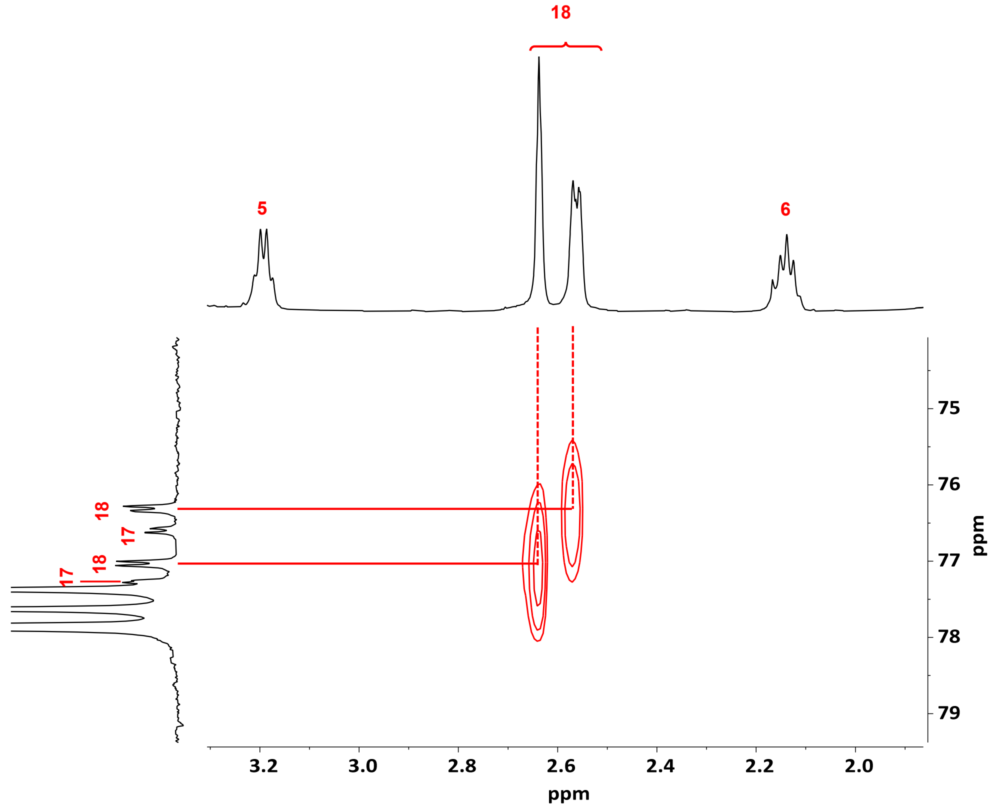

HSQC spectrum of PEG-[G3]-yne (CDCl$_3$)

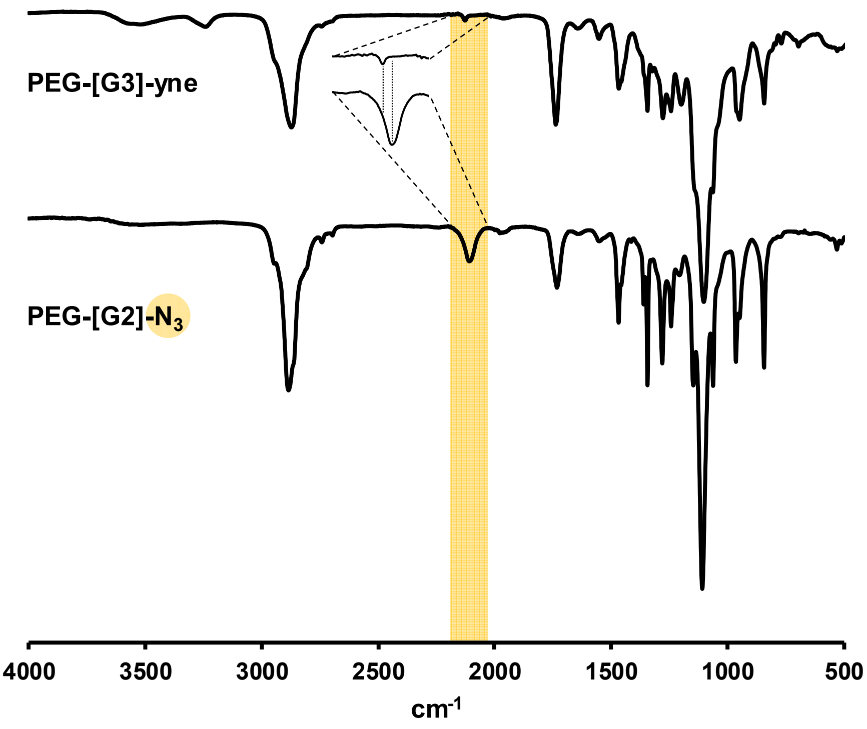

IR spectrum of PEG-[G3]-yne

PEG-[G3]-Gal. A solution of PEG-[G3]-ene (100 mg, 0.014 mmol), 1-thio-β-D-galactose (65 mg, 0.33 mmol), and DMPA (3 mg, 0.011 mmol) in dry DMF (0.3 mL) was deoxygenated by bubbling Ar for 15 min. Then, it was irradiated with 350 nm UV light (Asahi Spectra Max-303 xenon lamp, 300 W) for 3 h. Afterwards, the reaction mixture was purified by ultrafiltration (YM3, MilliQ H_2O, 5 x 50 mL) to give PEG-[G3]-Gal (106 mg, 99%) as a white foam. ^1H NMR (500 MHz, D_2O) δ: 4.91 – 4.82 (m, 12H), 4.69 (t, J = 7.0, 2H), 4.60 – 4.44 (m, 36H), 4.22 – 4.18 (m, 2H), 3.97 – 3.56 (m, ~546H), 3.43 (s, 3H), 3.19 (t, J = 6.5, 2H), 2.99 – 2.79 (m, 16H), 2.22 – 2.09 (m, 18H). ^{13}C NMR (126 MHz, D_2O) δ: 161.1, 160.7, 159.2, 158.8, 158.6, 158.3, 139.6, 139.3, 131.9, 131.3, 86.4, 79.3, 74.4, 71.4, 71.2, 71.1, 70.8, 70.0, 69.3, 68.6, 68.4, 66.6, 65.6, 65.2, 64.3, 63.6, 61.4, 58.5, 50.9, 49.2, 37.9, 29.7, 28.9, 26.9. IR (ATR, cm^{-1}): 2885, 1726, 1105.

Of note, as revealed in the HSQC spectrum, the copolymer displays a 5% of α-D-galactose anomer (5.42 ppm), which is already present in the commercial galactose reagent.

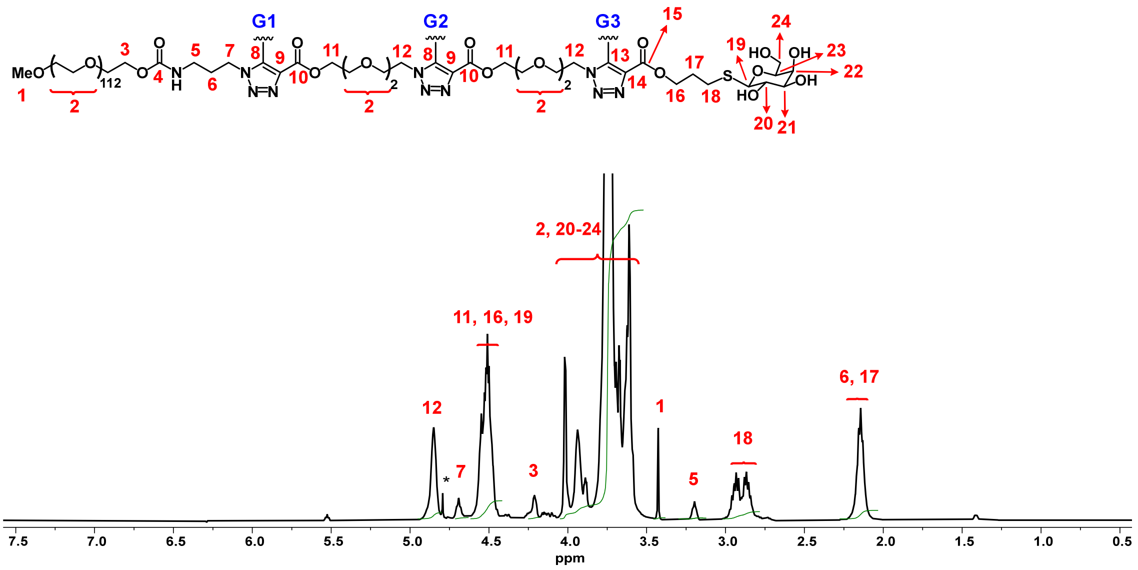

^1H NMR spectrum of PEG-[G3]-Gal (Diffusion filter 90 ms, D_2O)

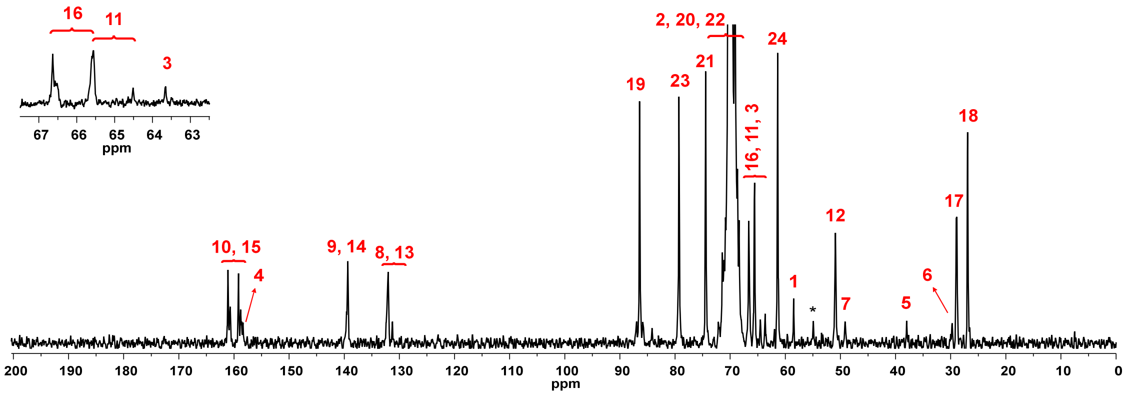

^{13}C NMR spectrum of PEG-[G3]-Gal (D_2O)

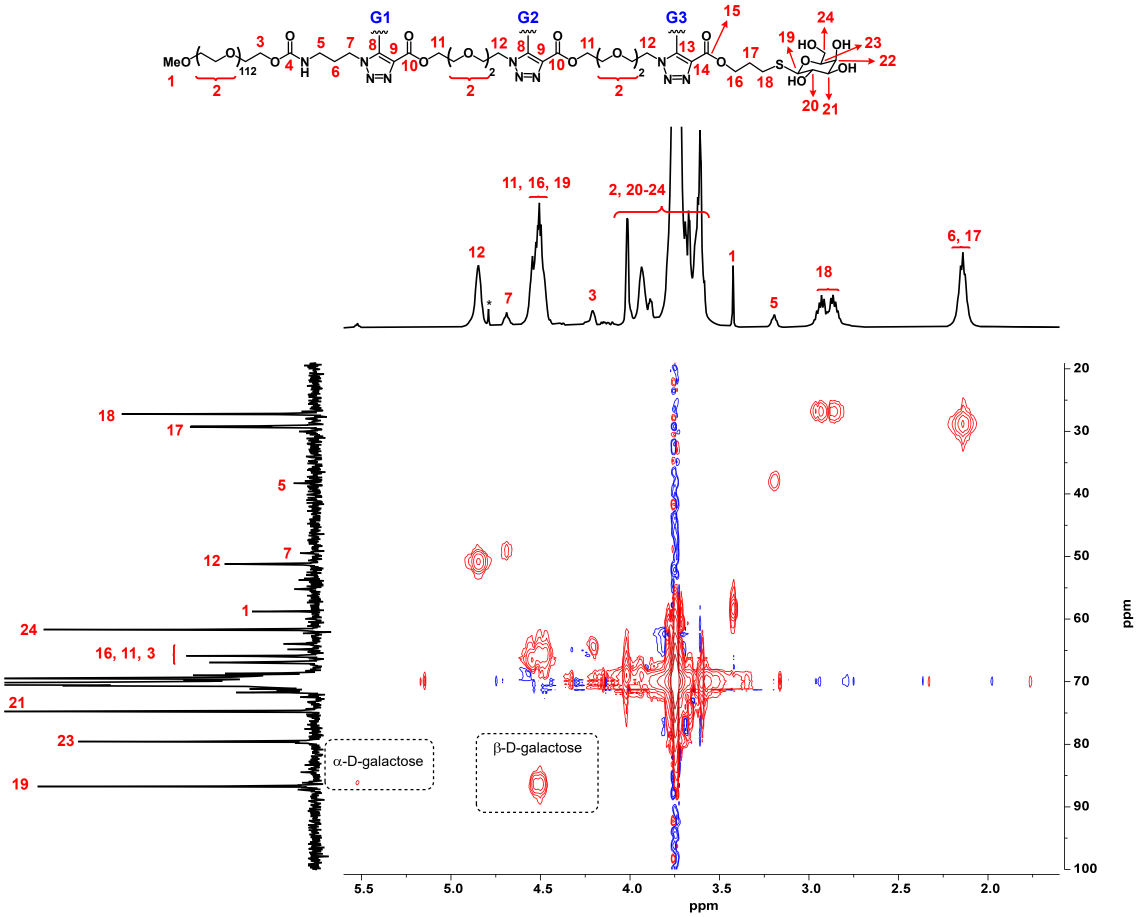

HSQC spectrum of PEG-[G3]-Gal (D_2O)

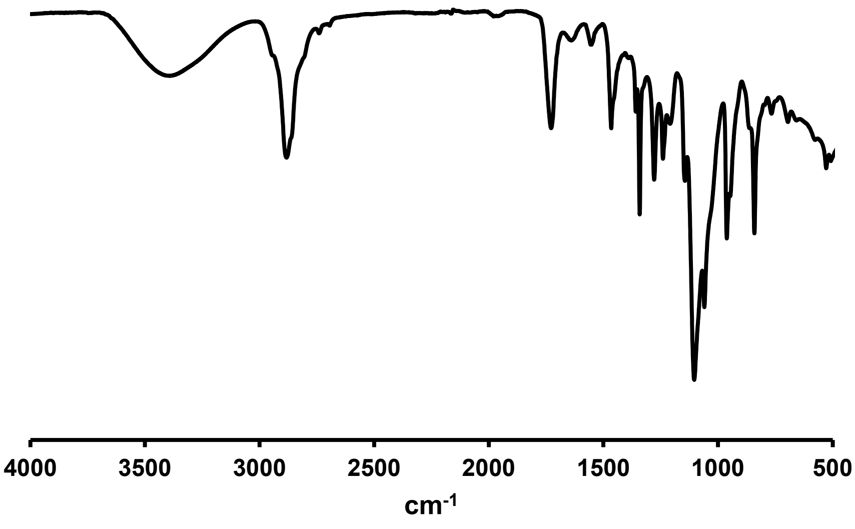

IR spectrum of PEG-[G3]-Gal

PEG-[G4]-Suc. A solution of PEG-[G3]-yne (217 mg, 0.03 mmol), 2-mercaptosuccinic acid (216 mg, 1.44 mmol), and DMPA (6 mg, 0.02 mmol) in dry DMF (0.7 mL) was deoxygenated by bubbling Ar for 15 min. Then, it was irradiated with 350 nm UV light (Asahi Spectra Max-303 xenon lamp, 300 W) for 3 h. Afterwards, the reaction mixture was concentrated and precipitated from MeOH/*i*PrOH (rt) to give PEG-[G4]-Suc (254 mg, 88%) as a white solid. ^{1}H NMR (500 MHz, CD_3OD) δ: 4.94 – 4.81 (m, 12H), 4.81 – 4.72 (m, 8H), 4.72 – 4.58 (m, 10H), 4.57 – 4.36 (m, 12H), 4.18 – 4.13 (m, 2H), 3.99 – 3.44 (m, ~506H), 3.37 (s, 3H), 3.29 – 3.03 (m, 10H), 3.01 – 2.85 (m, 12H), 2.83 – 2.64 (m, 12H), 2.19-2.11 (m, 2H). ^{13}C NMR (126 MHz, CD_3OD) δ: 175.5, 174.5, 161.9, 161.8, 161.4, 160.0, 159.7, 141.3, 140.9, 140.7, 133.6, 133.4, 133.3, 132.4, 73.6, 73.1, 72.0, 71.2, 70.9, 70.3, 70.0, 69.0, 68.0, 67.5, 66.5, 65.7, 59.6, 52.3, 46.1, 44.3, 43.5, 39.5, 38.1, 37.8, 36.0, 35.6, 35.2, 31.8. IR (ATR, cm^{-1}): 2877, 1725, 1104.

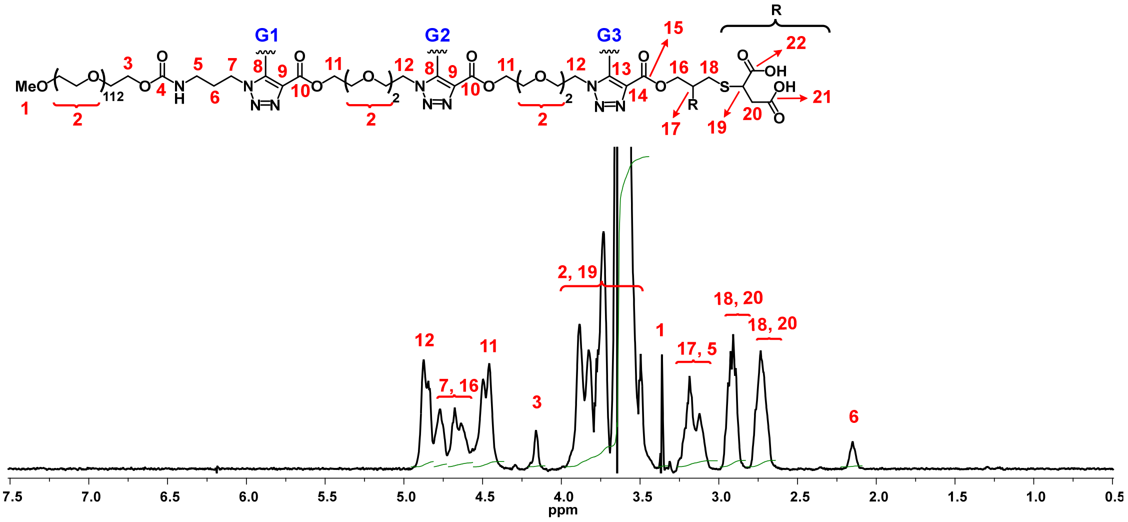

^{1}H NMR spectrum of PEG-[G4]-Suc (Diffusion filter 100 ms, CD_3OD)

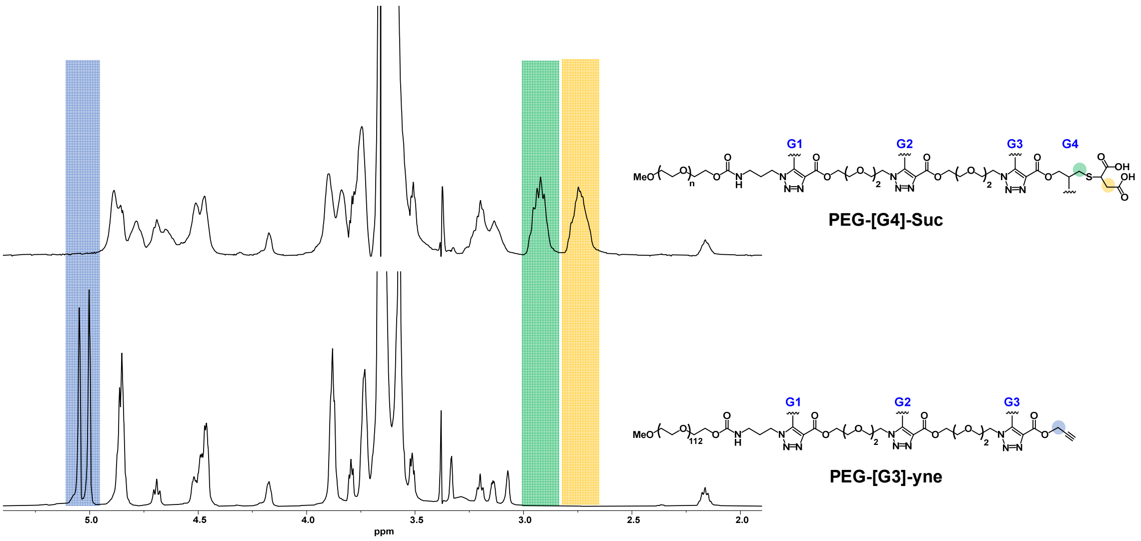

Monitoring of complete PEG-[G4]-Suc formation by ^{1}H NMR (CD_3OD)

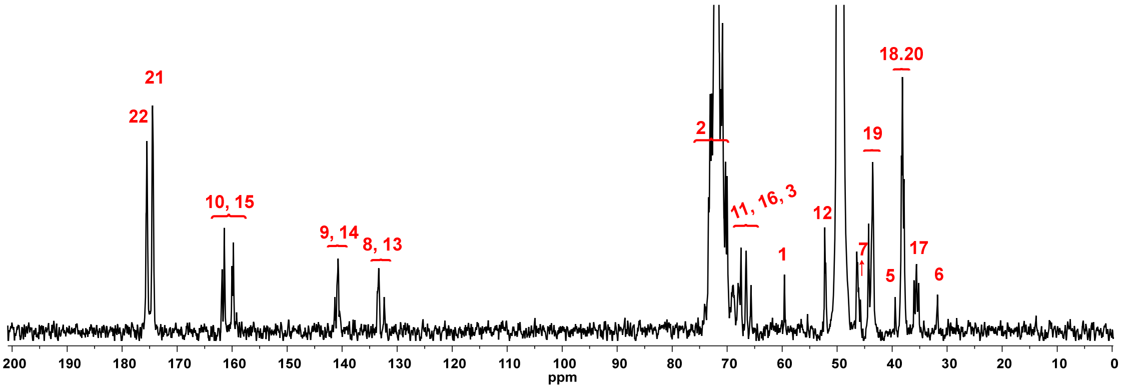

^{13}C NMR spectrum of PEG-[G4]-Suc (CD_3OD)

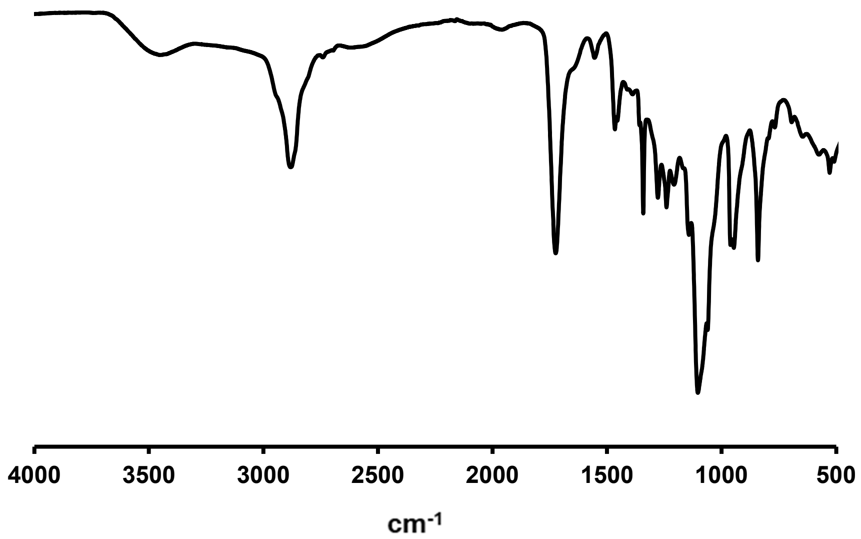

IR spectrum of PEG-[G4]-Suc

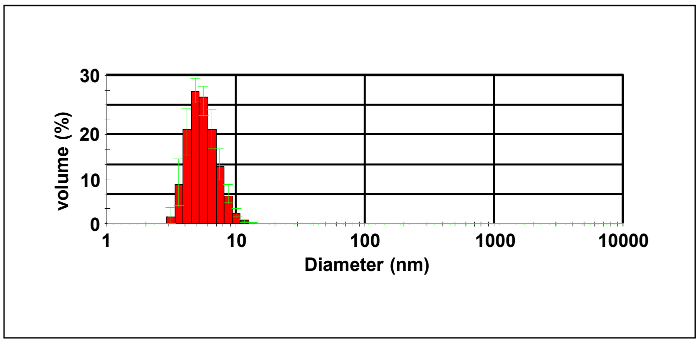

DLS histogram of PEG-[G4]-Suc (1 mg/mL in D_2O)

2. Degradability of the Dendritic Structure

To determine the rate of hydrolysis of the ester groups at the dendritic block under physiological pH conditions, PEG-[G3]-Gal (8 mg/mL) was incubated in deuterated 10 mM phosphate buffer PB (pH 7.4) at 37 ºC. The progress of hydrolysis was determined by ^{1}H NMR at different time points by integration of a multiplet between 4.62 and 4.43 ppm, corresponding to 36 protons: 28 alpha to the ester groups and 8 anomeric protons of galactose. Appearance of a new signal at 1.93 ppm during the experiment, assigned to 1-(3-hydroxypropyl)thio-β-galactose, allowed to quantify the extent of hydrolysis of the peripheral esters close to the galactose groups. The difference between both values unveiled the hydrolysis rate of the internal dendritic esters. Integration of the spectra was done relative to 3 protons at 3.43 ppm corresponding to the OMe signal of the PEG.

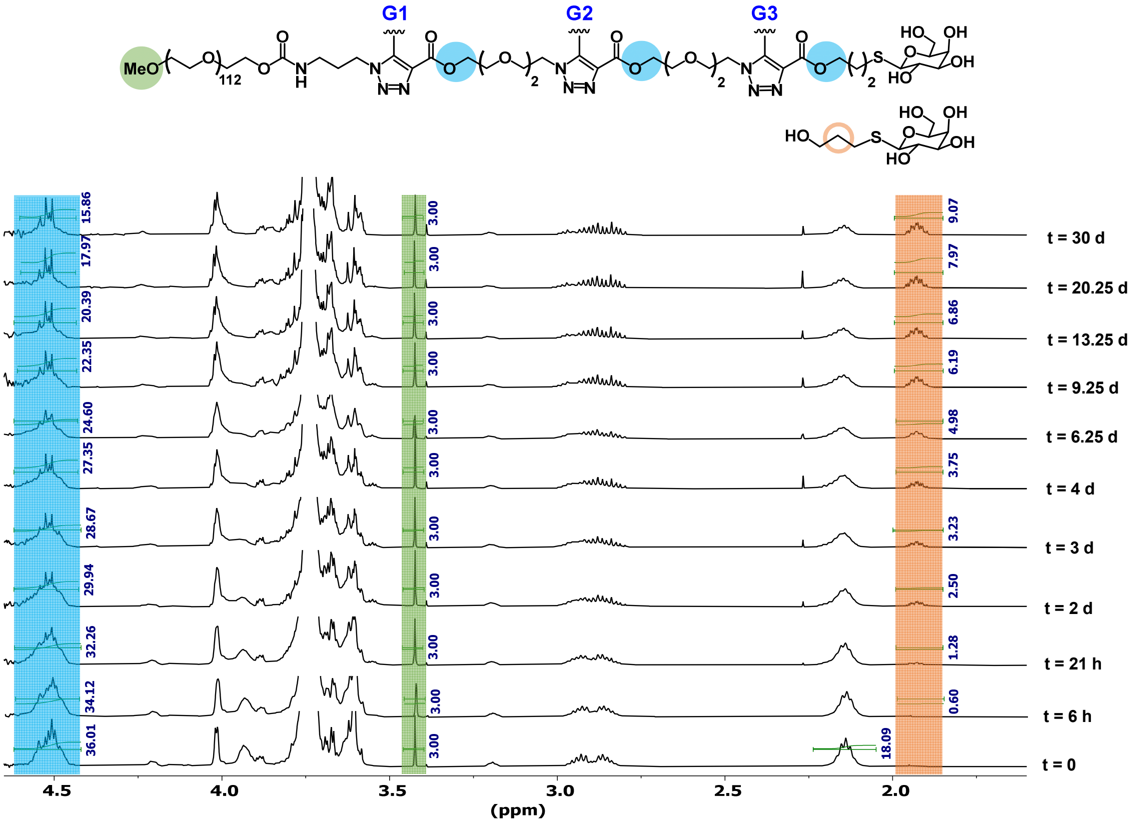

Figure S2. ^{1}H NMR spectra (500 MHz, D_2O, 10 mM PB, pD 7.0) of PEG-[G3]-Gal at different time points

3. Amphiphilic and Doxorubicin (DOX) Loaded Micelles

For the preparation of micelles from PEG-[G3]-Et, PEG-[G3]-Ar, and PEG-[G3]-Dod, copolymers were dissolved in MeOH at different concentrations (1, 4, and 8 mg/mL). Then, 10 mM PB pH 7.4, 150 mM NaCl was slowly added while vortexing (final volume ratio, 1:1). After evaporation of MeOH using a rotary evaporator, the formation of micelles was checked by DLS at 25 °C. Micelles with lower pdi were obtained on increasing the polymer concentration.

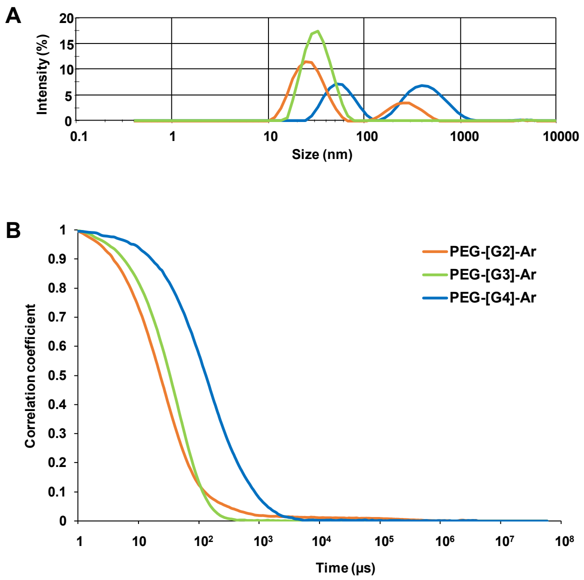

Figure S3. DLS size distributions (A) and correlation functions (B) of PEG-[G2]-Ar, PEG-[G3]-Ar, and PEG-[G4]-Ar assemblies (10 mM PB pH 7.4, 150 mM NaCl)

Critical micelle concentration (CMC) of PEG-[G3]-Ar and PEG-[G3]-Dod micelles:
The critical micelle concentration (CMC) of PEG-[G3]-Ar and PEG-[G3]-Dod micelles was determined using pyrene as a fluorescence probe. A 10^{-6} M pyrene solution in 10 mM PB pH 7.4, 150 mM NaCl was prepared as follows. 50 µL of a 2×10^{-2} M pyrene solution in CH_2Cl_2were added to a 1 L round-bottom flask. The solvent was evaporated,

and the flask was submitted to high vacuum for 1 h protected from light. Then, 1 L of a 10 mM PB pH 7.4, 150 mM NaCl solution was added and stirred for 72 h. Micelles from PEG-[G3]-Ar and PEG-[G3]-Dod were prepared as described above using this buffer (10^{-6} M pyrene) at a concentration of 8 mg/mL. These solutions of micelles were subsequently diluted with the same buffer down to 5×10^{-4} mg/mL of copolymers. Fluorescence emission spectra of the resulting solutions were obtained by exciting samples at 333 nm and measuring the emission between 350 and 500 nm. The intensity ratios between excimer (I_E, 450 nm) and monomer (I_M, 371 nm) were plotted vs the logC. The CMC results from the intersection between the regression straight lines of the linearly dependent region and the lower horizontal portion of the curve.

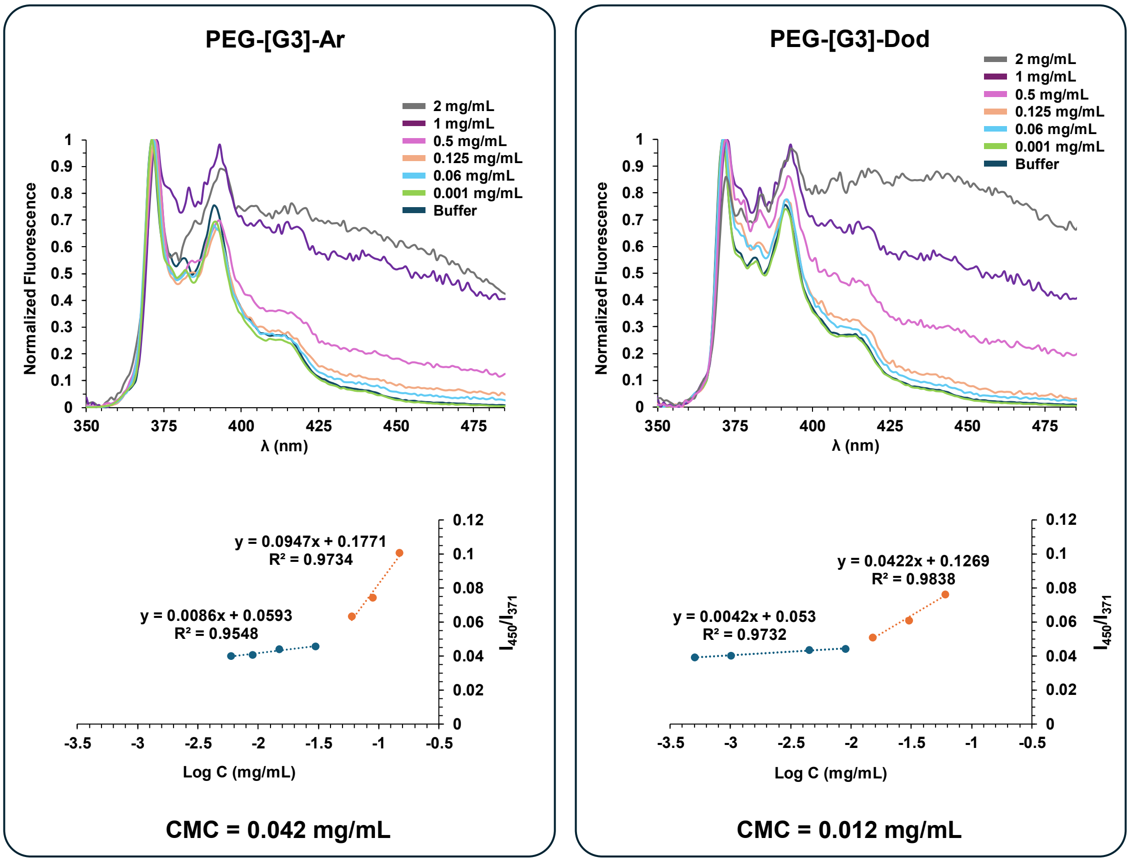

Figure S4. CMC determination of PEG-[G3]-Ar and PEG-[G3]-Dod micelles

Encapsulation efficiency (EE) and drug loading (DL)

- Encapsulation efficiency (EE) describes the fraction of drug incorporated into a micelle compared to the total amount of drug used in its preparation (EE = weight of drug in micelle × 100/weight of feeding drug).
- Drug loading (DL) refers to the mass fraction of a micelle that is composed of the drug (DL = weight of drug in micelle × 100/weight of drug-loaded micelle).

DOX encapsulation. Aqueous solutions of doxorubicin hydrochloride (0.75 mL, 1.79 mg/mL in 150 mM NaCl) were added to freshly prepared solutions of PEG-[G3]-Ar and PEG-[G3]-Dod micelles (0.75 mL, 8 mg/mL in 10 mM PB pH 7.4, 150 mM NaCl). The amount of DOX accounts for 40 mol% of the dendrimer peripheral Ar or Dod groups. After incubation for 48 h at 37 ºC, unloaded DOX was removed by dialysis (Spectra/Por 6, MWCO 1 KDa) against 200 mL PBS for 6 h in the dark at 37 ºC. To determine the EE and DL of DOX, aliquots of the buffer solution were taken at the end of the dialysis (3 × 150 µL) and placed in a 96-well microplate. Then, 500 mM acetate buffer pH 4 (50 µL) was added to each well and the fluorescence of the samples was measured in a microplate reader (exc. 485±20 nm, em. 535±20 nm; Tecan Infinite F200 PRO). The concentration of unloaded DOX in solution was determined by comparison with a standard calibration curve made from the fluorescence emission of fresh solutions of DOX of known concentrations prepared under identical conditions. An EE of 80% and DL of 19% were obtained for the PEG-[G3]-Dod micelles.

In vitro release study of DOX. DOX-loaded PEG-[G3]-Dod micelles were prepared and dialyzed as described above (Spectra/Por 6, MWCO 1 KDa; 800 µL of micelles against 200 mL PBS, 6 h, 37 °C, in the dark). Then, dialysis bags were transferred to new buffer solutions: (i) 200 mL of PBS and (ii) 200 mL of 50 mM acetate buffer pH 5.0, 100 mM

NaCl, and dialysis continued at 37 °C in the dark. At fixed times, aliquots (3 × 100 μL) were taken and placed in 96-well microplates (Nunc F96 MicroWell flat black from Thermo Scientific). Then, 500 mM acetate buffer pH 4 (100 μL) was added to each well and the fluorescence of the samples was measured in a microplate reader (exc. 485±20 nm, em. 535±20 nm; Tecan Infinite F200 PRO). The amount of released DOX was calculated by comparison with a standard calibration curve made from the fluorescence emission of fresh solutions of DOX of known concentration, prepared under identical conditions.

Cell cultures. Human adenocarcinoma alveolar basal epithelial (A549) cells, obtained from the European Collection of Authenticated Cell Cultures (ECACC), were cultured at 37 °C in 5% CO_2 atmosphere in Dulbecco's modified Eagle's medium (DMEM) with high glucose, containing 10% fetal bovine serum (FBS) and supplemented with 50 U/mL penicillin and 50 U/mL streptomycin. All cell experiments were performed with this modified DMEM containing 10% FBS and simply referred in the text as "medium".

Cytotoxicity of PEG-[G3]-Et, PEG-[G3]-Ar, and PEG-[G3]-Dod micelles. Micelles from PEG-[G3]-Et, PEG-[G3]-Ar, and PEG-[G3]-Dod were prepared as described above at a final concentration of 8 mg/mL in 10 mM PB pH 7.4, 150 mM NaCl. These solutions were diluted with medium to reach final copolymer concentrations between 60 and 1.25 mM (Table S1). A-549 cells were seeded in 96-well plates at a density of 80000 cells/mL. After 24 h of incubation at 37 °C in 5% CO_2, medium was replaced with the copolymer solutions (100 μL). After 48 h of incubation, cell viability was determined by a colorimetric assay with CCK-8 following manufacturer's protocol. After 2 h of incubation with a 6% solution of CCK-8 in medium, supernatant solution was transferred to a 96-well plate. Viability was determined by measuring the supernatant absorbance at 450 nm in a plate reader Tecan Infinite F200 PRO. Absorbance (A) from a 6% CCK-8

solution in medium was subtracted from all data points. Viability was calculated as follows (results show the average and standard deviation of three independent experiments):

$$\text{Cell Viability (\%)} = 100 \times \frac{(A_{\text{sample}} - A_{6\%\ \text{CCK-8}})}{(A_{\text{control}} - A_{6\%\ \text{CCK-8}})}$$

Table S1. Concentrations of copolymers analyzed in the cytotoxicity study.

Copolymer (mM)	PEG-[G3]-Et (µg/mL)	PEG-[G3]-Ar (µg/mL)	PEG-[G3]-Dod (µg/mL)
**60**	496	478	429
**40**	330	319	286
**30**	248	239	214
**20**	165	160	143
**15**	124	120	107
**10**	82	80	71
**5**	41	40	36
**2.5**	21	20	18
**1.25**	10	10	9

Cellular uptake studies. Human lung adenocarcinoma cells (A549) were seeded in 4-chamber glass bottom 35 mm dish with 20 mm bottom well from In Vitro Scientific in 0.5 mL high glucose Dulbecco's Modified Eagle's Medium (DMEM) supplemented with 10% FBS and 1% antibiotics solution (10000 U/mL penicillin, 10000 mg/mL streptomycin, and 29.2 mg/mL glutamine) and incubated overnight at 37 °C in 5% CO_2 atmosphere. Then, medium was replaced by a solution of 30 µL of DOX-loaded PEG-[G3]-Dod micelles in 470 µL of medium, and incubation was continued at 37 ºC for 30 min. Afterwards, medium was removed, and fresh medium was added to the cell culture plates. Cells were left incubating at 37 ºC for 1 h or 2 h. Half an hour before observation,

acidic organelles were stained by adding 10 μL of 10 μM LysoTracker Green and nuclei were stained by adding 10 μL of 200 μM Hoechst 33258. Before observation, medium was replaced with fresh medium.

The intracellular distribution of DOX was studied in a Laser Scanning Confocal Inverted Microscopy Leica Stellaris 8 FALCON (Leica Microsystems, Wetzlar, Germany) equipped with Leica Application Suite X (LAS X) package. Excitation / emission wavelengths were 405 nm / 415-482 nm for Hoechst 33258; 490 nm / 500-540 nm for LysoTracker Green; and 440 nm / 580-650 nm for Doxorubicin. Hybrid detectors were used for detecting fluorescence signal and a PMT for capturing brightfield images. Images were acquired applying a sequential method with a 1296 x 1296 pixels resolution (82 μm × 82 μm). The glycerol immersion employed objective was HC PL APO CS2 63x/1.30 Gly. Finally, resolution was optimized by applying Leica Lightning adaptative deconvolution.

4. Membrane Crosslinked Polymersomes. PEG-[G2]-N_3 (4 mg, 619 nmol) was dissolved in acetone:H_2O (1:1, 2 mL) and stirred at room temperature for 22 h until acetone was completely evaporated. The resulting solution of polymersomes (4 mg/mL in H_2O) was analyzed by DLS (Figure 5A) and divided in two portions (0.5 mL each). One of the portions was freeze-dried and then, resuspended in acetone (0.5 mL) and analyzed by DLS to unveil the presence of free block copolymer in solution (Figure 5B). The second portion of polymersomes (2 mg, 309 nmol, 4 mg/mL in H_2O) was treated with bis-dPEG$_{11}$-DBCO (212 µg, 42 µL of a 5 mg/mL solution in acetone, 0.15 equiv. per azide) and stirred at rt for 8 h. DLS analysis confirmed the presence of crosslinked polymersomes with nearly identical size and pdi than before crosslinking (Figure 5C). When this sample was freeze-dried and resuspended in acetone (0.5 mL), DLS corroborated a retention of the assembly structure imparted by the SPAAC crosslinking (Figure 5D).

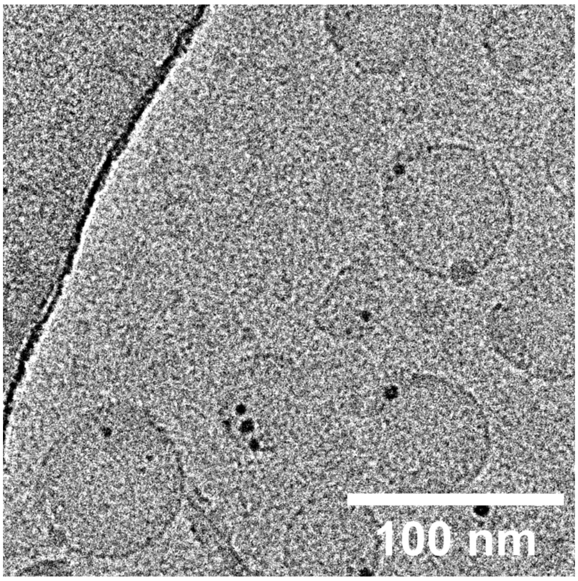

Figure S5. Cryo-TEM image of polymersomes from PEG-[G2]-N_3

5. PIC and Hybrid-PIC

PIC with Poly-Arg. PEG-[G4]-Suc (0.39 mg/mL) was dissolved in 10 mM Na_2HPO_4. Poly-Arg (DP 144) (0.5 mg/mL) was dissolved in 10 mM NaH_2PO_4/0.1 M HCl (10% v/v). Both solutions were aged at rt overnight and filtered through 0.45 μm nylon filters immediately before PIC formation. PIC micelles were prepared by adding the PEG-[G4]-Suc solution over the Poly-Arg solution (volume ratio 2:1, which accounts for a stoichiometric charge ratio). Micelles were aged for 1 h at rt and then, were dialyzed against 10 mM PB pH 7.4 for 45 min and 10 mM PB pH 7.4, 150 mM NaCl for 90 min. Finally, the stability of the micelles was studied by heating at 37 ºC for 24 h.

Hybrid-PIC with Ca^{2+}. A standard solution of $CaCO_3$ (66.60 μL, 1.66 μmol of Ca^{2+}, 16 equiv. of Ca^{2+} per PEG-[G4]-Suc, 1 mg Ca/mL determined by ICP) was added to a solution of PEG-[G4]-Suc (933.4 μL, 0.104 μmol, 1.071 mg/mL in 50 mM Na_2HPO_4). The resulting solution (pH 7.8) was stirred at rt for 24 h and then, dialyzed against MilliQ H_2O (3 × 250 mL) to afford HPIC that were analyzed by DLS, cryo-TEM, and SEM.

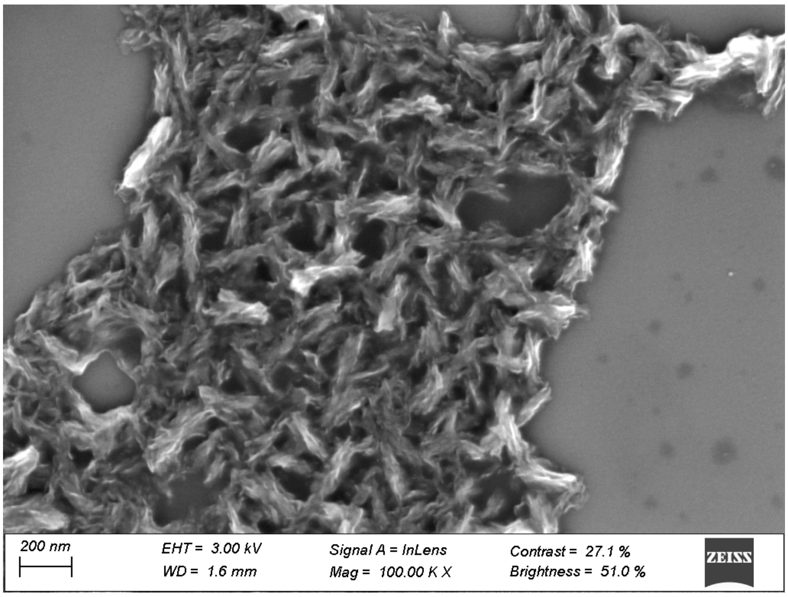

Figure S6. SEM image of HPIC from PEG-[G4]-Suc and Ca^{2+}

Covalently crosslinked HPIC were obtained by adding ethylenediamine (5 µL of a 0.066 M solution in MilliQ H_2O, 0.332 µmol, 3.2 equiv. per PEG-[G4]-Suc) and EDC (9.6 µL of a readily prepared solution of 100 mg of EDC in 1 mL of MilliQ H_2O, 4.99 µmol, 48 equiv. per PEG-[G4]-Suc) to the above HPIC solution. The resulting mixture was allowed to stir overnight. The efficiency of the covalent crosslinking was assessed by comparing the DLS histograms of HPIC samples treated with EDTA (16.6 µL, 1.66 µmol, 0.1 M in MilliQ H_2O, pH 6; 1 equiv. per Ca^{2+}) before and after crosslinking.

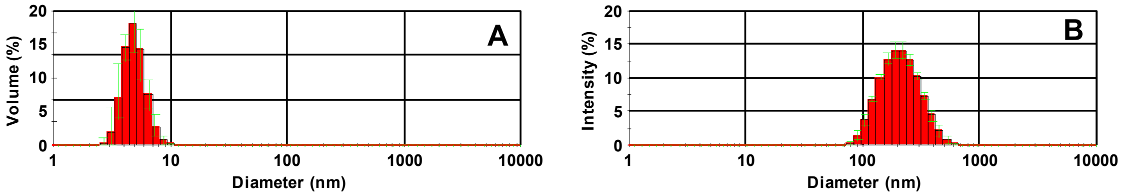

Figure S7. DLS histograms of HPIC (PEG-[G4]-Suc/Ca^{2+}) treated with EDTA before (A) and after (B) crosslinking
